# A MiR181/Sirtuin1 regulatory circuit modulates drug response in biliary cancers

**DOI:** 10.1007/s10238-024-01332-0

**Published:** 2024-04-10

**Authors:** Anna Barbato, Fabiola Piscopo, Massimiliano Salati, Carla Pollastro, Lorenzo Evangelista, Luigi Ferrante, Davide Limongello, Simona Brillante, Antonella Iuliano, Luca Reggiani-Bonetti, Maria Salatiello, Antonino Iaccarino, Pasquale Pisapia, Umberto Malapelle, Giancarlo Troncone, Alessia Indrieri, Massimo Dominici, Brunella Franco, Pietro Carotenuto

**Affiliations:** 1 TIGEM, Telethon Institute of Genetics and Medicine, Via Campi Flegrei 34, 80078 Pozzuoli, Naples, Italy; 2 Division of Oncology, Department of Oncology and Hematology, University Hospital of Modena, 41125, Modena, Italy; 3 Department of Mathematics, Computer Science and Economics (DIMIE), University of Basilicata, 85100 Potenza, Italy; 4 Department of Medical and Surgical Sciences for Children and Adults, University Hospital of Modena, 41125 Modena, Italy; 5 Department of Public Health, Universita’ degli Studi di Napoli—AOU Federico II, 80131 Naples, Italy; 6 Department of Translational Medical Science, Medical Genetics, University of Naples “Federico II”, 80131 Naples, Italy; 7 IRGB, Institute for Genetic and Biomedical Research, National Research Council (CNR), Milan, Italy; 8 Scuola Superiore Meridionale (SSM, School of Advanced Studies), Genomics and Experimental Medicine Program, 80078 Naples, Italy

**Keywords:** Biliary tract cancer, miR-181, MicroRNA, Biomarkers, Cancer resistance, Cancer genomics

## Abstract

Despite recent advances, biliary tract cancer (BTC) remains one of the most lethal tumor worldwide due to late diagnosis, limited therapeutic strategies and resistance to conventional therapies. In recent years, high-throughput technologies have enabled extensive genome, and transcriptome sequencing unveiling, among others, the regulatory potential of microRNAs (miRNAs). Compelling evidence shown that miRNA are attractive therapeutic targets and promising candidates as biomarkers for various therapy-resistant tumors. The analysis of miRNA profile successfully identified miR-181c and -181d as significantly downregulated in BTC patients. Low miR-181c and -181d expression levels were correlated with worse prognosis and poor treatment efficacy. In fact, progression-free survival analysis indicated poor survival rates in miR-181c and -181d low expressing patients. The expression profile of miR-181c and -181d in BTC cell lines revealed that both miRNAs were dysregulated. Functional in vitro experiments in BTC cell lines showed that overexpression of miR-181c and -181d affected cell viability and increased sensitivity to chemotherapy compared to controls. In addition, by using bioinformatic tools we showed that the miR-181c/d functional role is determined by binding to their target SIRT1 (Sirtuin 1). Moreover, BTC patients expressing high levels of miR-181 and low SIRT1 shown an improved survival and treatment response. An integrative network analysis demonstrated that, miR-181/SIRT1 circuit had a regulatory effect on several important metabolic tumor-related processes. Our study demonstrated that miR-181c and -181d act as tumor suppressor miRNA in BTC, suggesting the potential use as therapeutic strategy in resistant cancers and as predictive biomarker in the precision medicine of BTC.

**Supplementary Information:**

The online version contains supplementary material available at 10.1007/s10238-024-01332-0.

## Introduction

Biliary Tract Cancers (BTCs) are a heterogeneous group of aggressive solid tumors arising from the malignant transformation of the epithelial cells facing the lumen of the biliary tree. BTCs includes a cluster of different biliary tumors classified into three subtypes, according to their anatomic location: intrahepatic, perihilar and distal cholangiocarcinoma (CCA) and gallbladder cancer (GBC) [1]. BTCs represent the second most common primary hepatic malignancy after hepatocellular carcinoma, accounting for 10–15% of all liver cancers and approximately 3% of all gastrointestinal tumors [2, 3]. Their incidence is increasing globally mainly as a result of a rise in intrahepatic CCA cases. For resectable cases, surgery followed by 6-month adjuvant chemotherapy is the current standard of care endorsed by international guidelines [4], while for the advanced disease the association of PDL1 inhibitors to cisplatin/gemcitabine have changed the first-line treatment paradigm [4]. Although surgery is a potential curative option, only approximately 30% of all BTC patients is suitable for this therapeutic approach. Conventional chemotherapy is generally ineffective, and patients develop resistance to palliative treatments [4].

In the past few years, the advent of high-throughput technologies namely next-generation sequencing has enabled the identification of several druggable molecular vulnerabilities in BTC. Among them, mutations in isocitrate dehydrogenase 1 gene (IDH1) [5] and fusions/rearrangements of the fibroblast growth factor receptor 2 gene (FGFR2) [6, 7, 8] have been the most successfully exploited in clinical trials with currently three targeted agents approved for pretreated advanced CCA harboring these genomic aberrations. Despite latest genomic-driven progress, only a minority of patients may benefit from targeted therapies and even in responding patients acquired resistance almost invariably occurs leading to a meager overall survival in advanced BTC.

These observations highlighted the urgent unmet need for a better understanding of tumor biology, particularly of non-genetic molecular mechanisms in order to identify novel biomarkers predicting prognosis and treatment efficacy as well as novel therapeutic targets.

During the last decade, many studies have focused on the potential regulatory role of non-coding RNA (ncRNA) in human cancer [9, 10]. MicroRNAs are endogenous small non-coding RNAs that play a pivotal role in regulating gene expression. MiRNAs may function as either oncogenes or tumor suppressors depending on the tumor type. Recent evidence shown that dysregulated miRNAs can affect the hallmarks of cancer, including proliferation, survival, invasion and metastasis, angiogenesis and drug resistance [11]. Additionally, an increasing number of studies have identified miRNAs as potential new biomarkers for human cancer. Compelling evidence have demonstrated that miRNAs are dysregulated in BTC and promote cholangiocarcinogenesis and tumor progression [12].

The miR-181 family belongs to a conserved group of miRNAs regulating cell proliferation, apoptosis, autophagy, mitochondrial function, and immune response [13, 14]. The miR-181 family is composed of four members: miR-181a, miR-181b, miR-181c and miR-181d, located into genetic clusters on chromosomes 1, 9 and 19. Several studies have demonstrated that miR-181 family members have a fundamental role in tumor progression and drug resistance [15, 16]. In fact, several evidence have shown that miR-181a and miR-181b are able to modulate different relevant biological processes by regulating targets involved in cancer-associated pathways in several human cancers [17, 18]. However, little is known regarding the other two members, the miR-181c and miR-181d [19, 20].

In this study, an exhaustively analysis of miRNA expression profiles of BTC patients and cell lines led us to identify miR-181c and -181d as significantly dysregulated in both human samples and in vitro models of BTC. We found that low miR-181c/d expression was associated with poor prognosis and lack of efficacy of treatments in BTC patients. Our study revealed the potential role of miR-181c/d as tumor suppressor in BTC and support the hypothesis that this might be exploited for new therapeutic approaches. Indeed, overexpression of miR-181c/d reduced cell growth and increased sensitivity to chemotherapy. We demonstrated that the miR-181c/d functional role is determined by binding to their target SIRT1 (Sirtuin 1). Based on the proposed integrative network, the interplay between miR-181c/d and SIRT1 had a negative regulatory effect on several important metabolic-related pathways modulating drug resistance in BTC. Overexpression of both miR-181c and miR-181d may represent innovative therapeutic tools to ameliorate the clinical management of BTC patients. Furthermore, we suggest that expression levels of miR-181c/d may be a useful biomarker to monitor and predict response to treatment in BTC patients.

## Materials and methods

### Human samples

The human BTC tissues were collected under approval of the Ethical Committee for Clinical Research at Azienda Ospedaliera Universitaria, Modena, Italy (ID 465/2018/SPER/AOUMO). The study protocols conformed to the ethical guidelines of the 1975 Declaration of Helsinki, as per ethical approval given by the institutional review board. Written informed consent was obtained from all participants. Diagnosis of BTC was established on radiological findings and was pathologically proven in all patients. Inclusion Criteria, Subject Demographics, Sex and Biological Variables and Clinicopathological characteristics were shown in Table S1.

Disease recurrence was defined as the presence of imaging‐proven disease.

Total RNA was extracted from the FFPE (Formalin-Fixed-Paraffinn-Embedded) samples from 62 tumor and the matched nontumor component after microscopic dissection (Table S1). MiRNAs were extracted from FFPE tissues before the beginning of therapy and at disease progression through Maxwell® RSC RNA FFPE Kit (Promega, Madison, WI, USA) and processed with Maxwell® RSC Instruments (Promega, Madison, WI, USA) according to the manufacturer’s instructions [21, 22].

### Cell culture and transfections

EGI-1, RBE and HUCCT1 cells were obtained from DSMZ (Braunschweig, Germany) and American Type Culture Collection (ATCC) (Manassas, VA, USA) and cultured in DMEM and RPMI 1640 medium (Invitrogen, Karlsruhe, Germany) with L-Glutamine, 10% fetal bovine serum (FBS; Invitrogen, Carlsbad, CA, USA), 100 U/mL penicillin and 50 µg of streptomycin, at 37 ◦C in humidified 5% CO2 atmosphere. H69 cells (obtained by Lonza, Siena, IT), an SV40-transformed (i.e. immortalized) normal human cholangiocyte cell line were grown in media containing DMEM/F12 (Sigma-Aldrich, St. Louis, MO) supplemented with fetal bovine serum (CellGro, Manassas, VA), 10% fetal bovine serum (FBS; Invitrogen, Carlsbad, CA, USA), 100 U/mL penicillin and 50 µg of streptomycin, at 37 ◦C in humidified 5% CO2 atmosphere. Gemcitabine- and Cisplatin-resistant cells (EGI-DR) were obtained by growing EGI cell lines in medium containing increased concentrations of Gemcitabine and Cisplatin for 4 weeks. EGI cell lines resistant to Gemcitabine 0,3 µM plus Cisplatin 3 µM were selected. Gemcitabine and Cisplatin were purchased from Sigma-Merck KGaA (Darmstadt, Germany). Authentication of cell lines was done by Eurofins (Milan, Italy) and confirmed by online STR-matching analysis (www.dsmz.de/fp/cgi-bin/str.html). SiRNA against SIRT1, MiR-181c and -181d mimics, inhibitors and negative controls were purchased from Dharmacon (Lafayette, CO, USA). Transfection with 40 nM mimics or inhibitors and/or negative control mimics was performed with Lipofectamine 2000 reagent (Invitrogen, Karlsruhe, Germany).

### RNA extraction and quantitative reverse transcription PCR

Total RNA was extracted from the cell samples using Trizol reagent (Invitrogen, California, CA, USA) or in alternative Maxwell RSC simplyRNA Cells (Glomax Discover System, Promega, Madison, WI, USA) and processed with Maxwell RSC Instrument (Glomax Discover System, Promega, Madison, WI, USA) following manufacturer’s instructions [21]. Single-stranded complementary DNA (cDNA) was generated using High Capacity cDNA Reverse Transcription Kit (Life Technologies, Paisley, UK). Quantitative Reverse Transcription PCR (qRT-PCR) was performed in LightCycler 96 (Roche, Penzberg, Germany) using LightCycler FastStart DNA Master SYBR Green I (Roche, Penzberg, Germany) and each validated primer. Validated qRT-PCR primers of SIRT1, GAPDH and ACTB were purchased from Eurofins (Milan, Italy).

QRT-PCR for miRNA was performed using a TaqMan MicroRNA assay kit (Applied Biosystems, Foster City, CA, USA) and specific primer sets for U6 snRNA (Assay ID: 001973), mature miR-181c (Assay ID: 000482), miR-181d (Assay ID: 001098), (Applied Biosystems, Foster City, CA, USA) according to the manufacturer’s instructions [22]. Reverse transcription was performed with TaqMan microRNA reverse transcription kit (Life Technologies, Paisley, UK), and microRNA expression assessed by qPCR with TaqMan assay (Life Technologies, Paisley, UK). We used ACTB and U6 snRNA as internal normalizers for mRNA and miRNA, respectively.

The primer sequences are as follows:

SIRT1: REV CTGCCACAAGAACTAGAGGATAAG; For AGTGGCAAAGGAGCAGATTAG.

GAPDH: REV AGGGGTGCTAAGCAGTTGGT; FOR ATGTTCGTCATGGGTGTGAA

ACTINB: REV GACTCCATGCCCAGGAGGG; FOR AAGAGCTACGAGCTGCCTGA

### Proliferation assay

MTS assays were performed using tetrazolium-based CellTiter 96 AQueous One Solution Cell Proliferation assay (Promega, Madison, WI, USA). Cells were seeded in 96-well plates at 10,000 cells per well for overnight incubation. Following adhesion of cells to the well, cells were treated with the experimental treatments indicated. Control groups were exposed to the same concentration of DMSO (Dimethyl sulfoxide; Sigma-Merck KGaA, Darmstadt, Germany). At the designated time-points, plates were read at the absorbance of 492 nm on a microplate reader (Glomax Discover System, Promega, Madison, WI, USA). Relative cell viability of an individual sample was calculated by normalizing their absorbance to that of the corresponding control. All experiments were done in triplicate. The GI50 was determined from the regression of a plot of the logarithm of the concentration versus percent inhibition by Prism GraphPad (GraphPad Software 8.0, La Jolla, CA, USA) using the Dose–Response One-Site Model.

### Colony formation assay

The cells were seeded onto 24-well plates (2000 cells/well) and treated with indicated compounds or vehicle control (Dimethyl sulfoxide, DMSO; Sigma-Merck KGaA, Darmstadt, Germany) for 7–10 days. After washing and fixation, the cells were stained with 0.5% Crystal Violet (Bio Basic Inc., Markham, Canada) in 25% methanol for 10 min. Cell colonies were then photographed and counted.

### Annexin V-FITC/PI staining assay

Cells were treated with indicated compounds and DMSO control for 48 h, and the cell apoptosis was measured by using Annexin V-FITC/PI Apoptosis Detection Kit (BD Biosciences, San Diego, CA, USA). Cells were suspended in binding buffer, stained with Annexin V-FITC and PI for 15 min at room temperature in dark. Apoptotic cells were analyzed using a BD Accuri C6 Analyzer (BD Biosciences, San Diego, CA, USA).

### Western blot analysis

The cells were lysed with RIPA buffer containing protease and phosphatase inhibitors (Selleckchem). The protein concentration was tested with a BCA kit, and appropriate amounts of protein were prepared for SDS-PAGE and then transferred to PVDF membrane (Millipore, MA, USA). The membranes were blocked for 1 h with 5% non-fat dry milk and then incubated with rabbit anti-SIRT1 (1:1000; #9475; Cell Signaling Technology Europe, Netherlands); TFAM (1:1000; #8076; Cell Signaling Technology Europe, Netherlands); CATALASE (1:1000; #12,980; Cell Signaling Technology Europe, Netherlands); SOD2 (1:1000; #MA1-106; Invitrogen); UQCRC2 (1:1000; #ab14745; Abcam); MTCO2 (1:1000; #ab110258; Abcam); ASCL4 (1:500; #sc-365230; Santacruz); Actin-b (1:5000, A5441; Sigma) mABs were used as loading controls. The results were imaged using a gel image analysis system (Bio-Rad, California, USA) according to the manufacturer’s instructions.

### RNAseq and small-RNAseq library preparation and deep sequencing

For RNA-seq analysis, libraries were prepared according to the manufacturer’s instructions (QuantSeq 3’ mRNA-Seq Library Prep Kit FWD for Illumina, Lexogen GmbH, Wien, Austria) starting from 250 ng of total RNA [16, 21]. Quality control of library templates was performed using a High Sensitivity D1000 Screen Tape (Agilent Technologies, Santa Clara, CA, USA) on a TapeStation 4200 (Agilent Technologies, Santa Clara, CA, USA). The Qubit quantification platform (Qubit 2.0 Fluorometer, Life Technologies/Thermo Fisher Scientific, MA, USA) was used to normalize samples for the library preparation. Using multiplexing, up to 87 samples were combined into a single lane to yield sufficient coverage. The sequencing was carried out in collaboration with Next Generation Diagnostic S.r.l. (Pozzuoli, Naples, Italy). Libraries were sequenced by single-end chemistry on an NovaSeq6000 platform (SP 100 cycles; Illumina, Cambridge, UK). Each library was loaded at a concentration of 250 pM, which was previously established as optimal. An average yield of ~ 4.5 Mb was obtained per sample.

Small RNA-Seq was performed by using a Small RNA-Seq Library Prep Kit (Lexogen, GmbH, Wien, Austria), according to manufacturer protocols. To verify the quantity and quality of RNA extracted before the library construction, the samples were tested using 2100 Bioanalyzer “total RNA pico bioanalyzer kit”(Agilent, Santa Clara, CA, USA) and the concentrations of all RNA solutions were determined using a Qubit 2.0 fluorometer (Life Technologies/Thermo Fisher Scientific, MA, USA). The RNA samples were used to produce cDNA libraries using the Small RNASeq Library Prep kit (Lexogen GmbH, Wien, Austria) according to the user manual. Input RNA was primarily ligated to a 30 adapter then, after removing excess 30 adapter by column purification, it was ligated to 50 adapter and the excess was removed. In the second step the RNA, flanked by 50 and 30 adapters, was converted into cDNA during the PCR amplification step through the adjunction of multiplexing indices. These indices are used to distinguish the single samples after the pooling phase. The library product was cleaned-up and concentrated with gel-based purification protocol. This step removes linker-linker artefacts (120 bp) and long library fragments (> 200 bp). The presence of linker-linker artefacts in the library may reduce the power of amplification and the consequent sequencing results. A size selection using a 6% Tris–Glycine-SDS Precast Polyacrylamide Gel (Life Technologies/Thermo Fisher Scientific, MA, USA) was used to achieve this goal according to the Library prep instruction manual and using a Gel extraction module compatible with PAGE gel purification (Lexogen GmbH, Wien, Austria). The sequencing step was performed with NGS technologies using Illumina Novaseq 6000 SP kit (100 cycles) produced by Illumina (Illumina, Cambridge, UK) [16].

### Computational analysis of deep sequencing data

A data analysis was performed using the pipeline already established at the Bioinformatics and Statistics Core Facility at TIGEM [16]. Briefly, the reads were trimmed to remove adapter sequences and low-quality ends and reads mapping to contaminating sequences (e.g., ribosomal RNA, phIX control) were filtered out. Reads were aligned and assigned to Human ENSEMBLE transcripts and genes (hg38 reference) by using RSEM version 1.2.25 with standard parameters. The threshold for statistical significance chosen was False Discovery Rate (FDR) < 0.05. The Gene set enrichment analysis (GSEA) was then performed restricting the output to the collection of “hallmark”, “Biocarta”, “Wikipathway”, “Reactome” and “KEGG” gene sets part of the Molecular Signatures Database (MSigDB v7.0). The threshold for statistical significance chosen in the GSEA was False Discovery Rate (FDR) < 0.25. The expression of differentially induced/suppressed genes (FDR < 0.05) was validated by RT-PCR. RNA-seq and smallRNA-seq data are included within this paper and its Supplementary Information files. Data have been deposited in NCBIs Gene Expression Omnibus (GEO) and a Provisional GEO accession number has been requested. The accession number will be provided from the corresponding author upon request.

### Integrated analysis of miRNA and mRNA

Correlation analysis of mir181c/d and predicted target genes based on the mRNA:miRNA interaction network was performed by considering the expression profile in BTC patients. The network was constructed by considering the negative statistically significantly correlation (Pearson’s correlation) between miRNA and mRNA expression, which included mir181c/d and 352 predicted target mRNAs. To create the interaction network, we converted the read counts into counts-per-million (CPM) values for both mRNA and miRNA data. Cytoscape tool was used to build network of interaction (Cytoscape 3.8.2 version, https://cytoscape.org). Targetscan 7.2 (http://www.targetscan.org/vert_72/) and miRDB were used to identify predicted targets. To explore the correlation of candidate target genes with miRNAs, Cytoscape software (Cytoscape 3.8.2 version, https://cytoscape.org) was employed to construct and analyzed the miRNA-hub gene network.

### Prediction of miRNA-mRNA regulatory network

The list of targeted genes included in this analysis was derived from the functional gene set analysis performed with the up-regulated genes identified by the meta-analysis. Briefly, we extracted the list of genes associated with the functional gene ontology terms and biological pathways predicted by ClueGO. The list of conserved miRNAs predicted to target these genes was identified by Targetscan 7.2 (http://www.targetscan.org/vert_72/). To reconstruct the miRNA-mRNA regulatory network, the expression intensity of each miRNA and their respective targets was extracted from the normalized expression (RNAseq and small-RNAseq) data of the BTC cohort. Pairwise correlation was computed between miRNA and their targets. MiRNAs that have a negative correlation (R equal or less than − 0.5) with at least one target were selected for the network reconstruction. Network visualization was performed in Cytoscape software (Cytoscape 3.8.2 version, https://cytoscape.org)  considering miRNA and mRNA as source and target nodes respectively.

### Reporter assay

To prepare the reporter constructs, the 3′UTR of target genes containing the putative miR-181c/d oligonucleotides containing SIRT1 3’UTR binding sites were designed and purchased to Eurofins (Milan, Italy). The oligonucleotides were cloned at the downstream of the luciferase gene in the pGL3-luc vector (Promega, Madison, WI, USA) as schematically depicted in Fig. 5 [16]. For generation of the mutant reporters, oligonucleotides containing mutated binding sites were used. Oligonucleotides sequences were reported below. H69 cells were co-transfected with reporter plasmid (250 ng), pRL-CMV-Renilla plasmid (25 ng) and 40 nM mimic-miRNA and/or negative control mimics (Dharmacon, Lafayette, CO, USA) in 96-well plates using Lipofectamine 2000 (Invitrogen, California, CA, USA) according to the manufacturer’s instructions [16]. After 48 h of transfection, luciferase activity was measured using a Dual Luciferase Reporter Assay system (Promega, Madison, WI, USA) according to the manufacturer’s instruction. Firefly luciferase activity was normalized to Renilla luciferase activity.

SIRT1 3UTR_s: 5’pCTAGACCACCAGCATTAGGAACTTTAGCATGTCAAAATGAATGTTTACTTGTGAACTCGATAGAGCAAGGAAACC T-3′.

SIRT1 3UTR_anti: 5’p CTAGAGGTTTCCTTGCTCTATCGAGTTCACAAGTAAACATTCATTTTGACATGCTAAAGTTCCTAATGCTGGTGG T-3′

SIRT1 3UTR-MUT_s:

5’pCTAGACCACCAGCATTAGGAACTTTAGCATGTCAATATTTTTTTTTACTTGTGAACTCGATAGAGCAAGGAAACC T-3′.

SIRT1 3UTR-MUT_anti: 5’p CTAGAGGTTTCCTTGCTCTATCGAGTTCACAAGTAAAAAAAAATTTTGACATGCTAAAGTTCCTAATGCTGGTGG T-3′

### Statistical analysis

Statistical tests including Student’s t test and one-way ANOVA with Bonferroni’s multiple comparison test were performed with Prism GraphPad (GraphPad Software 8.0, La Jolla, CA, USA). Data are presented as mean +/− SD. Results were considered statistically significant if *p* < 0.05. The calculation of the GI50 values were performed with Prism GraphPad Prism (GraphPad Software 8.0, La Jolla California, USA) and followed a nonlinear regression model applied to the sigmoidal dose–response curves of the cell viability data. The values were log transformed before fitting the model. 4.13. For survival data, Kaplan–Meier curves were plotted and compared using a logrank test. All tests were two-sided. To evaluate the effect of mir-181b and -181c on survivall  in BTC patients, we performed a log-rank test between high and low-mi181a/b risk groups. We stratified patients into high- and low-risk subtypes with median risk score. Kaplan–Meier curve was used to compare the OS (overall survival) and PFS (Progression free survival) between the high- and low risk groups. All analysis was performed with R software (version 4.0.2) using survival R package. A p-value < 0.05 was considered statistically significant.

## Results

### Expression profile of miR-181c/d in BTC patients and association with clinical outcome.

**Fig. 1:**
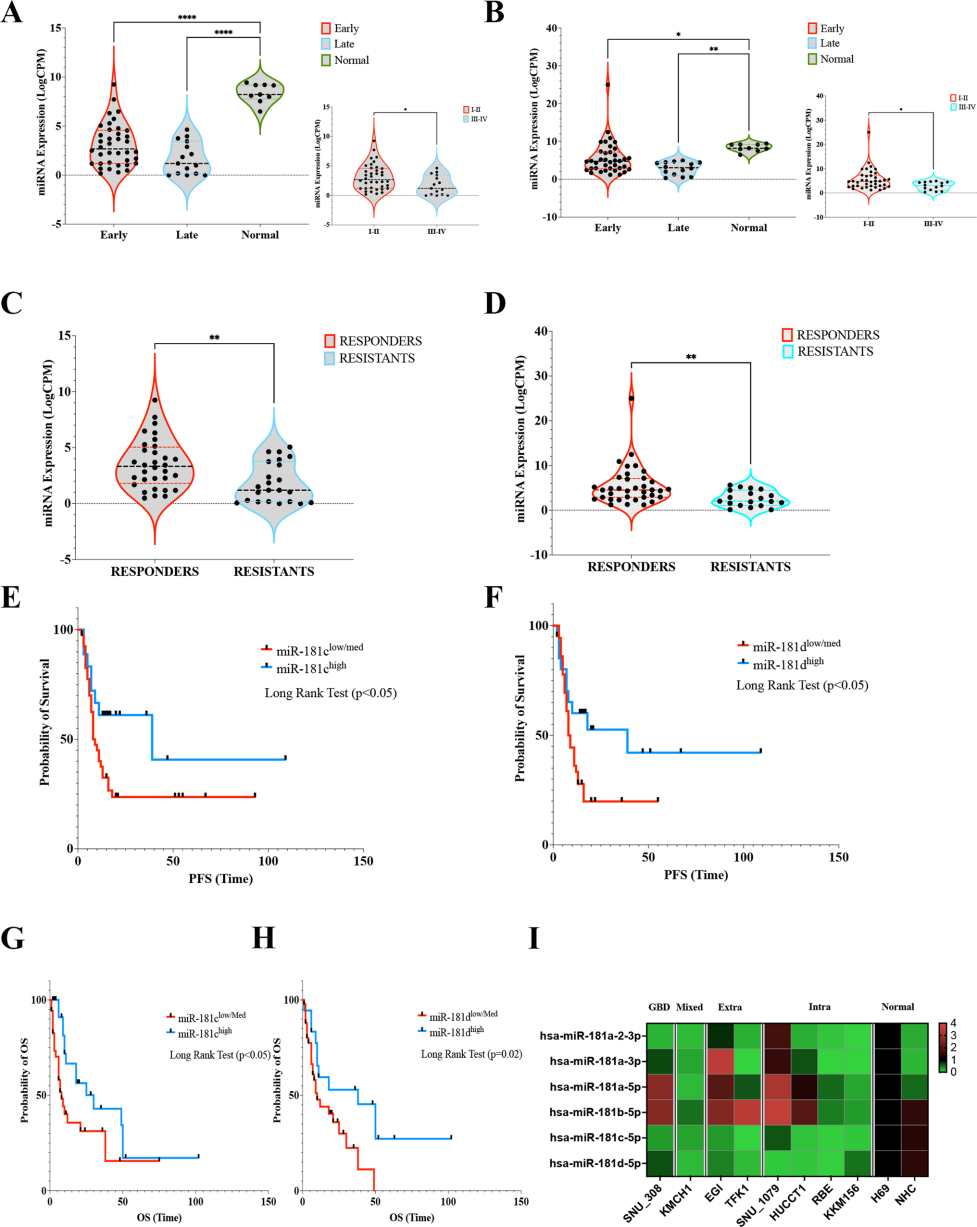
Expression profile of miR-181c/d in BTC patients and association with clinical outcome. **A-B** Violin plots showing the expression (CPM log) of miR-181c (**A**) and miR-181d (**B**) in 62 BTC patients grouped in Early (TNM I/II), Late (TNM III/IV) on the basis of TNM stage; the expression in normal samples (Normal) reported; the upper/lower quartile and the median were indicated. **C-D** Violin plots showing the expression (CPM log) of miR-181c (**C**) and miR-181d (**D**) in 62 BTC patients grouped in sensitive (CR, PR) and resistant (PD) to chemotherapy according to best response to the treatment (RECIST criteria). Data show violin plots indicating the upper/lower quartile and the median. **E–F** Kaplan–Meier estimating Progression-free survival (PFS) of patients with high and low/medium miR-181c (**E**) and -181d (**F**) expression. Patients were stratified by medians into miR-181c-and -181d low/medium and miR-181c-and -181d groups. Pairwise log-rank test was used to analyse the survival; *p* < 0.05. **G-H K**aplan–Meier estimating Overal survival (OS) of patients with high and low/medium miR-181c (**G**) and -181d (**H**) expression. Patients were stratified by medians into miR-181c-and -181d low/medium and miR-181c-and -181d groups. Pairwise log-rank test was used to analyse the survival; *p* < 0.05. **I** Heatmap depicting the expression (Log-CPM) of miRNA-181 family members in BTC and Cholangiocytes cell lines. Cell lines were grouped on the basis of their site of origin (GBD: Gallbladder; Mixed: mied intra- and extra-haepatic; Extra: extra-haepatic; Intra: Intra-haepatic; Normal: cholangiocytes). Each row represents a gene, each column represents a cell line; percentages for all cell types are presented; white fields represent low percentages; blue fields represent high percentage scores. Statistical analyses were conducted by unpaired t test or one-way ANOVA with Tukey’s post hoc analysis. *p < 0.01; **p < 0.001; ***p < 0.0001

The expression profile of miR-181c and -181d was assessed by small-RNA sequencing (small-RNAseq). In particular, we isolated total RNA from formalin-fixed paraffin-embedded (FFPE) tissue of a clinically-annotated cohort of 62 BTC patients (Table S1). The expression of miR-181c and -181d was significantly higher in normal samples with respect to tumor counterparts (Fig. 1A, B). Moreover, early stages (I-II) of BTC shown an increased expression of both microRNAs compared to advanced stages (III-IVA) (Fig. 1A, B). For both miRNAs, no significant association were detected between their expression and specific BTC subtypes (Fig. S1A).

All patients developed unresectable recurrent disease and were treated with first-line platinum/gemcitabine chemotherapy. According to Response Evaluation Criteria in Solid Tumors (RECIST, version 1.1) [23], 26 patients experienced progressive disease (PD) or stable disease (SD) as best response, while 36 achieved an objective response that was a partial response (PR) in all cases. Patients experiencing PR constituted the group of “responders”, whereas patients with PD or SD as best response were included in the group of “resistants”. Overall Survival (OS) was significantly prolonged for responders *vs.* resistants group, as revealed by Long-Rank Survival Test (Fig. S1B). Median OS was 61 months (95% CI 2.88–12.93) for responders, compared with 10 months (95% CI 0.077–0.34) for non-responders (HR 0.13; 95% CI 0.06–0.27; *p* < 0.0001). The expression profile of miR-181c and -181d revealed that both miRNAs were significantly downregulated in resistant patients (*P* = 0.005) (Fig. 1C, D). Consistently, patients benefitting from systemic treatment, displayed an increased tissue expression of both miRNAs (Fig. 1C, D). An inverse association was also observed between miR-181c and -181d and plasma levels of CA19-9, the most frequently used biomarker for diagnostic and treatment predictive purposes in BTC patients [24], suggesting that both miRNAs could serve as predictive prognostic factor in patients with BTC treated with chemotherapy (Fig. S1C).

In order to test their prognostic impact, we assessed whether miR-181c and -181d were correlated with patient survival using the Kaplan–Meier plots. Samples were split in two groups: those with high or low miRNA expression levels (median split). As shown in Fig. 1, patients with high levels of expression of miR-181c (n = 44) and -181d (n = 41) were found to have a better progression-free survival (PFS) than those expressing low levels of miR-181c (n = 21) and -181d (n = 24) (Fig. 1E, F; *P* < 0.05). The median PFS for patients with low levels of miR-181c and -181d was 9 and 8.5 months, respectively, while for miR-181b/c high-expressing patients was 39 months (hazard ratio-HR, 0.23; 95% CI, 0.10 to 0.50 comparing the low miR-181c-group versus the high-group; HR, 0.21; 95% CI, 0.1054 to 0.44 comparing the low miR-181d-group versus the high-group) (Fig. 1E, F). Noticeable improvements were found in the high miR-181c/d-group where high expression levels of both miRNAs were associated also with an increased overall survival (OS) (*P* < 0.05; Fig. 1G, H).

Small-RNAseq analysis allowed us to obtain the expression profile of all miRNAs belonging to miR-181 family in a panel of 8 BTC (SNU_308; KMCH1; EGI; TFK1; SNU_1079; HUCCT1; RBE; KKM156) and 2 cholangiocyte (H69; NHC) cell lines. Unlike other miRNA family members, miR-181c and -181d were found significatively down-regulated in cancer cells compared to normal cholangiocytes, suggesting that both miRNAs may function as tumor-suppressors (Fig. 1I; S1D). When comparing the expression of miR-181c and -181d between cell lines originating from different BTC subtypes, no specific expression was found, letting us to hypothesize that miR-181c/d expression is not specific to a particular anatomical subsite of BTC (Fig. 1I).

In line with the hypothesis that miR-181c/d may act as tumor suppressors in BTC, the above-described findings demonstrated that expression levels of miR-181c/d may be associated to a positive outcome in BTC patients treated with chemotherapy, thus strongly suggesting that miR-181s may be used as predictive biomarkers in the treatment of BTC.

### MiR-181c/d replacement affects the growth of BTC cells

**Fig. 2:**
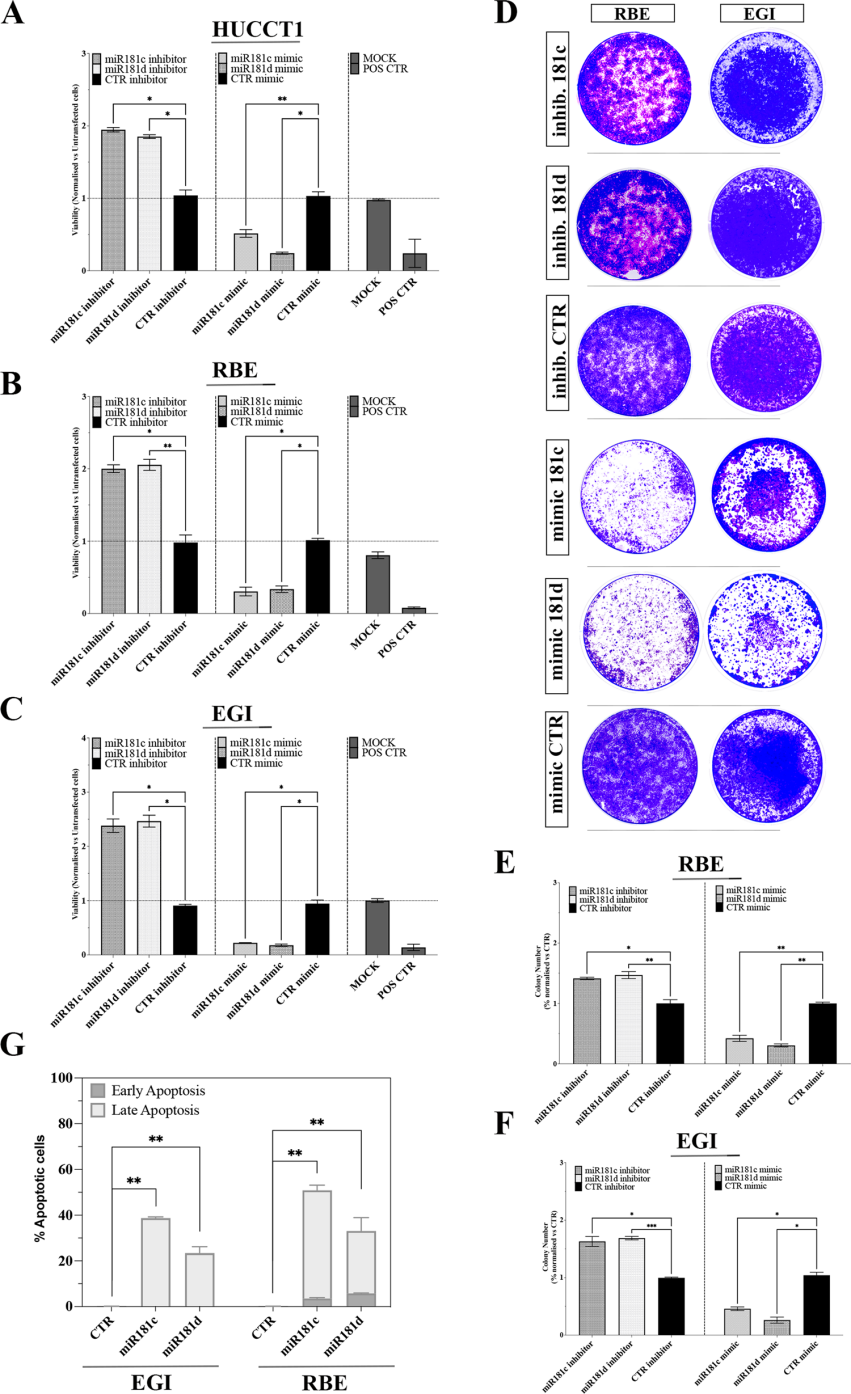
MiR-181c/d replacement Affects the Growth of BTC Cells. **A-B-C** HUCCT01, RBE and EGI cells were transiently transfected with 40 nM of the indicated mimic or inhibitor or the appropriate controls (Control: CTR; Mock transfection control: MOCK; Positive control: POS CTR) and assayed for proliferation by the MTT assay. Data are expressed in terms of percentage of cell viability as compared to untransfected cells. Each value represents the arithmetic mean of three independent experiments performed with triplicate samples. *p < 0.01; **p < 0.001. **D-E–F** Colony formation assays were performed to determine the colony formation ability of RBE (**D-E**) and EGI (**D; F**) BTC cells transfected with miR-181c and -181d mimics or inhibitor or the appropriate controls. Representative images and quantification of visible colonies have been presented. The colony number in the CTR group was set as 100%. All the experiments were performed in triplicate, and the relative colony formation rates are shown as the mean +/− SD. **G** Flow cytometry analysis of apoptosis in EGI and RBE cells transfected with miR-181c and -181d mimics and the appropriate controls. for 48 h. The degree of apoptosis has been determined by annexin V/PI staining. Bars showing average percentages of early/late apoptotic positive cells are presented. The data are means +/− SD of three independent experiments. Statistical analyses were conducted by unpaired t test or one-way ANOVA with Tukey’s post hoc analysis. *p < 0.01; **p < 0.001; ***p < 0.0001

We next investigated whether replacement or inhibition of miR-181c and -181d could affect the proliferation of two intra-hepatic (HUCCT1, RBE) and one (EGI) extra-hepatic BTC cell lines. As illustrated in Fig. 2A–C, transfection of oligonucleotides mimicking the mature forms of miR-181c/d (mimic) significantly suppressed the viability of BTC cells compared to controls (Fig. 2A–C). Using a similar approach, we also examined the effect of inhibition of endogenous miR-181c and -181d activity on cell viability, demonstrating that the inhibition of the two miRNAs enhanced cell proliferation with respect to the controls (Fig. 2A–C).

The effect of miR-181c/d overexpression on the colony formation abilities of BTC cells was also evaluated. Results clearly demonstrated that forced expression of the two miRNAs significantly reduced the number of colonies respect to the controls (Fig. 2D–F). Moreover, the transfection of oligonucleotides inhibiting the expression of miR-181c/d increased the colony counts in both cell type, thus indicating that miR-181c/d depletion may trigger tumor growth in vitro (Fig. 2D–F). The transfection efficiency of miR-181c/d in BTC cells was evaluated by qRT-PCR (quantitative Real-Time-PCR; Fig. S1E). These results suggest that miR-181c and -181d act as effective tumor suppressor microRNAs, since their replacement in BTC cell lines clearly inhibits cell growth.

We further investigated if miR-181c/d may suppress tumor growth in vitro by promoting apoptosis. In order to analyze the effect of miR-181c and -181d on apoptosis we used the Annexin-V/PI assay. Results showed that forced expression of miR-181c/d significantly increased the percentage of apoptotic cells compared to controls (Fig. 2G, S1F). The findings described above suggest that miR-181c/d may act as tumor suppressor in BTC cell lines by inhibiting tumor proliferation and activating apoptosis.

**Fig. 3:**
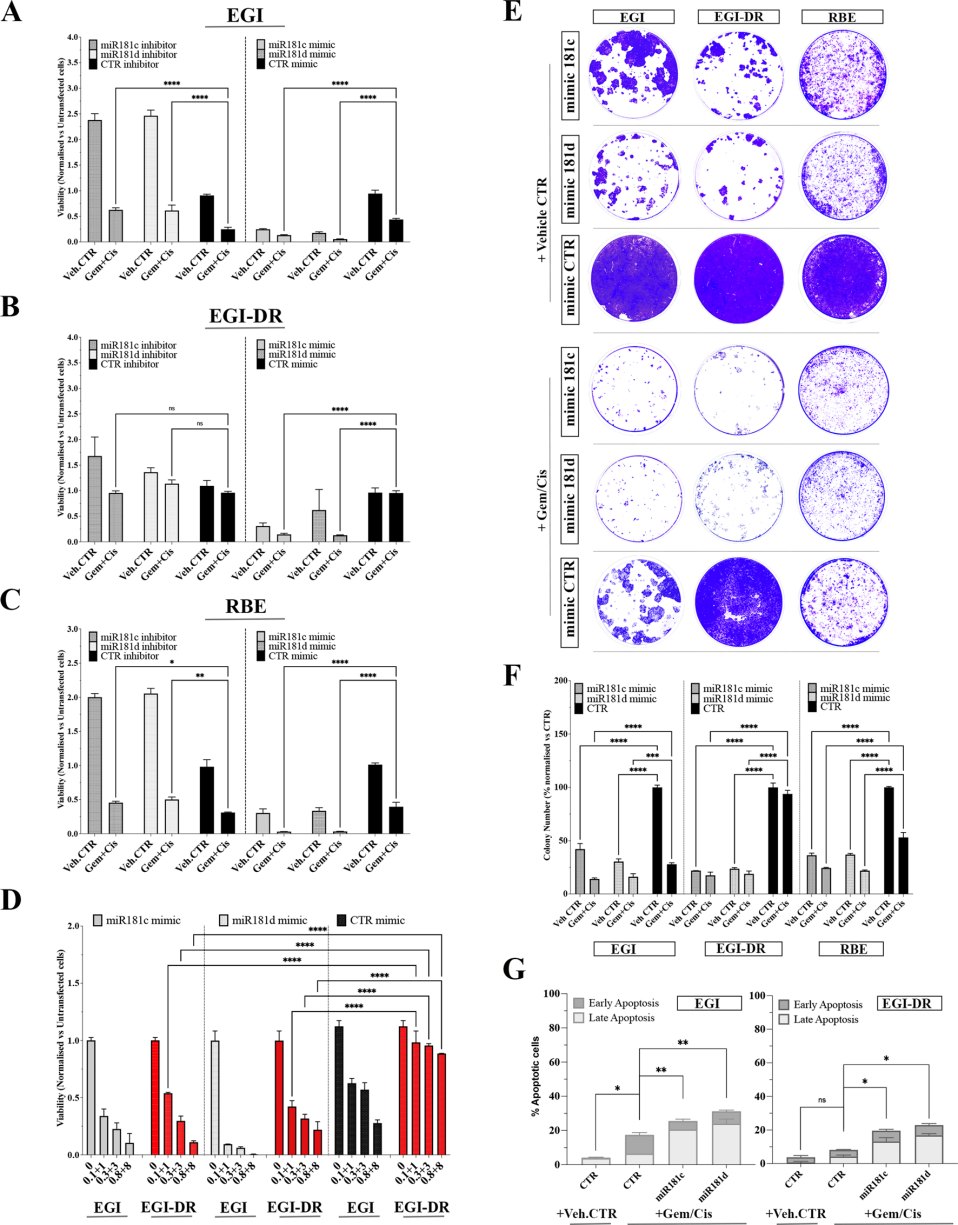
MiR-181c/d Replacement Sensitizes Resistant Cells to chemotherapy. **A-B-C** EGI **(A)**, EGI-DR **(B)**, and RBE **(C)** cells transiently transfected with 40 nM of the indicated mimic or inhibitor or the appropriate controls were treated with combination of Gemcitabine (Gem; 0.1 µM) and Cisplatin (Cis; 1 µM) and assayed for proliferation by the MTT assay. Data are expressed in percentage of cell viability as compared to Vehicle control (Veh.CTR) treated cells. Each value represents the arithmetic mean of three independent experiments performed with triplicate samples. **D** Effect of miR-181c and -181d mimic transfection on cell proliferation in EGI and EGI-DR cells treated with increasing doses of Gem/Cis. Data are represented as mean +/− SD, n = 3. **E–F** Colony formation assays were performed to determine the colony formation ability of EGI, EGI-DR and RBE cells transfected with miR-181c and -181d mimics or the appropriate controls and treated with Gem/Cis (0.1 + 1 µM) ore Veh.CTR. Representative images and quantification of visible colonies have been presented. The colony number in the Vehicle CTR-treated group was set as 100%. All the experiments were performed in triplicate, and the relative colony formation rates are shown as the mean +/− SD. **G** Effect of forced expression of miRNA-181c and -181d on Apoptosis in EGI, and EGI-DR cells treated with Gem/Cis (0.1 + 1 µM) ore Veh.CTR. The degree of apoptosis has been determined by annexin V/PI staining. Bars showing average percentages of early/late apoptotic positive cells are presented. The data are means +/− SD of three independent experiments. Statistical analyses were conducted by unpaired t test or one-way ANOVA with Tukey’s post hoc analysis. *p < 0.01; **p < 0.001; ****p < 0.00001; ns, not significative

**Fig. 4:**
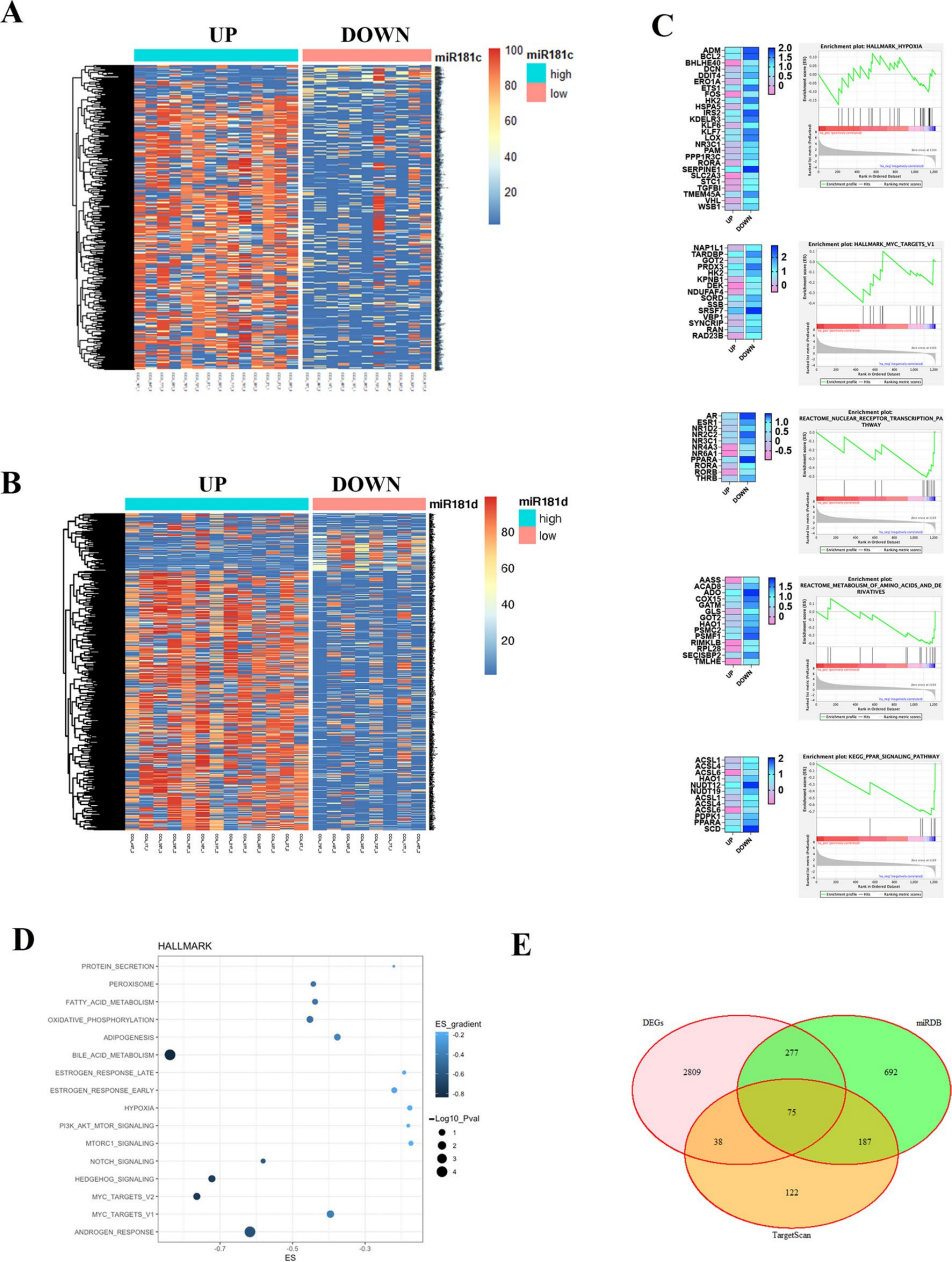
MiR-181c/d Modulate the Response to Chemotherapy by Targeting Key Signaling Networks in BTC*.* **A-B** Heatmap demonstrating log relative expression level of differentially expressed genes between the groups of patients in which miRNAs were UP-regulated and orange the DOWN-regulated. Cell colors (blue-white-orange-red gradient) correspond the binary logarithm of the ratio of the expression level in a current sample to the average level across all the samples (per each gene). Cluster analysis grouped samples and differentially expressed genes (DEGs) according to similarity in expression. DEGs are in rows and samples in columns. The DEGs clustering tree is indicated on the left. To create the heatmap we converted the read counts into log2-counts-per-million (logCPM) values. **C** Representative GSEA plots of Hypoxia, MYC targets, Nuclear Receptor Transcription, Metabolism of Aminoacids and Derivatives, and PPAR signaling pathway gene signatures. Only the gene sets that showed the normalized enrichment score (NES > 2.0) and false discovery rate (FDR) q value < 5% were reported. Representative Heatmaps of differentially expressed genes constituting the leading-edge subsets within the gene sets shown. Each row represents a gene, and each column represents the Median Fold Change (Log) of miRNA-181c/d UP-regulated patients vs DOWN-regulated. **D** Gene set enrichment analysis (GSEA) with compiled modules from Hallmarks in MSigDB. Dot plots of GSEA results illustrated Hallmarks gene network associated with miRNA-181c/d expression in BTC patients. The figures show the significant top 15 positively and the top 15 negatively enriched GO terms, based on co-expressed genes. Dot color indicates the Enrichment Score (ES). Dot size indicates the *p* value (− Log10 *p*value). **E** A Venn diagrams showing the candidate the DEGs and miRNA181c/d targets identified using the prediction algorithms TargetScan, and miRDB

### MiR-181c/d replacement sensitizes resistant cells to chemotherapy

**Fig. 5:**
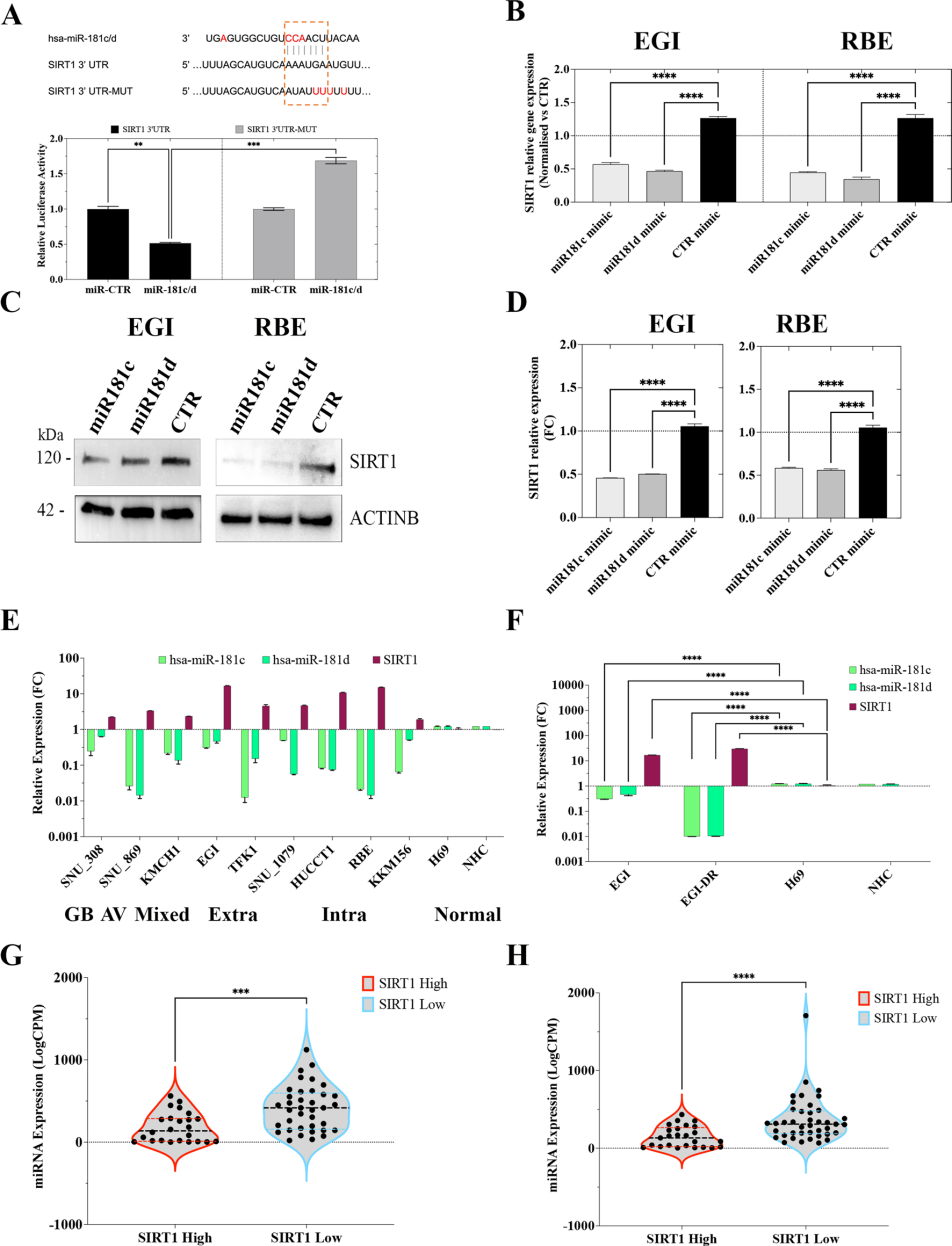
MiR-181c/d negatively regulate SIRT1 expression in BTC. **A** Schematic representation of SIRT1 3′UTR containing predicted miR-181c and -181d binding sites. Red-labelled nucleotide indicate the differences between the sequences of miR-181c and -181d. The dual luciferase reporter assay was performed with H69 cells as described in Materials and Methods. Briefly, H69 cells were transfected with miR-181c (miR-181c/d) or miR mimic negative control (miR-CTR) together with the plasmid encoding for the Firefly luciferase (FFL) carrying part of the SIRT1 3′-UTR in its wild type form (SIRT1 3UTR) or SIRT1 3ʹ-UTR with the mutagenized form of the miR-181c/d binding site (SIRT1 3UTR-MUT). FFL activities were internally normalized to Renilla luciferase activities yielding relative light units (RLU). Histograms show the mean +/− SE from 3 to 6 independent experiments upon normalization to the miR mimic control. ** *p* < 0.01; *** *p* < 0.001 in two-way-ANOVA test. **B** SIRT1 mRNA expression analysis using Quantitative Real-Time polymerase chain reaction (qRT-PCR) in EGI and RBE cells transfected with miR-181c and -181d mimics or relative controls. Shown are the mean +/− SE from 3 independent experiments upon normalization to the miR mimic control. ****p < 0.0001 in un-paired t-test. **C-D** Western blot analysis for SIRT1 and ACTINB of protein lysates obtained from EGI and RBE cells transfected with miR-181c and -181d mimics or relative controls. **D** SIRT1protein expression (histograms) was estimated by integrated optical density in western blots (Fig. 5C) after normalization to the ACTINB, (n = 3). Data are expressed as fold change respect to controls; error bars represent the SD. **E** QRT-PCR analysed miR-181c, miR-181d and SIRT1 mRNA expression in BTC cells. Cell lines were grouped on the basis of their site of origin (GBD: Gallbladder; Mixed: mied intra- and extra-haepatic; Extra: extra-haepatic; Intra: Intra-haepatic; Normal: cholangiocytes). Values are expressed in Log scale. Data represented are mean +/− SD of three independent experiments. **F** QRT-PCR analysed miR-181c, miR-181d and SIRT1 mRNA expression in EGI, EGI-DR and two cholangiocyte cell lines. Values are expressed in Log scale. Data represented are mean +/− SD of three independent experiments. **G-H** Violin plots showing the expression (Log-CPM) of miR-181c (G) and miR-181d (H) in BTC patients grouped on the basis of the SIRT1 expression in 62 BTC patients (median split); the upper/lower quartile and the median were indicated; *p* < 0.05. Statistical analyses were conducted by unpaired t test (Mann–Whitney test). ***p < 0.0001; ****p < 0.00001

With the aim to investigate the contribution of miR-181c and -181d in modulating the response to chemotherapy, we used sensitive and resistant BTC cell lines. We benefited from the use of gemcitabine/cisplatin (gem/cis)-resistant cells (EGI-DR) generated by continuous selective culture and RBE cells which presented a GI_50_ higher than the other cell lines. In particular, the analysis of miR-181c and -181d expression revealed that EGI-DR and RBE cell lines showed significantly reduced expression of both miRNAs respect to other BTC cell lines (Fig. 5E, F). Moreover, we observed that the GI_50_ of gem/cis (combined treatment) correlated with both miRNA expression levels, but in an inversely proportional manner (Fig. S1G, H), suggesting that the miR-181c and -181d may have a crucial role in regulating the response to chemotherapy.

We thus designed in vitro experiments to evaluate the functional role of the miR-181c/d in modulating drug-response. Having demonstrated that miR-181c/d were down-regulated in gem/cis-resistant cells, we investigated whether re-expression of both microRNAs could also sensitize BTC cells to gem/cis and thus attenuate resistance. Resistant (RBE; EGI-DR) and parental (EGI) cell lines were transfected with miR-181c/d mimic and inhibitor and further treated with gem/cis. The overexpression of miR-181c/d significantly inhibited BTC cell growth and enhanced the sensitivity of both sensitive (EGI) and resistant (RBE; EGI-DR) cells to gem/cis (Fig. 3A–C). On the contrary, the depletion of miR-181c and -181d reduced the sensitivity of BTC cells with respect to treated controls (Fig. 3A–C). Notably, the viability of resistant cells (RBE; EGI-DR) transfected with mimic miR-181c/d and treated with gem/cis was reduced by more than 50% with respect to treated controls (Fig. 3A–C).

At the same time, we tested the effect of miR-181c/d silencing in sensitive and resistant cells. Results showed in Fig. 3A, B revealed that depletion of miRNA-181c/d in EGI and RBE cells enhanced the proliferation even in the presence of gem/cis, thus demonstrating a promoting effect on resistance. On the other hand, in resistant EGI-DR cells, no significant effect on proliferation was encountered after treatment with gem/cis compared to controls (Fig. 3B). The lack of a significant effect after inducing forced down-regulation could be explained as the levels of miR-181c/d expression in resistant cells is very low (Fig. 5F).

We next performed a dose–response proliferation assay by overexpressing miR-181c and -181d in EGI- and EGI-DR cells and further treating with increasing concentrations of gem/cis (Fig. 3D). As expected, in EGI cells, gem/cis induced a dose-dependent inhibition of proliferation, while in EGI-DR the treatment did not produce any significative results at all drug concentrations tested (Fig. 3D). Interesting, the replacement of both miR-181c and -181d marked increased the sensitivity to gem/cis treatment (Fig. 3D).

To confirm these data, we carried out in vitro colony formation assays (Fig. 3E, F). The assay was performed by transfecting EGI, EGI-DR and RBE cells with miR-181c, -181d and control mimics, followed by treatment with vehicle control and gem/cis. Representative images and their quantification confirmed that miR-181c/d were able to strongly affect colony formation in EGI, EGI-DR and RBE cells (Fig. 3E, F). Moreover, forced expression of miR-181c or -181d followed by gem/cis treatment significantly decreased the number of colonies in both sensitive (EGI) and resistant (EGI-DR) BTC cell lines (Fig. 3E, F), thus demonstrating that replacement of miR-181c/d may sensitize BTC cells to gem/cis treatment. The transfection efficiency of miR-181c/d in BTC cells was evaluated by qRT-PCR (Fig. S1E).

We further investigated whether the modulation of sensitivity/resistance against gem/cis operated by miR-181c/d may be correlated to alteration of the apoptosis. Flow cytometric AnnexinV assay was performed in EGI and EGI-DR cell lines in which miR-181c/d were overexpressed by transient transfections and treated with gem/cis. As showed in Fig. 3G, in both sensitive and resistant cells, the forced expression of miR-181c and -181d markedly increased the percentage of apoptotic cells with respect to untransfected cells (Fig. 3G; S1I). Notably, the replacement of miR-181c/d induced apoptosis in gem/cis resistant cells thus sensitizing to the treatment (Fig. 3G; S1I).

Taken together, the results described above demonstrated that replacement of 181c/d may reverse the resistance of BTC cells to gem/cis by inducing apoptosis, further supporting the functional contribution of both miRNAs to regulating mechanisms of resistance.

### MiR-181c/d modulate the response to chemotherapy by targeting key signaling networks in BTC

To understand the molecular mechanisms adopted by miR-181c and -181d to regulate the sensitivity and resistance to chemotherapy in BTC, we performed a transcriptome profiling of 62 BTC patient-derived tissue samples by RNA-seq. An integrative pipeline correlating small-RNAseq and total RNAseq data was also established. The analysis has been conducted according to the levels of expression of both miRNAs, applying a cut-off median to select samples with low and high miR-181c/-181d expression (Fig. 4A, B; S2A-B). To select differentially expressed genes (DEGs), we ranked genes by the log2 fold change with adjusted p values ≤ 0.05. Differential expression analysis between miR-181c and -181d high and low expressing group identified respectively 973 and 949 differentially expressed genes (DEG) (Fig. 4A, B; Fig. S2A, B; Table S2; FDR < 0.05). The pattern of gene expression showed two different unsupervised clusters indicating a divergent expression pattern between miR-181c/d high and low expressing tumor groups (Fig. S2A, B). Since miRNA-181c and -181d belong to the same miRNA cluster and their sequence has a large similarity, we started to capture the impact of both miRNAs (miR-181c/d) on BTC patients rather than focus exclusively on a single one.

To gain a better understanding of the functional differences between miR-181c/d high and low expressing tumor groups and elucidate potentially unique signatures, we applied gene set enrichment (GSEA) analysis of the DEG from the molecular signature database (MSigDB) using hallmark (Fig. 4D), wikipathways, reactome and KEGG pathways (Fig. S2H–J). GSEA revealed key cancer-related pathways regulated by both miR-181c and -181d (Fig. 4C, D; Fig. S2C–E; S2H-J; Table S3). Several negative enriched hallmark gene sets were identified in the miR-181c/d high-expressing group, including the oncogenic networks MYC, Hedgehog, NOTCH, PI3K/AKT/MTOR, WNT, Hypoxia (Fig. 4C, D; S2C; Table S4). Other representative Metabolism-related processes were also found to be down-regulated such as Oxidative Phosphorylation, Fatty Acid and Bile Acid Metabolism, Glycolysis, Mitochondrial Biogenesis gene sets (Fig. 4C, D; S2D; S2H-J; Table S4). Interestingly, signaling pathways, commonly associated with drug-resistance, including ABC proteins transports and disorders were negatively enriched (Fig. S2E; Table S3). Importantly, there were significant inverse correlations between miR-181c/d expression and cancer-related oncogenic pathways as mTOR and Hedgehog (Fig. 4C, D; S2C-E; S2H-J; Table S3).

DEGs were further screened using two common miRNA target prediction engines (TargetScan, miRDB), thus identifying 75 DE target genes potentially regulated by miR-181c/d expression (Fig. 4E; Table S4). The resulting DEG targets were further filtered to the genes that are differentially expressed on RNA-seq analysis (Fig. 4E; Table S3). We thus established a correlation value between the upregulated miRNAs and their downregulated target genes from the miRNA and relative mRNA profiling (Fig. S2F, G). Among negative-regulated genes, the analysis showed several experimentally validated target genes of miR-181 family members, such as LATS1 [25], TRIM2 [26], ACVR2A [27], NOTCH4 [28], RAP1B [29], MAP3K3 [30], PRKCD [31], ADCY1 [32], ZNF562 [33], YWHAG [34], KRAS [35], CARD11 [36], PEAK1 [37], CREB1 [38], SIRT1 [26, 39], belonging to key cancer-related signaling pathways (Fig. S2F, G; Table S4). Belonging to genes regulating drug-resistance in cancer, the presence of the SIRT1 represents a predicted target of particular interest (Fig. S2F, G; Table S4). In fact, emerging evidence demonstrated that SIRT1 acts as a resistance-driver gene by epigenetically regulating metabolic and mitochondrial gene signatures in cancer, including BTC [40–43].

### MiR-181c/d negatively regulate SIRT1 expression in BTC

As stated before, it has been shown that SIRT1 (Silent information regulator 2 homolog 1) acts as an oncogene in cancer regulating mitochondrial biogenesis, cellular redox and associated metabolic pathways, and several oncogenic signaling [41, 44]. We thus decided to experimentally validate SIRT1 as direct target of both miR-181c and -181d. Potential targets of miR-181c and -181d were predicted using three bioinformatic databases (TargetScan, miRanda and PicTar). SIRT1 was chosen as a target gene of both miR-181c and -181d, based on putative target sequences of SIRT1 localized in the 3′UTR (Fig. 5A).

To verify whether SIRT1 is a direct target of miR-181c/d, a human SIRT1 3′UTR fragment containing the binding site of the two miRNAs (Fig. 5A) or the same construct carrying a mutant binding site (negative control) were cloned into the pGL3 vector and the miR-181c mimic or miR-CTRL were co-transfected into H69 cells and cultured for 48 h. Then luciferase activities were measured by a luminescent assay. As expected overexpression of miR-181c suppressed the luciferase activity of wild-type SIRT1 3′UTR but the activity of the mutant-type SIRT1 3′UTR was not changed (Fig. 5A), indicating SIRT1 as direct miR-181c/d target.

Consistent with the hypothesis that SIRT1 may represent a mRNA target for both miR-181c and -181d, SIRT1 mRNA and protein expression was found to be significantly down-regulated by the enforced expression of miR-181c and -181d in 2 BTC cell lines compared to controls (Fig. 5B–D).

Expression analysis performed by qRT-PCR, shown a negative correlation between miR-181c/d and SIRT1 expression in all BTC cell lines, independently from their site of origin (Fig. 5E; S1J). The inverse correlation between miR-181c/d and SIRT1 was not observed in normal cholangiocytes (Fig. 5E; S1J), while it is significantly more pronounced in BTC resistant cells (Fig. 5F), suggesting that the molecular interplay between miR-181c/d and SIRT1 may modulate drug resistance in BTC. Moreover, the integrative analysis of small- and total-RNAseq data from our BTC patient cohort, evidenced a negative correlation between miR-181c/d and SIRT1 expression (Fig. 5G, H), raising evidence of a potential clinical importance of miR-181/SIRT1 axis.

Taken together these results indicated that miR-181c/d binds directly to SIRT1 and inhibits its expression in BTC both in patients and cell lines, suggesting that their molecular interplay may be relevant in oncogenic mechanism driving cholangiocarcinogenesis.

### MiR-181c/d regulate chemotherapy response by targeting SIRT1

**Fig. 6:**
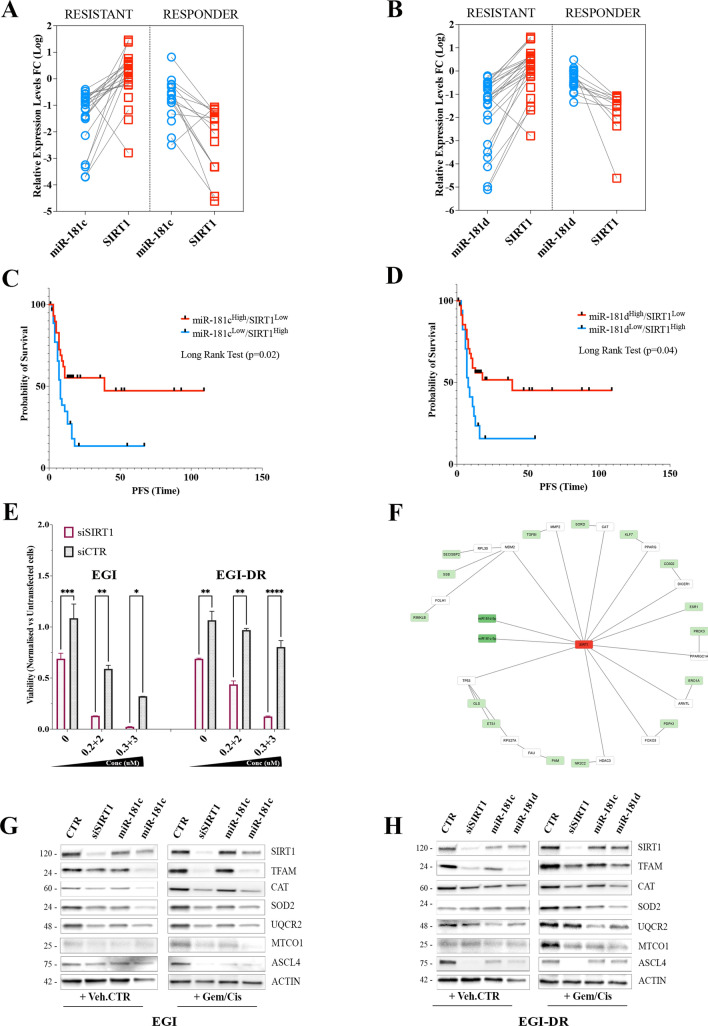
MiR-181c/d Regulate Chemotherapy Response by Targeting SIRT1. **A-B** Resistant and responder BTCs display miR-181c/d-SIRT1 axis deregulation. Lower miR-181c **(A)** and -181d **(B)** and higher SIRT1 expression levels were found in patients resistant to chemotherapy, compared to tumors from responders group. *p* < 0.001 by Mann–Whitney U test. **C-D** Kaplan–Meier estimates PFS of patients with high and low miR-181c/d-SIRT1 expression ratio. Patients were stratified by medians into miR-181c/d^High^/SIRT1^Low^ and miR-181c/d^Low^/SIRT1^High^ groups. Pairwise log-rank test was used to analyse the survival. **E** Effect of SIRT1 silencing on cell proliferation in EGI and EGI-DR cells treated with increasing doses of Gem/Cis. Data are represented as mean +/− SD, n = 3. Cell lines were transfected with either SiRNA control (siCTR), or SIRT1 specific siRNAs (SiSIRT1). **F** MiRNA-181c- and -181d-SIRT1 mRNA network and negatively regulated DEG genes in BTC patients visualized by Cytoscape. MiRNAs are in dark green boxes; SIRT1 in red box in the middle of the network, while and SIRT1-interacting target genes in the surrounding white and green boxes. The green boxes identify the DE mRNAs negatively correlated with miRNA-181c and -181d expression **G-H** Western blot analysis for SIRT1, TFAM, CAT, SOD2, UQCR2, MTCO1, ASCL4 and ACTINB of protein lysates obtained from EGI (**G**) and EGI-DR (**H**) cells transfected with miR-181c, -181d mimics, siSIRT1 or relative controls and treated with Gem/Cis or Veh.CTR

Next, we explored the clinical significance of SIRT1-targeting by miR-181c/d in BTC using the patient cohort described in Table S1. A correlation analysis led us to demonstrate an inverse and significant relationship between miR-181c/d levels and SIRT1 mRNA levels in BTC patients (Fig. 5G, H; 6A, B). In particular, BTC patients resistant to chemotherapy reported an increased SIRT1 expression correlated with a concomitant down-regulation of both miR-181c and -181d (Fig. 6A, B), thus highlighting that reduced expression of miR181c/d correlated with drug resistance. The negative correlation between both miRNAs and SIRT1 was highly significant in the “responders” subtype (Fig. 6A, B). In the “resistant’ group with low miR-181c/d expression, SIRT1 mRNA expression was significantly increased, thus validating the inverse correlation.

To further dissect the impact of reciprocity between miR-181c/d and SIRT1 expression on patient response, we stratified the patients into miR-181c/d^high^-SIRT1^low^ and miR-181c/d^low^-SIRT1^high^ (median split). Progression-free survival-based analysis of the two groups demonstrated that patients belonging to the miR-181c/d^high^-SIRT1^low^ group showed a significantly improved PFS, extending beyond 36 months for 55% of this subgroup (Fig. 6C, D). On the contrary, PFS was significantly shorter in the miR-181c/d^low^-SIRT1^high^ group (median 8 months vs. 39 months; *P* < 0.05 by long-rank test; Fig. 6C, D).

The analysis of miR-181c/d expression revealed that BTC-resistant cell lines showed significantly decreased levels of miR-181c and -181d (Fig. 5F). Moreover, we observed that the GI_50_ of gem/cis (combined treatment) correlated with both microRNA expression levels, but in an inversely proportional manner (Fig. S1G, H), suggesting that the miR-181c/d-SIRT1 axis may have a crucial role in regulating the response to gem/cis treatment.

We thus designed in vitro experiments to evaluate the functional role of the miR-181c/d-SIRT1 axis in regulating chemotherapy resistance. We previously demonstrated that miR-181c and -181d replacement sensitizes resistant BTC cells to gem/cis (Fig. 3). Similar experiments demonstrated that SIRT1 depletion from sensitive (EGI) and resistant cells (EGI-DR), via RNAi, significantly impacted on the resistance to gem/cis treatment, with respect to control-treated cells (Fig. 6E). As expected, gem/cis treatment resulted in dose-dependent cytotoxicity in the parental line but had no effect on EGI-DR cells. In contrast, gem/cis retained full potency and anti-proliferative effect in both lines, after SIRT1 siRNA-mediated depletion (Fig. 6E).

To gain insights into the mechanism used by miR-181c/d-SIRT1 axis to modulate the sensitivity and resistance to chemotherapy, an integrative miRNA-mRNA network analysis incorporating protein–protein interaction (PPI) was performed using Cytoscape (Table S5; Fig. 6F; *p* < 0.05). A perturbed miRNA-181c/d-SIRT1 regulatory networks was built-up selecting a gene list according to the following criteria: i) correlation to miRNA-181c/d based on target prediction, and target validation; ii) miRNA/mRNA expression data from the RNA seq-based dataset.

The SIRT1-centered circuitry resulted in a regulatory network which included 29 nodes representing SIRT1-interacting genes, 31 edges representing interactions between genes, the two miRNAs and SIRT1 (Fig. 6F). The proposed method identified a miR-181c/d-SIRT1 network where the primary hub gene SIRT1 was connected directly with ESR1 and indirectly with ten hub-structured genes (Fig. 6F; Table S5). For a selective visualization of the most important miRNA-181c/d-SIRT1-modulated genes, we highlighted the interactions of relevant SIRT1-modulated pathways (Fig. 6; Table S5). Interesting, our integrative analysis demonstrated that the miRNA-181c/d-SIRT1 network had a negative regulatory effect on several signaling previous shown to modulate SIRT1-induced chemoresistance, including FOXO [45], and TP53 [46] pathways (Fig. 6F; Table S5). Functional enrichment analysis of the miR-181c/d-SIRT1 network revealed that most of the genes are involved in various important biological processes, which trigger SIRT1-mediated tumorigenesis and drug resistance [47] (Hipoxia; MYC-targets; MTORC signaling; ABC family; PPAR signaling; TP53 signaling) (Fig. 6F; Table S5). The analysis also identified regulatory networks associated to cellular metabolism and drug efflux, mitochondrial homeostasis, oxidative stress, PGC-1α, gluconeogenesis and oxidation of fatty acids metabolic pathway, known to be targets of SIRT1-dependent modulation (Fig. 6F; Table S5) [47, 48]. Taken together, these results indicate that the deregulation of the metabolic signaling pathway mediates the functional effects of miR-181c/d-SIRT1 axis on BTC drug sensitivity and resistance.

As the transcriptomic data revealed that miR-181c/d-SIRT1 may modulate multiple mitochondrial- and metabolism-related pathways, particularly those associated with OXPHOS (Mitochondrial Oxidative Phosphorylation System) and cellular resistance to oxidative stress, we next evaluated the functional effect of both miR-181c/d overexpression and SIRT1 knocking down (KD) on mitochondrial homeostasis, fatty acid metabolism and OXPHOS profile in sensitive and resistant BTC cells. The Mitochondrial transcriptional factor A (TFAM) has been shown to act as a key player in the regulation of mitochondrial metabolism [49], drug resistance [] and oncogenic activation in several tumors [49, 50]. Several studies have also shown that SIRT1 may induce the expression of TFAM and other metabolism-related players [51, 52]. TFAM, Catalase and SOD2 were chosen as representative markers of mitochondrial activity and oxidative stress response, and their expression was analyzed first by western blotting (Fig. 6G, H). The analysis demonstrated that forced overexpression of miR-181c and -181d and siRNA-mediated depletion of SIRT1 markedly reduced the expression levels of TFAM, Catalase and SOD2 in both EGI and EGI-DR cells (Fig. 6G, H). The decreased expression of TFAM, Catalase and SOD2 was also detected in EGI and EGI-DR cells treated with gem/cis, raising evidence that miR-181c/d-SIRT1 axis may modulate the sensitivity to gem/cis by negatively regulating the SIRT1-mediated metabolic reprogramming of BTC. We further analyzed the expression profile of UQCR2 and MTCO1, two proteins belonging to OXPHOS. The analysis revealed reduced levels of both proteins in EGI and EGI-DR cells overexpressing miR181c/ mimics or silenced for SIRT1 (Fig. 6G, H). MiR-181c and -181d forced overexpression and SIRT1 KD markedly reduced UQCR2 and MTCO1 levels also in sensitive and resistant EGI cells treated with gem/cis (Fig. 6G, H). Moreover, the protein levels of a key regulator of lipid metabolism, ACSL4 were also decreased by miR-181c and -181d overexpression and SIRT1 KD in sensitive and resistant BTC cells treated and not with gem/cis, thus highlighting the negative modulation of miR-181c/d-SIRT1 axis on lipid metabolism.

Based on the proposed integrative network, and functional validation, the interplay between miR-181c/d and SIRT1 had a negative regulatory effect on several important metabolic-related pathways modulating drug resistance in BTC. Our results strongly support a central role for the miR-181c/d-SIRT1 axis in modulating key components of tumor growth with target therapy resistance both in vitro and BTC clinical sample. Taken together, our findings demonstrated that the negative correlation between the expression of miR-181c/d and SIRT1 is associated with chemotherapy resistance in BTC. We also exploited the clinical significance of the miR-181c/d-SIRT1 axis raising evidence that the miR-181c/d^low^/SIRT1^high^ status may predict a poor response in patients under treatment with chemotherapy.

## Discussion

In this study, using both patient-derived material and preclinical models, we demonstrated for the first time that miR-181c and -181d, act as tumour suppressive miRNAs in BTC. Indeed, we showed that their downregulation is associated with advanced tumour stage and poor clinical outcome. Mechanistically, the forced overexpression of miR-181c and -181d reduced tumour proliferation and counteracted chemoresistance in in vitro BTC models by targeting histone deacetylase *SIRT1.* Our findings support the translational potential of these miRNAs as predictive biomarkers and therapeutic tools in BTC.

The miR-181 family consists of four highly conserved members: miR-181a, miR-181b, miR-181c, and miR-181d [53]. The sequence homology and difference among miR-181a-d and their gene loci on different chromosomes were elucidated by Indrieri et al. [53]. The miR-181 family members are evolutionarily conserved among the vertebrate lineage with high homology implicating their functional redundancy [53]. Notably, miR-181s are aberrantly expressed in tumor tissues and exhibit oncogenic or tumor-suppressive properties in a cancer-specific manner. The involvement of several members miRNA-181 family in cancer and drug-resistance has been demonstrated in a widely range of tumors [53, 54]. Human miR-181c and -181d are transcribed in clusters at genomic locus on chromosomes 19 and are involved in the regulation of multiple cellular functions [38]. MiR-181c and -181d are emerging as onco-suppressors in multiple cancer types, including non-small cell lung cancer (NSCLC) [55], breast cancer [56], hepatocellular carcinoma [57], bladder cancer [58], esophageal squamous cell carcinoma [59, 60], brain cancers [61]. However, miR-181d have also been shown to exhibit oncogenic properties in colorectal cancer (CRC) [62], lung adenocarcinoma [63], glioma [64]. Several other microRNAs presented dual-opposite oncogenic or suppressive functions which may be attributed to organ-specific actions or different cellular contexts of tumors.

Numerous studies evidenced that miRNAs regulate several mechanisms driving drug-resistance [65]. Moreover, therapeutic strategies aiming to overexpress oncosuppressor-miRNAs or inhibit onco-miRs may represent efficient approaches to overcome drug-resistance or increasing the efficacy of therapeutic regimens. The advent of precision medicine has paved the way for the introduction of miRNAs as biomarkers to predict therapeutic responses and cancer patient survival [6, 65, 66]. In recent years, many studies have proposed miRNAs as potential biomarkers as diagnostic, prognostic, and predictive tools in BTC [12, 67, 68]. Respect to other class of molecules, miRNA possesses several advantages: i) miRNA-isolation and detection in liquid biopsies (from blood, urine, and other bodily fluids) can be easily performed; ii) miRNAs possess a high specificity and sensitivity for the tissue or cell type of origin; iii) miRNA detection present high time- and cost-effectivenes in comparison to other available biomarkers; iv) the detection can be multi-plexed: a multi-miRNA profile (miRNA signature) provides a non-invasive method for the diagnosis and prediction of disease progression and treatment efficacy [12].

Based on their biological relevance, members of the miR-181 family have been investigated as prognostic and predictive biomarkers in CRC [69], oral [70], esophageal [71], endometrial [72], and NSCLC [54], breast [73], ovarian [74] and pancreatic [75] cancers. Recently, our group shown that miR-181a and -181b may represent new biomarkers for therapeutic intervention in refractory melanomas [16].

We found here that the downregulation of both miR-181c and -181d is a commonly occurring feature within the tumour area of BTC and that was correlated with a significantly shorter survival in our clinically-annotated patient cohort.

Furthermore, the increased expression of both miRNA levels was associated with a higher likelihood of achieving a response to standard chemotherapy treatment in the advanced-disease setting. We can speculate that the improved sensitivity to cytotoxics driven by miR-181c and -181d is a key factor for the better survival outcome experienced by BTC with upregulation of these miRNAs.

Through functional experiments we indeed showed that the forced expression of both miRNAs increased sensitivity to gem/cis in resistant BTC cell models, markedly suppressing cell viability and colony formation and inducing cell death. Although preliminary and in need of validation, this finding strongly suggests potential use of miR-181c and -181d as therapeutic tools. Potential applications of multidrug resistance-related miRNAs, including miR-181a/b have been already investigated in cancer [16, 53, 76–80].

Here, we report the first evidence that SIRT1 is regulated by miR-181c/d in BTC and to a greater extent in cancer. Our experiments, suggest that miR-181c and -181d directly interact with the SIRT1 3′UTR to regulate SIRT1 expression. In support of this hypothesis, the expression level of miR-181c/d was inversely correlated with the SIRT1 transcript level in BTC specimens. Furthermore, the suppressive effect of miR-181c/d on SIRT1 expression sensitizes BTC cells to gem/cis treatment. Finally, high expression of miR-181c/d (and, thus, lower SIRT1) was associated with improved PFS in BTC patients.

SIRT1 is a nicotinamide adenosine dinucleotide (NAD/NADH)-dependent histone deacetylase (HDAC) that has been linked to control of longevity, gene silencing, cell-cycle progression, apoptosis, and energy homeostasis [43]. Its expression has been shown to be altered in cancer cells, and it targets both histone and non-histone proteins for deacetylation and thereby alters metabolic programs. Interestingly, many of the metabolic pathways that are influenced by SIRT1 are also altered in tumor development. Not only does SIRT1 have the potential to regulate oncogenic factors, it also orchestrates many aspects of metabolism and lipid regulation and recent reports are beginning to connect these areas. SIRT1 influences pathways that provide an alternative means of deriving energy (such as fatty acid oxidation and gluconeogenesis) when a cell encounters nutritive stress and can therefore lead to altered lipid metabolism in various pathophysiological contexts [43]. Recent studies have revealed the significance of SIRT1 as oncogene in BTC indicating that overexpression of SIRT1 is correlated with cell proliferation and progression of BTC [42]. The same authors proposed the use of SIRT1 inhibitors as effective therapeutic approach against BTC [42, 81]. Importantly, increasing evidence suggests that SIRT1 is a major player in cancer drug resistance [41]. Our results provide evidence of a negative correlation between miR-181c/d expression and their target SIRT1 in patients with a positive outcome and responding to targeted therapies, thus strongly suggesting that miR-181c/d exert their tumor-suppressive action by targeting SIRT1 and thus silencing its pro-tumorigenic functions. Numerous SIRT1 small molecule inhibitors have been developed or are under development [82]. However, they shown encouraging anti-cancer effect against cancers, but a limited specificity and potency [82]. Our results proposed miR-181b and -miR181c as new generation of SIRT1 inhibitors with promising effects on overcoming cancer drug resistance and improve therapeutic outcomes of cancer treatment. Interesting, other member of miR-181 family were reported to mitochondrial-related metabolism, and antioxidant response [16, 53, 83].

Finally, our analysis of the functional role of miR-181c/d also investigated their regulatory network, thus identifying key signaling pathway regulating BTC progression and resistance. In particular, a network of genes, involved in transcriptional regulation of metabolic processes and cell death, has been found to be negatively enriched in BTC with high expression levels of miR-181c/d. Metabolic reprogramming is a hallmark of cancer and allows tumour cells to meet the increased energy demands required for rapid proliferation, invasion, and metastasis [84]. Targeting altered tumour metabolism is an emerging therapeutic strategy for cancer treatment, including BTC. We reported that miR-181c and -181d might regulate several key-metabolic related fundamental functions, notably associated to a tumor suppressive role and to a positive response to therapeutic agents in BTC^81^. Our results strongly suggest the potential use of miR-181c/d in targeting SIRT1-mediated metabolism in BTC.

In summary, we demonstrated here for the first time that miRNA-181c and -181d play a tumour suppressive role in BTC by targeting the histone deacetylase *SIRT1*. As such, they are downregulated in tumour areas and in higher disease stages. Interestingly, we showed that their expression confers a better prognosis and more favourable response to standard chemotherapy both in the patient cohort and preclinical models. Collectively, these data support the transformative clinical application of miRNA-181c and -181d as novel predictive biomarkers and therapeutic tools in BTC.

## Supplementary Information

Below is the link to the electronic supplementary material.

Supplementary file1 (TIF 23198 KB)

Supplementary file2 (TIF 23198 KB)

Supplementary file3 (TIF 29 KB)

Supplementary file4 (TIF 376 KB)

Supplementary file5 (PDF 114 KB)

Supplementary file6 (PDF 91 KB)

Supplementary file7 (PDF 68 KB)

Supplementary file8 (DOCX 27 KB)

## Data Availability

RNA-seq and smallRNA-seq data are included within this paper and its Supplementary Information files. Data have been deposited in NCBIs Gene Expression Omnibus (GEO) and a Provisional GEO accession number has been requested. The accession number will be provided from the corresponding author upon request.
