# Supplementary figures and images for: A MiR181/Sirtuin1 regulatory circuit modulates drug response in biliary cancers
Source: Clin Exp Med. 2024 Apr 10;24(1):74. doi: 10.1007/s10238-024-01332-0 (PMC11006774; doi:10.1007/s10238-024-01332-0)

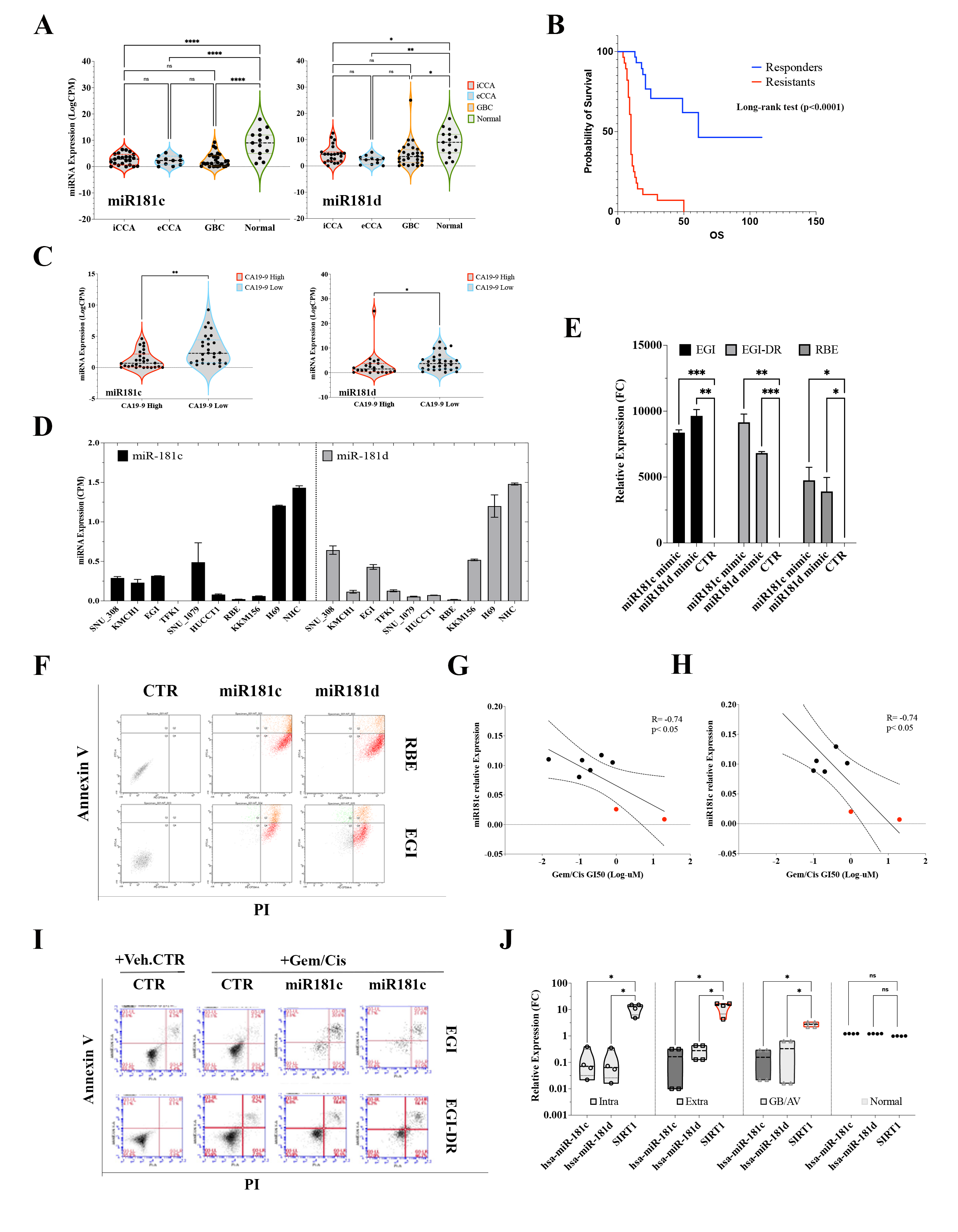

Supplement: Supplementary file 1 — Supplementary file1 (TIF 23198 KB) [file 10238_2024_1332_MOESM1_ESM.tif]

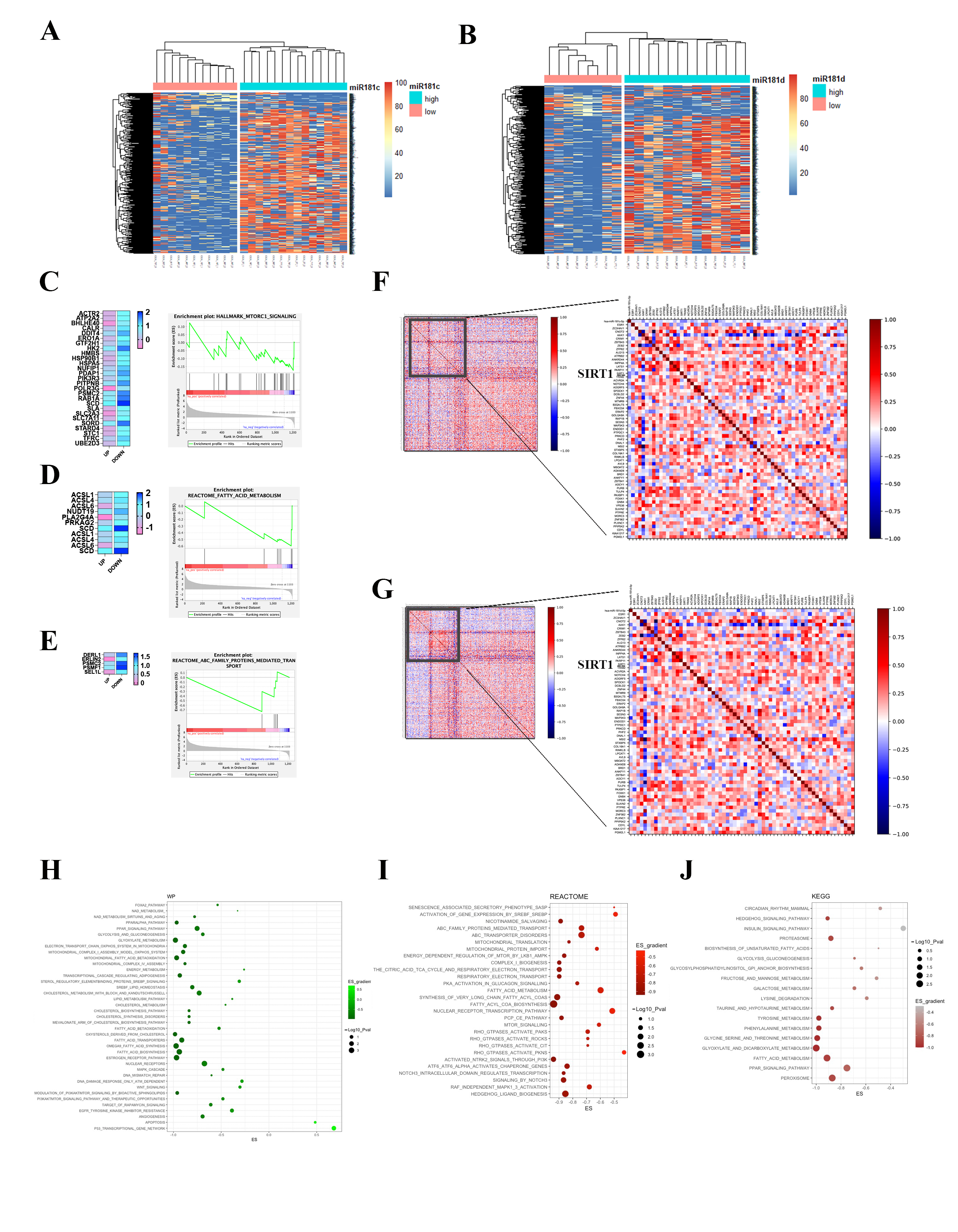

Supplement: Supplementary file 2 — Supplementary file2 (TIF 23198 KB) [file 10238_2024_1332_MOESM2_ESM.tif]
