# Supplementary material for: A MiR181/Sirtuin1 regulatory circuit modulates drug response in biliary cancers
Source: Clin Exp Med. 2024 Apr 10;24(1):74. doi: 10.1007/s10238-024-01332-0 (PMC11006774; doi:10.1007/s10238-024-01332-0)
Supplement: Supplementary file 3 — Supplementary file3 (TIF 29 KB) [file 10238_2024_1332_MOESM3_ESM.pdf]

Table S1. Baseline demographic and clinicopathologic characteristics (n=62).

Characteristic	N (%)
Age, years (median, range)	64 (36-88)
Gender	
Female	38 (61%)
Male	24 (39%)
ECOG PS	
0-1	45 (73%)
≥2	17 (27%)
Subtype	
iCCA	24 (39%)
eCCA	11 (18%)
GBC	27 (43%)
CA19-9	
<37U/mL	42%
>37U/mL	58%
Stage	
I-II	41 (66%)
III-IV	21 (34%)
pT	
1	8 (10%)
2	19 (37%)
3	23 (42%)
4	8 (3%)
x	4 (8%)
pN	
0	28 (45%)
1	34 (55%)
Resection margins	
R0	42 (68%)
R1	20 (32%)
Adjuvant chemotherapy	
Yes	36 (58%)
No	26 (42%)
