# Supplementary material for: A MiR181/Sirtuin1 regulatory circuit modulates drug response in biliary cancers
Source: Clin Exp Med. 2024 Apr 10;24(1):74. doi: 10.1007/s10238-024-01332-0 (PMC11006774; doi:10.1007/s10238-024-01332-0)
Supplement: Supplementary file 4 — Supplementary file4 (TIF 376 KB) [file 10238_2024_1332_MOESM4_ESM.pdf]

Table S2. Differentially expressed genes p-value filtered (<0.05) in BTC patients grouped as miR-181c and miR-181d UP and DOWN expression

Gene Name	EnsemblGeneID	logFC	pval	regulation	miRNA
MECR	ENSG00000116353	-1,389044062	0,007689872	down	miR-181d
KDM4C	ENSG00000107077	-2,050686957	0,016832373	down	miR-181d
CYP27C1	ENSG00000186684	-2,24924966	0,008813162	down	miR-181d
FADS1	ENSG00000149485	-0,779272244	0,004074681	down	miR-181d
C3orf35	ENSG00000198590	-1,941192875	0,019466435	down	miR-181d
PDPN	ENSG00000162493	-1,515187778	0,014189391	down	miR-181d
NCKAP5L	ENSG00000167566	-1,046800862	0,032053621	down	miR-181d
CENPF	ENSG00000117724	-0,888954408	0,021763383	down	miR-181d
CCL23	ENSG00000274736	-1,434010996	0,0197831	down	miR-181d
SCN2A	ENSG00000136531	-2,825688748	0,013625289	down	miR-181d
NR4A3	ENSG00000119508	-2,147319532	0,020675382	down	miR-181d
CCDC181	ENSG00000117477	-2,381211214	0,010571581	down	miR-181d
AC109630.1	ENSG00000259269	-2,657565157	0,015909849	down	miR-181d
PSCA	ENSG00000167653	-3,240834762	0,015400496	down	miR-181d
TRIM29	ENSG00000137699	-2,073470681	0,014595797	down	miR-181d
MEDAG	ENSG00000102802	-2,254110885	0,010276333	down	miR-181d
ADRB1	ENSG00000043591	-2,287767879	0,020487323	down	miR-181d
APOD	ENSG00000189058	-1,535373075	0,025856188	down	miR-181d
GLB1L	ENSG00000163521	-1,594740384	0,044711627	down	miR-181d
ACOT11	ENSG00000162390	-1,467122451	0,013796758	down	miR-181d
BRINP2	ENSG00000198797	-2,388096524	0,018435059	down	miR-181d
SUGCT	ENSG00000175600	-2,21784532	0,006087464	down	miR-181d
SPON1	ENSG00000262655	-1,880624581	0,012841413	down	miR-181d
IFI44L	ENSG00000137959	-1,133179962	0,037689079	down	miR-181d
RCSD1	ENSG00000198771	-1,080254416	0,00314633	down	miR-181d
MRPS9	ENSG00000135972	-1,069120983	0,005457315	down	miR-181d
EGR2	ENSG00000122877	-2,859630634	0,007726673	down	miR-181d
AUTS2	ENSG00000158321	-0,921850769	0,011333404	down	miR-181d
MTRR	ENSG00000124275	-0,820153505	0,008073447	down	miR-181d
NFATC1	ENSG00000131196	-1,141064045	0,004592114	down	miR-181d
HOTAIRM1	ENSG00000233429	-1,115114951	0,010606924	down	miR-181d
CDON	ENSG00000064309	-2,481466756	0,007635436	down	miR-181d
RASA3	ENSG00000185989	-1,088085945	0,03278643	down	miR-181d
TBL1X	ENSG00000101849	-1,130776271	0,033809129	down	miR-181d
ICE2	ENSG00000128915	-0,255096898	0,042379809	down	miR-181d
MMP19	ENSG00000123342	-1,890627099	0,009871606	down	miR-181d
TBCD	ENSG00000141556	-0,664707648	0,042545581	down	miR-181d
GEM	ENSG00000164949	-1,84809479	0,01567272	down	miR-181d
DCTN1	ENSG00000204843	-0,510545326	0,036820498	down	miR-181d
EGR3	ENSG00000179388	-2,3362768	0,012323723	down	miR-181d
CILP	ENSG00000138615	-2,181241258	0,016032756	down	miR-181d
SAMSN1	ENSG00000155307	-1,690083845	0,007561473	down	miR-181d
TMEM168	ENSG00000146802	-1,287805683	0,000582023	down	miR-181d
HAT1	ENSG00000128708	-1,254152864	0,003090928	down	miR-181d
SLC39A8	ENSG00000138821	-2,101271854	0,009424523	down	miR-181d
ITK	ENSG00000113263	-2,329083332	0,009231212	down	miR-181d
GNB4	ENSG00000114450	-0,674806707	0,005648208	down	miR-181d
KLHL6	ENSG00000172578	-0,960186578	0,032734046	down	miR-181d
AC140912.1	ENSG00000261227	-2,969501958	0,017360803	down	miR-181d
GLI3	ENSG00000106571	-1,18242312	0,049151634	down	miR-181d
RYR2	ENSG00000198626	-1,36265315	0,009248721	down	miR-181d
GATA2	ENSG00000179348	-1,761486277	0,02578205	down	miR-181d
SIRT1	ENSG00000096717	-0,750072773	0,016562038	down	miR-181d
HSD17B7	ENSG00000132196	-1,514767081	0,0117744	down	miR-181d
TLR4	ENSG00000136869	-2,388223961	0,003093794	down	miR-181d
ZC3H7A	ENSG00000122299	-0,445426554	0,042396375	down	miR-181d
AL133367.1	ENSG00000260285	-2,28901696	0,018799351	down	miR-181d
LGI2	ENSG00000153012	-2,092297839	0,001639299	down	miR-181d
AC090987.1	ENSG00000253656	-2,326820143	0,03983113	down	miR-181d
MAP3K6	ENSG00000142733	-1,013917353	0,012062501	down	miR-181d
CAMK1D	ENSG00000183049	-1,215026441	0,006503006	down	miR-181d
AC245452.1	ENSG00000224086	-3,23443994	0,019095607	down	miR-181d
TESPA1	ENSG00000135426	-2,967357068	0,016155225	down	miR-181d
PARM1	ENSG00000169116	-2,10373712	0,009225932	down	miR-181d
TFF1	ENSG00000160182	-1,847793058	0,005877855	down	miR-181d
EOMES	ENSG00000163508	-1,891089228	0,007110777	down	miR-181d
TNNI2	ENSG00000130598	-1,759465847	0,00892458	down	miR-181d
AC114760.2	ENSG00000272211	-3,087163854	0,016111096	down	miR-181d
CCR4	ENSG00000183813	-1,853830345	0,007673221	down	miR-181d
AC092376.2	ENSG00000277954	-4,295099966	0,047056709	down	miR-181d

GTDC1	ENSG00000121964	-1,284680985	0,008835978	down	miR-181d
JAKMIP2	ENSG00000176049	-2,970187928	0,009922132	down	miR-181d
MYH7B	ENSG00000078814	-2,779332478	0,040395862	down	miR-181d
RHOH	ENSG00000168421	-2,204708584	0,006618418	down	miR-181d
FLNC	ENSG00000128591	-2,257556498	0,011033331	down	miR-181d
KLK11	ENSG00000167757	-2,691943971	0,002430939	down	miR-181d
TENM3	ENSG00000218336	-3,016990381	0,00013516	down	miR-181d
AL355075.4	ENSG00000259001	-2,718006238	0,002246227	down	miR-181d
ZNF524	ENSG00000171443	-0,991109534	0,014317381	down	miR-181d
RBM43	ENSG00000184898	-0,967907657	0,008459652	down	miR-181d
SPHK1	ENSG00000176170	-1,187877183	0,004106309	down	miR-181d
LRRC15	ENSG00000172061	-2,639618033	0,001810049	down	miR-181d
RETN	ENSG00000104918	-3,916801104	0,014892048	down	miR-181d
LRRC8C	ENSG00000171488	-2,531457891	0,002120031	down	miR-181d
Z97200.1	ENSG00000271811	-1,831707343	0,007827952	down	miR-181d
SLC25A14	ENSG00000102078	-1,167894681	0,01797506	down	miR-181d
RASGEF1A	ENSG00000198915	-2,160773328	0,012166638	down	miR-181d
THEMIS2	ENSG00000130775	-2,452246695	0,000209426	down	miR-181d
TNIP3	ENSG00000050730	-2,176020875	0,010505589	down	miR-181d
AC096677.1	ENSG00000224536	-2,83351367	0,018664723	down	miR-181d
UQCRHL	ENSG00000233954	-2,763274273	0,005636748	down	miR-181d
RGS22	ENSG00000132554	-4,177895144	0,02202901	down	miR-181d
AMBRA1	ENSG00000110497	-1,308830418	0,003286133	down	miR-181d
AC005899.5	ENSG00000266385	-1,91604283	0,023481648	down	miR-181d
DMBT1	ENSG00000187908	-3,125669845	0,021300398	down	miR-181d
ASB11	ENSG00000165192	-4,21351673	0,015648825	down	miR-181d
CACNA1A	ENSG00000141837	-1,202816823	0,022405167	down	miR-181d
PLEKHS1	ENSG00000148735	-2,355030143	0,01178919	down	miR-181d
AC108134.2	ENSG00000261889	-2,75627003	0,043674242	down	miR-181d
NRROS	ENSG00000174004	-2,790646803	0,019885957	down	miR-181d
AL031595.2	ENSG00000280011	-3,347562871	0,024235226	down	miR-181d
AL357033.4	ENSG00000277496	-3,201897291	0,008226136	down	miR-181d
LMOD3	ENSG00000163380	-3,960045781	0,030806188	down	miR-181d
RTP1	ENSG00000175077	-4,108846051	0,02056943	down	miR-181d
ESR2	ENSG00000140009	-4,466381624	0,021883444	down	miR-181d
AC073352.1	ENSG00000272662	-2,778122048	0,022309558	down	miR-181d
CDHR4	ENSG00000187492	-2,691282318	0,001640724	down	miR-181d
TRIM45	ENSG00000134253	2,256918514	0,037268881	up	miR-181d
PHOSPHO2	ENSG00000144362	1,325512444	0,040899065	up	miR-181d
BCL7A	ENSG00000110987	1,998216558	0,024989045	up	miR-181d
PPARGC1A	ENSG00000109819	4,333904643	0,015359103	up	miR-181d
CSTF2T	ENSG00000177613	2,552485886	0,046059703	up	miR-181d
DAB2IP	ENSG00000136848	0,873460826	0,016765332	down	miR-181d
CNTNAP1	ENSG00000108797	2,918963373	0,040826346	up	miR-181d
ZNF575	ENSG00000176472	2,593793794	0,049967707	up	miR-181d
NFYC	ENSG00000066136	2,127480652	0,026501165	up	miR-181d
MRVI1	ENSG00000072952	1,711823428	0,035998168	up	miR-181d
PTER	ENSG00000165983	0,945653901	0,031905006	down	miR-181d
RASAL2	ENSG00000075391	0,706314077	0,043574446	down	miR-181d
RPP25L	ENSG00000164967	1,496678457	0,046723249	up	miR-181d
ALS2CL	ENSG00000178038	1,105007329	0,008550635	up	miR-181d
PARP6	ENSG00000137817	0,53288589	0,044771969	down	miR-181d
BCL3	ENSG00000069399	1,391214832	0,017918747	up	miR-181d
TM2D2	ENSG00000169490	1,64453822	0,010852484	up	miR-181d
SLC6A1	ENSG00000157103	2,986460266	0,001299485	up	miR-181d
ZNF485	ENSG00000198298	1,725657241	0,046924573	up	miR-181d
TPRA1	ENSG00000163870	1,268634367	0,042824011	up	miR-181d
PSMD6	ENSG00000163636	0,892125987	0,035149787	down	miR-181d
AC084125.4	ENSG00000265393	2,639747972	0,029461563	up	miR-181d
YEATS4	ENSG00000127337	1,274753251	0,023237138	up	miR-181d
C18orf21	ENSG00000141428	1,901480232	0,039267415	up	miR-181d
C5orf49	ENSG00000215217	1,6263295	0,041083226	up	miR-181d
CREB3L3	ENSG00000060566	2,737110044	0,029999326	up	miR-181d
BAIAP2-DT	ENSG00000226137	1,795194296	0,008939771	up	miR-181d
LENG9	ENSG00000275183	1,355779492	0,04172525	up	miR-181d
SNRK-AS1	ENSG00000234617	2,20352694	0,043884694	up	miR-181d
TMEM61	ENSG00000143001	1,386326218	0,025494518	up	miR-181d
ARMCX6	ENSG00000198960	1,302503711	0,034722432	up	miR-181d
TAS2R19	ENSG00000212124	1,834261109	0,012054019	up	miR-181d
ANXA9	ENSG00000143412	3,748141129	0,025978918	up	miR-181d
FICD	ENSG00000198855	2,469802274	0,025472641	up	miR-181d
CNNM1	ENSG00000119946	3,47253448	0,041049436	up	miR-181d
PHLPP2	ENSG00000040199	2,165199871	0,013328483	up	miR-181d
ACTL10	ENSG00000182584	2,600615693	0,002655193	up	miR-181d
LINC01637	ENSG00000237476	2,125613999	0,016873379	up	miR-181d
AL353194.1	ENSG00000229539	3,280365202	0,002146844	up	miR-181d
CBWD2	ENSG00000136682	1,433875976	0,042704156	up	miR-181d

FAM84B	ENSG00000168672	2,533708987	0,007746588	up	miR-181d
TBPL1	ENSG00000028839	1,027620614	0,049742833	up	miR-181d
NR2F1-AS1	ENSG00000237187	2,549786074	0,024580861	up	miR-181d
ZNF253	ENSG00000256771	2,428413971	0,014197013	up	miR-181d
EIF5AL1	ENSG00000253626	1,474974397	0,03860612	up	miR-181d
RBM18	ENSG00000119446	1,523189272	0,033205701	up	miR-181d
ZNF304	ENSG00000131845	1,926309309	0,006654879	up	miR-181d
SLC35E2	ENSG00000189339	2,233027399	0,033051019	up	miR-181d
ZNF529	ENSG00000186020	2,090301815	0,037493528	up	miR-181d
RCBTB1	ENSG00000136144	2,766711841	0,005944828	up	miR-181d
PARVB	ENSG00000188677	0,989549438	0,032223496	down	miR-181d
FAM35A	ENSG00000122376	1,551330442	0,036192392	up	miR-181d
UCK2	ENSG00000143179	1,624907181	0,022918276	up	miR-181d
FAM111B	ENSG00000189057	3,021709529	0,007772145	up	miR-181d
IPO4	ENSG00000196497	1,554954592	0,043115835	up	miR-181d
RNASEH1-AS1	ENSG00000234171	1,753310432	0,018785666	up	miR-181d
WNK2	ENSG00000165238	4,105612394	0,026797435	up	miR-181d
C16orf59	ENSG00000162062	1,889906388	0,023257743	up	miR-181d
PLCD1	ENSG00000187091	2,143202979	0,017326578	up	miR-181d
N6AMT1	ENSG00000156239	2,183644895	0,020157028	up	miR-181d
SPRY4	ENSG00000187678	1,426628316	0,048267929	up	miR-181d
GALNT18	ENSG00000110328	2,003935862	0,021198008	up	miR-181d
ASB16-AS1	ENSG00000267080	1,768719182	0,042851195	up	miR-181d
NUP50	ENSG00000093000	1,048377292	0,01346091	up	miR-181d
TUBGCP5	ENSG00000275835	1,663477774	0,014470355	up	miR-181d
C9orf156	ENSG00000136932	1,935399422	0,04573774	up	miR-181d
CWF19L1	ENSG00000095485	1,126204034	0,017575593	up	miR-181d
MFSD6	ENSG00000151690	1,634435504	0,018494837	up	miR-181d
SNX33	ENSG00000173548	1,136337207	0,028784152	up	miR-181d
NRAV	ENSG00000248008	2,012576484	0,021198568	up	miR-181d
ZDHHC23	ENSG00000184307	3,064939277	0,003142248	up	miR-181d
CAP2	ENSG00000112186	1,609475762	0,013841591	up	miR-181d
AL162377.1	ENSG00000231856	2,397931811	0,016869462	up	miR-181d
RBM15	ENSG00000162775	0,931751332	0,037517128	down	miR-181d
ZNF627	ENSG00000198551	1,184228732	0,037029041	up	miR-181d
ATRN	ENSG00000088812	2,587267981	0,000176217	up	miR-181d
ZNF837	ENSG00000152475	1,66221274	0,035187597	up	miR-181d
DNAL4	ENSG00000100246	2,22350429	0,007481258	up	miR-181d
WBSCR16	ENSG00000274523	1,296563964	0,008197354	up	miR-181d
LIG4	ENSG00000174405	2,801250876	0,002536271	up	miR-181d
RHOU	ENSG00000116574	2,900245297	0,00061971	up	miR-181d
PCCA	ENSG00000175198	1,732233115	0,004402983	up	miR-181d
SOCS5	ENSG00000171150	1,769213684	0,017215712	up	miR-181d
PLIN4	ENSG00000167676	2,494636349	0,005978886	up	miR-181d
PCNA	ENSG00000132646	1,230975926	0,034460069	up	miR-181d
UFM1	ENSG00000120686	1,026294648	0,007891384	up	miR-181d
TBC1D8	ENSG00000204634	1,662387691	0,025169241	up	miR-181d
AC027307.2	ENSG00000267317	2,840142581	0,008132519	up	miR-181d
CGGBP1	ENSG00000163320	0,90073069	0,046256761	down	miR-181d
TRMT5	ENSG00000126814	1,592852321	0,046603407	up	miR-181d
RGMB	ENSG00000174136	1,783469403	0,020013106	up	miR-181d
SUPV3L1	ENSG00000156502	1,05387656	0,026830249	up	miR-181d
DBNDD1	ENSG00000003249	3,214967201	0,002269341	up	miR-181d
CRBN	ENSG00000113851	1,497125627	0,002708481	up	miR-181d
SCLY	ENSG00000132330	2,259236551	0,039414737	up	miR-181d
CCNB1IP1	ENSG00000100814	1,599354329	0,040038789	up	miR-181d
PRKAA2	ENSG00000162409	2,660885994	0,005235812	up	miR-181d
SLC38A6	ENSG00000139974	1,017098783	0,010195549	up	miR-181d
IDE	ENSG00000119912	1,169395065	0,019881741	up	miR-181d
SCX	ENSG00000260428	4,242598087	0,037532376	up	miR-181d
CAPN7	ENSG00000131375	0,987408952	0,028520738	down	miR-181d
GALNT11	ENSG00000178234	1,342687039	0,029623204	up	miR-181d
ARHGEF17	ENSG00000110237	0,969998743	0,046257699	down	miR-181d
KLF13	ENSG00000169926	1,92438861	0,003845548	up	miR-181d
NFYC-AS1	ENSG00000272145	1,991190056	0,048916979	up	miR-181d
SMLR1	ENSG00000256162	1,886352012	0,041710519	up	miR-181d
ING3	ENSG00000071243	2,264649772	0,00458029	up	miR-181d
MRI1	ENSG00000037757	1,597831378	0,008282108	up	miR-181d
MRPL10	ENSG00000159111	1,315928811	0,024461747	up	miR-181d
BCO2	ENSG00000197580	2,703443698	0,011182853	up	miR-181d
AKR1C2	ENSG00000151632	1,595387796	0,027120269	up	miR-181d
RFC2	ENSG00000049541	1,328333128	0,013384046	up	miR-181d
BTBD10	ENSG00000148925	1,636832578	0,004522928	up	miR-181d
CHST4	ENSG00000140835	2,96693419	0,029046248	up	miR-181d
EIF4ENIF1	ENSG00000184708	1,197114536	0,041293626	up	miR-181d
IGFBP4	ENSG00000141753	2,046385521	0,04229052	up	miR-181d
POLI	ENSG00000101751	1,684631162	0,031426684	up	miR-181d

COQ5	ENSG00000110871	1,481342554	0,008176525	up	miR-181d
ABL1	ENSG00000097007	0,959675033	0,032900667	down	miR-181d
CRTAP	ENSG00000170275	0,881906087	0,034660739	down	miR-181d
C9orf106	ENSG00000179082	2,03233222	0,041690466	up	miR-181d
MTX2	ENSG00000128654	2,984351614	0,034664021	up	miR-181d
OSGIN2	ENSG00000164823	1,457698325	0,01544277	up	miR-181d
GATB	ENSG00000059691	2,882482667	0,008283652	up	miR-181d
TPRN	ENSG00000176058	2,519318961	0,001153739	up	miR-181d
TMEM184C	ENSG00000164168	1,891098382	0,029858812	up	miR-181d
AKR7A2	ENSG00000053371	1,373450096	0,039444366	up	miR-181d
MCCC2	ENSG00000131844	1,30529524	0,008400549	up	miR-181d
TMEM14A	ENSG00000096092	1,513122025	0,032228696	up	miR-181d
EXOSC8	ENSG00000120699	1,619042147	0,027699971	up	miR-181d
NCKIPSD	ENSG00000213672	2,314768964	0,018772807	up	miR-181d
ZNF407	ENSG00000215421	1,059274976	0,015105226	up	miR-181d
TSPAN6	ENSG00000000003	0,822209504	0,040288251	down	miR-181d
NAB2	ENSG00000166886	1,585154341	0,034284988	up	miR-181d
AASDHPPT	ENSG00000149313	1,126743665	0,023787077	up	miR-181d
MRRF	ENSG00000148187	1,348953443	0,024180443	up	miR-181d
TMTC4	ENSG00000125247	3,415845735	0,008418042	up	miR-181d
PRR7	ENSG00000131188	2,657312844	0,048578704	up	miR-181d
EMC6	ENSG00000127774	1,127573235	0,045135069	up	miR-181d
IPP	ENSG00000197429	1,384257291	0,032658802	up	miR-181d
ZFP30	ENSG00000120784	1,778446805	0,00540303	up	miR-181d
F8	ENSG00000185010	2,387501767	0,028296445	up	miR-181d
AIFM2	ENSG00000042286	1,752021563	0,02298551	up	miR-181d
CYB561D1	ENSG00000174151	1,993808037	0,025046435	up	miR-181d
ANO10	ENSG00000160746	1,18129701	0,027005706	up	miR-181d
MYO1D	ENSG00000176658	1,549796068	0,042157936	up	miR-181d
SETD7	ENSG00000145391	0,950101313	0,044394207	down	miR-181d
FBXO31	ENSG00000103264	1,197081083	0,045679403	up	miR-181d
JAG1	ENSG00000101384	1,096133352	0,003441332	up	miR-181d
TMEM242	ENSG00000215712	2,242381373	0,03535334	up	miR-181d
TDG	ENSG00000139372	1,019359568	0,016908551	up	miR-181d
KIAA1191	ENSG00000122203	1,183339069	0,049792054	up	miR-181d
SH2B3	ENSG00000111252	1,384659088	0,010134914	up	miR-181d
FAM174A	ENSG00000174132	1,766126855	0,031305897	up	miR-181d
SCRN3	ENSG00000144306	1,498518413	0,049484944	up	miR-181d
TGFBRAP1	ENSG00000135966	1,236731451	0,006277957	up	miR-181d
KIAA1586	ENSG00000168116	1,812905366	0,004644219	up	miR-181d
LRRC20	ENSG00000172731	1,420630431	0,045480427	up	miR-181d
ELOVL7	ENSG00000164181	3,482958779	0,010669775	up	miR-181d
SWAP70	ENSG00000133789	1,31464456	0,032680033	up	miR-181d
TTL	ENSG00000114999	0,674696091	0,04951272	down	miR-181d
VPS26A	ENSG00000122958	0,907434722	0,014416156	down	miR-181d
LOXL4	ENSG00000138131	1,847911359	0,041050487	up	miR-181d
MCAT	ENSG00000100294	1,778456163	0,034001091	up	miR-181d
FOXA2	ENSG00000125798	1,373107399	0,021915338	up	miR-181d
SNX10	ENSG00000086300	1,701536024	0,025981961	up	miR-181d
QRICH1	ENSG00000198218	1,348566676	0,022736969	up	miR-181d
NTPCR	ENSG00000135778	1,331346194	0,020149061	up	miR-181d
CHCHD1	ENSG00000172586	1,168980397	0,009702085	up	miR-181d
DDX51	ENSG00000185163	0,922877417	0,029625893	down	miR-181d
ZNF625	ENSG00000257591	2,168498216	0,033822789	up	miR-181d
PYGO1	ENSG00000171016	1,866466557	0,025700721	up	miR-181d
RIT1	ENSG00000143622	1,39023089	0,00656562	up	miR-181d
HIST1H2BD	ENSG00000158373	1,540348594	0,035019008	up	miR-181d
NDUFAF3	ENSG00000178057	1,196523981	0,01711467	up	miR-181d
ALG1	ENSG00000033011	1,857890661	0,011445951	up	miR-181d
ILKAP	ENSG00000132323	1,915705892	0,002509659	up	miR-181d
ANP32A	ENSG00000140350	0,824951039	0,035802598	down	miR-181d
TMCC1	ENSG00000172765	0,983147129	0,002447691	down	miR-181d
GOLGA3	ENSG00000090615	1,022232171	0,006249461	up	miR-181d
MICU2	ENSG00000165487	1,225906236	0,032310031	up	miR-181d
ESD	ENSG00000139684	2,072628605	0,001174564	up	miR-181d
PPP3CB	ENSG00000107758	1,210423688	0,007927191	up	miR-181d
BRWD1	ENSG00000185658	0,800872877	0,020085283	down	miR-181d
CRYZ	ENSG00000116791	0,862330558	0,040584209	down	miR-181d
STIM1	ENSG00000167323	0,91535463	0,024802384	down	miR-181d
CLK4	ENSG00000113240	0,818191413	0,036218653	down	miR-181d
SRBD1	ENSG00000068784	1,178909787	0,032555858	up	miR-181d
RPL3	ENSG00000100316	2,38309156	0,00425697	up	miR-181d
DNAH5	ENSG00000039139	2,29953768	0,009801596	up	miR-181d
TBC1D5	ENSG00000131374	0,616972807	0,034491464	down	miR-181d
ECI1	ENSG00000167969	1,935725098	0,004325295	up	miR-181d
TSFM	ENSG00000123297	1,925442326	0,016128149	up	miR-181d
SIAH1	ENSG00000196470	1,872243864	0,032598802	up	miR-181d

SLC1A7	ENSG00000162383	2,676172678	0,036636111	up	miR-181d
WDR12	ENSG00000138442	1,134918056	0,010084574	up	miR-181d
NSUN4	ENSG00000117481	1,179437838	0,034651908	up	miR-181d
CADM4	ENSG00000105767	3,31349713	0,001608745	up	miR-181d
RBM4	ENSG00000173933	1,490881862	0,017449098	up	miR-181d
GNB2	ENSG00000172354	1,39942658	0,02336718	up	miR-181d
SSSCA1	ENSG00000173465	1,27765195	0,045069265	up	miR-181d
NUDT2	ENSG00000164978	1,726147749	0,006541987	up	miR-181d
UNC5B	ENSG00000107731	1,51770694	0,019984102	up	miR-181d
PIGK	ENSG00000142892	1,186952275	0,048364709	up	miR-181d
AL683813.1	ENSG00000232611	1,539264808	0,033473756	up	miR-181d
HRSP12	ENSG00000132541	2,409581817	0,005616278	up	miR-181d
DFNA5	ENSG00000105928	2,222402752	0,015874173	up	miR-181d
CCDC174	ENSG00000154781	1,223556813	0,002593847	up	miR-181d
THUMPD3	ENSG00000134077	1,758586028	0,004612735	up	miR-181d
NSUN3	ENSG00000178694	2,765748487	0,004730239	up	miR-181d
CHRNB1	ENSG00000170175	2,260877562	0,034819744	up	miR-181d
HHEX	ENSG00000152804	2,119642978	0,028198279	up	miR-181d
HGD	ENSG00000113924	2,611673216	0,003889469	up	miR-181d
CRLS1	ENSG00000088766	1,537587087	0,018135383	up	miR-181d
DECR1	ENSG00000104325	1,133305933	0,028614898	up	miR-181d
COQ3	ENSG00000132423	1,685496553	0,037757682	up	miR-181d
TAF1B	ENSG00000115750	1,43557977	0,019275685	up	miR-181d
GEN1	ENSG00000178295	1,472707942	0,018901609	up	miR-181d
HAGH	ENSG00000063854	1,929770124	0,000833833	up	miR-181d
SDSL	ENSG00000139410	2,127222939	0,001965345	up	miR-181d
WDR91	ENSG00000105875	1,433973466	0,037391596	up	miR-181d
GATS	ENSG00000239521	1,396580827	0,041166459	up	miR-181d
IVD	ENSG00000128928	0,92417583	0,029232363	down	miR-181d
SMARCAD1	ENSG00000163104	1,966855402	0,009447747	up	miR-181d
TM7SF2	ENSG00000149809	1,514060372	0,024079199	up	miR-181d
ELOVL5	ENSG00000012660	1,413259716	0,015107328	up	miR-181d
PAFAH1B1	ENSG00000007168	0,843560438	0,019280934	down	miR-181d
GMPR2	ENSG00000100938	1,312648308	0,016861183	up	miR-181d
PTPRS	ENSG00000105426	1,204875904	0,01509953	up	miR-181d
ECSIT	ENSG00000130159	1,094909467	0,02105716	up	miR-181d
UBAP2L	ENSG00000143569	1,133980208	0,035974703	up	miR-181d
UGDH	ENSG00000109814	1,918298271	0,00065098	up	miR-181d
SEC24A	ENSG00000113615	1,424139042	0,004741589	up	miR-181d
SEC14L2	ENSG00000100003	1,421745704	0,008239402	up	miR-181d
MAN2A2	ENSG00000196547	0,849065334	0,022837356	down	miR-181d
TYW5	ENSG00000162971	1,127034724	0,026404473	up	miR-181d
GLIS3	ENSG00000107249	0,618372746	0,047190816	down	miR-181d
NDUFA7	ENSG00000267855	1,10096073	0,049860864	up	miR-181d
SNRPB2	ENSG00000125870	1,449452527	0,012385516	up	miR-181d
C6orf1	ENSG00000186577	0,867670031	0,040438707	down	miR-181d
STAM	ENSG00000136738	1,730848752	0,002114626	up	miR-181d
STOM	ENSG00000148175	1,547388428	0,013013269	up	miR-181d
WRNIP1	ENSG00000124535	1,886534587	0,027967202	up	miR-181d
STX12	ENSG00000117758	0,775134868	0,044601278	down	miR-181d
KLHL5	ENSG00000109790	0,828270873	0,036801651	down	miR-181d
RPSA	ENSG00000168028	0,871625609	0,004912811	down	miR-181d
ZZZ3	ENSG00000036549	0,973990946	0,027390975	down	miR-181d
TNKS	ENSG00000173273	0,776801202	0,032516082	down	miR-181d
NRAS	ENSG00000213281	1,409297039	0,049487326	up	miR-181d
FBXO42	ENSG00000037637	1,210464005	0,006644385	up	miR-181d
TMTC3	ENSG00000139324	1,236027677	0,005261538	up	miR-181d
POLR2K	ENSG00000147669	1,082971661	0,049481673	up	miR-181d
RPP14	ENSG00000163684	1,365246367	0,014305025	up	miR-181d
PIAS3	ENSG00000131788	1,329370154	0,000906666	up	miR-181d
RAB1A	ENSG00000138069	1,110771665	0,008078242	up	miR-181d
HACD3	ENSG00000074696	1,114008024	0,014503142	up	miR-181d
SLC35C1	ENSG00000181830	1,258352296	0,031459699	up	miR-181d
MMP24-AS1	ENSG00000126005	0,921994201	0,020803676	down	miR-181d
PCBD2	ENSG00000132570	1,687548402	0,020439217	up	miR-181d
HN1L	ENSG00000206053	1,53959049	0,011760354	up	miR-181d
SETD6	ENSG00000103037	1,890453843	0,007553053	up	miR-181d
RILPL1	ENSG00000188026	1,566286139	0,008547088	up	miR-181d
DMTN	ENSG00000158856	1,661981656	0,029753781	up	miR-181d
NDFIP1	ENSG00000131507	0,96747991	0,026251225	down	miR-181d
SNX4	ENSG00000114520	0,921775027	0,028764734	down	miR-181d
ADO	ENSG00000181915	1,857638527	0,021689329	up	miR-181d
TMEM30B	ENSG00000182107	1,666404617	0,001720717	up	miR-181d
EDA2R	ENSG00000131080	3,187239282	0,0086757	up	miR-181d
GTF2F2	ENSG00000188342	1,019609527	0,017592222	up	miR-181d
AC064836.3	ENSG00000273456	1,512755144	0,012391139	up	miR-181d
FGD5-AS1	ENSG00000225733	1,076049527	0,008578555	up	miR-181d

SECISBP2L	ENSG00000138593	1,107213841	0,010676112	up	miR-181d
PPP6R3	ENSG00000110075	0,67526495	0,03897229	down	miR-181d
SLC1A1	ENSG00000106688	3,440465137	0,011317701	up	miR-181d
AFG3L2	ENSG00000141385	1,030477587	0,002362253	up	miR-181d
ACLY	ENSG00000131473	0,926175079	0,00295562	down	miR-181d
PTGFRN	ENSG00000134247	1,26262975	0,035102842	up	miR-181d
C19orf12	ENSG00000131943	1,30354789	0,014064028	up	miR-181d
TMEM109	ENSG00000110108	0,784791743	0,047335962	down	miR-181d
DDX56	ENSG00000136271	0,895451042	0,01288196	down	miR-181d
METTL2B	ENSG00000165055	0,770064955	0,020768643	down	miR-181d
THEM4	ENSG00000159445	1,363124145	0,019271394	up	miR-181d
PNP	ENSG00000198805	1,410263556	0,008368638	up	miR-181d
ZFX	ENSG00000005889	1,085688057	0,017905433	up	miR-181d
HNRNPA0	ENSG00000177733	1,740169115	0,000131049	up	miR-181d
GTF3C3	ENSG00000119041	1,009584402	0,020240952	up	miR-181d
ATP9A	ENSG00000054793	1,214736756	0,021153375	up	miR-181d
TP53	ENSG00000141510	1,454809834	0,012281195	up	miR-181d
C2orf15	ENSG00000273045	3,516718609	0,009610455	up	miR-181d
ITGA2	ENSG00000164171	2,335222237	0,018366566	up	miR-181d
FAAH2	ENSG00000165591	2,580077807	0,035410535	up	miR-181d
ZNF766	ENSG00000196214	1,282803693	0,032488802	up	miR-181d
METTL15	ENSG00000169519	1,372154095	0,016565271	up	miR-181d
TMPRSS2	ENSG00000184012	2,421700341	0,033539757	up	miR-181d
HCFC1	ENSG00000172534	0,793050845	0,047072773	down	miR-181d
ORC4	ENSG00000115947	1,402825518	0,026175898	up	miR-181d
GSK3B	ENSG00000082701	0,873243446	0,007453936	down	miR-181d
RPL17	ENSG00000265681	1,956472383	0,005830573	up	miR-181d
SNRPD3	ENSG00000100028	0,973012556	0,009175951	down	miR-181d
PRPF19	ENSG00000110107	1,110174072	0,001971482	up	miR-181d
ATF1	ENSG00000123268	1,324650249	0,036435481	up	miR-181d
ETNK2	ENSG00000143845	2,542574408	0,029604716	up	miR-181d
HIST1H4E	ENSG00000276966	3,206229513	0,004585905	up	miR-181d
AC008771.1	ENSG00000249042	2,564570709	0,016843003	up	miR-181d
NENF	ENSG00000117691	0,982831992	0,009996937	down	miR-181d
BUD31	ENSG00000106245	1,286156907	0,003881524	up	miR-181d
ZNF25	ENSG00000175395	2,125398584	0,008340135	up	miR-181d
SMYD2	ENSG00000143499	1,363047341	0,027671983	up	miR-181d
DMXL1	ENSG00000172869	0,851311122	0,008945257	down	miR-181d
ZNF678	ENSG00000181450	1,685145477	0,01324189	up	miR-181d
RBM41	ENSG00000089682	1,258252874	0,000306741	up	miR-181d
NDUFB10	ENSG00000140990	1,116613223	0,028943468	up	miR-181d
GGA2	ENSG00000103365	1,209362358	0,023488961	up	miR-181d
KEAP1	ENSG00000079999	1,140182449	0,023796794	up	miR-181d
COG5	ENSG00000164597	1,396951426	0,015021477	up	miR-181d
DNMBP	ENSG00000107554	1,377132335	0,010159771	up	miR-181d
AKR1B10	ENSG00000198074	2,22691802	0,014777786	up	miR-181d
C18orf32	ENSG00000177576	1,437024401	0,035982003	up	miR-181d
NAGLU	ENSG00000108784	1,035227274	0,026781724	up	miR-181d
NOL4L	ENSG00000197183	0,996187115	0,048622763	down	miR-181d
PAK2	ENSG00000180370	1,36922565	0,027565674	up	miR-181d
FAM32A	ENSG00000105058	1,706565549	0,001407477	up	miR-181d
MAP4K5	ENSG00000012983	0,930669137	0,01690977	down	miR-181d
ELAVL1	ENSG00000066044	1,028899421	0,025714839	up	miR-181d
AKIRIN2	ENSG00000135334	2,11822972	0,005655993	up	miR-181d
NOL6	ENSG00000165271	1,439576453	0,02018583	up	miR-181d
CCNG1	ENSG00000113328	2,038189484	0,012254813	up	miR-181d
ZNF219	ENSG00000165804	2,98379874	0,009039568	up	miR-181d
RAPH1	ENSG00000173166	1,685081012	0,002278506	up	miR-181d
FBXO25	ENSG00000147364	1,719361488	0,013764951	up	miR-181d
FAM102A	ENSG00000167106	1,126963554	0,017907286	up	miR-181d
TSTD1	ENSG00000215845	1,620312363	0,003524534	up	miR-181d
RNF169	ENSG00000166439	1,256306733	0,012735885	up	miR-181d
SPOPL	ENSG00000144228	1,780424306	0,02071346	up	miR-181d
POLR3H	ENSG00000100413	1,568241128	0,002517659	up	miR-181d
SPG11	ENSG00000104133	0,9762998	0,018204474	down	miR-181d
DNAJC27	ENSG00000115137	2,343795878	0,028744966	up	miR-181d
SOCS7	ENSG00000274211	1,520781363	0,016439519	up	miR-181d
MRPL24	ENSG00000143314	1,211374365	0,00508307	up	miR-181d
ATG2B	ENSG00000066739	1,54603773	0,03205266	up	miR-181d
PIM3	ENSG00000198355	3,156485575	0,022370922	up	miR-181d
SHMT2	ENSG00000182199	1,260494467	0,005670872	up	miR-181d
BLVRA	ENSG00000106605	1,290485978	0,019870259	up	miR-181d
PGGT1B	ENSG00000164219	0,686695849	0,027444056	down	miR-181d
SLC15A1	ENSG00000088386	3,060545057	0,020428828	up	miR-181d
PLEKHA3	ENSG00000116095	2,181457858	0,008906573	up	miR-181d
TUBB4B	ENSG00000188229	2,072664333	0,004372911	up	miR-181d
GNPTG	ENSG00000090581	0,988265298	0,027809337	down	miR-181d

PPP4R2	ENSG00000163605	1,666906832	0,009909376	up	miR-181d
DLD	ENSG00000091140	1,197450482	0,011416125	up	miR-181d
YY1AP1	ENSG00000163374	2,001917751	0,000208328	up	miR-181d
URI1	ENSG00000105176	1,088643581	0,010681219	up	miR-181d
CD101	ENSG00000134256	2,495503978	0,028544665	up	miR-181d
STXBP3	ENSG00000116266	1,316679437	0,018881831	up	miR-181d
LINC02256	ENSG00000289083	3,129886321	0,015538652	up	miR-181d
TRAPPC9	ENSG00000167632	1,954581326	0,01003787	up	miR-181d
ETS2	ENSG00000157557	3,464348672	0,002908378	up	miR-181d
RAD50	ENSG00000113522	0,387932149	0,031560392	down	miR-181d
EAPP	ENSG00000129518	1,260002452	0,008258692	up	miR-181d
TSN	ENSG00000211460	1,421818333	0,008060775	up	miR-181d
MRPS18C	ENSG00000163319	1,192528544	0,033255274	up	miR-181d
CMTM8	ENSG00000170293	1,955001221	0,012049135	up	miR-181d
COPS5	ENSG00000121022	0,862816807	0,038395373	down	miR-181d
UQCRQ	ENSG00000164405	1,225326197	0,017549945	up	miR-181d
BAG5	ENSG00000166170	1,093129779	0,025611563	up	miR-181d
TMEM59	ENSG00000116209	1,183676435	0,000893231	up	miR-181d
EPN2	ENSG00000072134	1,265223181	0,008633823	up	miR-181d
XPO7	ENSG00000130227	1,309769705	0,000667841	up	miR-181d
ZDHHC7	ENSG00000153786	1,022148954	0,039961681	up	miR-181d
ATP5C1	ENSG00000165629	1,234903743	0,04496031	up	miR-181d
PRKCE	ENSG00000171132	1,659806169	0,000715375	up	miR-181d
CCDC90B	ENSG00000137500	1,111910649	0,029100755	up	miR-181d
MAGED2	ENSG00000102316	2,273854997	0,016687504	up	miR-181d
GORASP2	ENSG00000115806	0,87410278	0,009537366	down	miR-181d
HNRNPUL2	ENSG00000214753	1,693531822	0,025693773	up	miR-181d
LMNTD2	ENSG00000185522	1,58967002	0,007486077	up	miR-181d
NKRF	ENSG00000186416	2,342015755	0,024045738	up	miR-181d
YBX1	ENSG00000065978	1,594475673	0,007533535	up	miR-181d
MXD4	ENSG00000123933	0,672136179	0,044149528	down	miR-181d
ZNF559	ENSG00000188321	1,434213429	0,045951304	up	miR-181d
RSAD1	ENSG00000136444	2,233728786	0,036454509	up	miR-181d
EPB41L4A-AS1	ENSG00000224032	1,285515309	0,040704471	up	miR-181d
HOOK1	ENSG00000134709	1,393524897	0,034336886	up	miR-181d
SNX5	ENSG00000089006	2,295238929	0,046590335	up	miR-181d
STAU2	ENSG00000040341	0,880014297	0,008551756	down	miR-181d
RNF208	ENSG00000212864	1,39477123	0,034524933	up	miR-181d
RECQL	ENSG00000004700	0,671896856	0,023093454	down	miR-181d
C16orf87	ENSG00000155330	2,016209693	0,004061021	up	miR-181d
TXNDC17	ENSG00000129235	1,660779376	0,002393075	up	miR-181d
NUDC	ENSG00000090273	0,986730104	0,006783604	down	miR-181d
NDUFV2	ENSG00000178127	1,036364927	0,016895529	up	miR-181d
CDH1	ENSG00000039068	1,886384943	0,03562583	up	miR-181d
RNPS1	ENSG00000205937	0,950594221	0,019675908	down	miR-181d
C19orf53	ENSG00000104979	1,685785051	0,030537614	up	miR-181d
HSD17B8	ENSG00000204228	1,502666519	0,007218316	up	miR-181d
NCOA3	ENSG00000124151	1,524622333	0,027723509	up	miR-181d
PRKAB2	ENSG00000131791	1,076270702	0,00880945	up	miR-181d
NRD1	ENSG00000078618	2,174710581	0,025869913	up	miR-181d
TRMT10C	ENSG00000174173	1,019103409	0,049904587	up	miR-181d
PMF1	ENSG00000160783	1,215647987	0,011158878	up	miR-181d
NINJ1	ENSG00000131669	1,202320692	0,022898152	up	miR-181d
HNMT	ENSG00000150540	1,365755171	0,017609379	up	miR-181d
ITPR3	ENSG00000096433	1,511393604	0,001971078	up	miR-181d
EXOSC6	ENSG00000223496	0,89386962	0,033356802	down	miR-181d
CARS2	ENSG00000134905	1,339070855	0,001737814	up	miR-181d
PFKFB3	ENSG00000170525	1,183828065	0,033188536	up	miR-181d
RPL29	ENSG00000162244	1,20188787	0,040612455	up	miR-181d
PHF20	ENSG00000025293	0,997284442	0,039562387	down	miR-181d
FASTKD2	ENSG00000118246	1,522488982	0,016781654	up	miR-181d
SNRPE	ENSG00000182004	1,167824995	0,045238438	up	miR-181d
ZNF317	ENSG00000130803	2,600614034	0,00646461	up	miR-181d
ZBTB10	ENSG00000205189	1,125870003	0,006849642	up	miR-181d
COA4	ENSG00000181924	1,476203698	0,004867328	up	miR-181d
ACAA1	ENSG00000060971	0,844038095	0,014191564	down	miR-181d
IPO8	ENSG00000133704	1,631538424	0,000513529	up	miR-181d
DAG1	ENSG00000173402	1,084680423	0,044154039	up	miR-181d
RRP8	ENSG00000132275	1,105752666	0,034950634	up	miR-181d
IDH1	ENSG00000138413	1,026079649	0,010285815	up	miR-181d
JADE1	ENSG00000077684	1,342435267	0,000612444	up	miR-181d
ZNHIT2	ENSG00000174276	1,770995708	0,038158423	up	miR-181d
TOMM70A	ENSG00000154174	2,201705943	4,87458E-05	up	miR-181d
MTIF3	ENSG00000122033	0,640564859	0,033022732	down	miR-181d
EIF2S2	ENSG00000125977	1,059916752	0,023893879	up	miR-181d
ATP6V1A	ENSG00000114573	1,362390977	0,007865431	up	miR-181d
PEX13	ENSG00000162928	1,92112596	0,001995668	up	miR-181d

TMEM8A	ENSG00000129925	0,819603309	0,029860199	down	miR-181d
KIAA0753	ENSG00000198920	1,461071532	0,003657583	up	miR-181d
NUP88	ENSG00000108559	1,093427745	0,045579406	up	miR-181d
HIST1H2BG	ENSG00000273802	2,744652894	0,023118589	up	miR-181d
NCOA4	ENSG00000266412	1,053136972	0,04142315	up	miR-181d
ZNF3	ENSG00000166526	1,823474315	0,001497371	up	miR-181d
SPIN1	ENSG00000106723	0,815264514	0,030147965	down	miR-181d
TMEM263	ENSG00000151135	1,139023329	0,008568334	up	miR-181d
CSAD	ENSG00000139631	0,84834237	0,00269149	down	miR-181d
BCL9	ENSG00000116128	1,333479369	0,033879445	up	miR-181d
RPS3A	ENSG00000145425	2,938957134	0,036897773	up	miR-181d
TTC38	ENSG00000075234	1,524090503	0,017713305	up	miR-181d
MARVELD1	ENSG00000155254	1,173479459	0,030586474	up	miR-181d
FAM8A1	ENSG00000137414	2,27428261	5,43864E-05	up	miR-181d
CFAP97	ENSG00000164323	0,403022274	0,03815118	down	miR-181d
NCBP2-AS2	ENSG00000270170	2,183211476	0,004671635	up	miR-181d
GJB1	ENSG00000169562	1,233484328	0,047648383	up	miR-181d
CHERP	ENSG00000085872	1,549235817	0,0007597	up	miR-181d
WDR76	ENSG00000092470	2,748487484	0,027349805	up	miR-181d
UXS1	ENSG00000115652	1,063308229	0,008771475	up	miR-181d
PYROXD1	ENSG00000121350	2,021420907	0,005718512	up	miR-181d
PPP1R18	ENSG00000146112	1,5820762	0,019169695	up	miR-181d
ICAM1	ENSG00000090339	0,991707097	0,032589959	down	miR-181d
PCDHGA10	ENSG00000253846	2,162794536	0,040514268	up	miR-181d
TDP2	ENSG00000111802	0,921862733	0,046087841	down	miR-181d
PYGO2	ENSG00000163348	1,003794132	0,023414472	up	miR-181d
THUMPD1	ENSG00000066654	0,674702034	0,033810697	down	miR-181d
HNF4A	ENSG00000101076	1,816826545	0,031052861	up	miR-181d
PSMB1	ENSG00000008018	0,725687796	0,0103958	down	miR-181d
MIB1	ENSG00000101752	1,012855202	0,001322894	up	miR-181d
ATIC	ENSG00000138363	0,749941497	0,009867319	down	miR-181d
POLR1D	ENSG00000186184	1,169275837	0,004470172	up	miR-181d
GNG12	ENSG00000172380	0,931260796	0,018408579	down	miR-181d
EBLN3P	ENSG00000281649	0,81178768	0,008076102	down	miR-181d
INTS7	ENSG00000143493	2,242887554	0,033421192	up	miR-181d
CNN3	ENSG00000117519	1,437560586	0,044330311	up	miR-181d
TANC1	ENSG00000115183	0,944724735	0,001539519	down	miR-181d
PRDX6	ENSG00000117592	1,298873039	0,026416143	up	miR-181d
TRABD	ENSG00000170638	2,256245514	0,010520166	up	miR-181d
LMOD1	ENSG00000163431	2,00642921	0,029724888	up	miR-181d
TMEM204	ENSG00000131634	2,192311811	0,014462883	up	miR-181d
CCDC25	ENSG00000147419	0,802761708	0,033497915	down	miR-181d
AADAC	ENSG00000114771	2,345135066	0,046051262	up	miR-181d
GFM1	ENSG00000168827	1,220188556	0,006067231	up	miR-181d
MRPL43	ENSG00000055950	1,263454071	0,018672884	up	miR-181d
FOSL2	ENSG00000075426	3,312270672	0,032021154	up	miR-181d
EGLN2	ENSG00000269858	1,002650114	0,033974097	up	miR-181d
B4GALT4	ENSG00000121578	1,807379354	0,048536141	up	miR-181d
FLT1	ENSG00000102755	1,253408562	0,001864042	up	miR-181d
GSTO1	ENSG00000148834	0,83136107	0,042514866	down	miR-181d
TMEM9	ENSG00000116857	1,077878908	0,008792786	up	miR-181d
ALKBH5	ENSG00000091542	2,038597193	0,002316866	up	miR-181d
RAB8A	ENSG00000167461	0,662372876	0,037061639	down	miR-181d
AC107068.1	ENSG00000259959	2,207749126	0,031636727	up	miR-181d
VEZF1	ENSG00000136451	0,69363828	0,013088321	down	miR-181d
ADCY9	ENSG00000162104	1,638454098	0,044598849	up	miR-181d
ZUFSP	ENSG00000153975	2,128195745	0,015330007	up	miR-181d
FAM65A	ENSG00000039523	1,733105354	0,025490277	up	miR-181d
MLH3	ENSG00000119684	0,873120622	0,00152708	down	miR-181d
LRRC42	ENSG00000116212	2,041157322	0,021786944	up	miR-181d
TTPA	ENSG00000137561	2,366006915	0,04773851	up	miR-181d
HTRA2	ENSG00000115317	1,564657329	0,007746304	up	miR-181d
IGFBP3	ENSG00000146674	1,562212369	0,025904152	up	miR-181d
PLLP	ENSG00000102934	1,715617419	0,002356252	up	miR-181d
ALDH3A2	ENSG00000072210	1,66509191	0,001666844	up	miR-181d
MARVELD2	ENSG00000152939	1,902185182	0,013807422	up	miR-181d
PDE9A	ENSG00000160191	1,596500266	0,008812323	up	miR-181d
CYLD	ENSG00000083799	1,200517329	0,001001691	up	miR-181d
MYLK	ENSG00000065534	1,50375813	0,001520737	up	miR-181d
HIPK1	ENSG00000163349	0,988254896	0,04637244	down	miR-181d
ANGEL2	ENSG00000174606	1,466815869	0,007801306	up	miR-181d
CYP4V2	ENSG00000145476	2,559470391	0,001409872	up	miR-181d
CHP1	ENSG00000187446	1,557167796	0,005460359	up	miR-181d
GBAS	ENSG00000146729	1,093683092	0,012154957	up	miR-181d
SPPL2B	ENSG00000005206	0,742351303	0,032428263	down	miR-181d
SNX6	ENSG00000129515	1,731572144	0,007913146	up	miR-181d
UBQLN1	ENSG00000135018	1,565879243	0,000147662	up	miR-181d

RBMXL1	ENSG00000213516	1,368865139	0,036109531	up	miR-181d
UBE2V2	ENSG00000169139	0,827763119	0,038959945	down	miR-181d
RNF181	ENSG00000168894	1,438330204	0,000394106	up	miR-181d
RALB	ENSG00000144118	1,370235615	0,011166367	up	miR-181d
PRKCDBP	ENSG00000170955	1,649852599	0,013756523	up	miR-181d
TPP2	ENSG00000134900	1,063160211	0,003212825	up	miR-181d
CBR1	ENSG00000159228	1,663192653	0,010065655	up	miR-181d
FH	ENSG00000091483	2,027039142	0,00784401	up	miR-181d
ZBTB4	ENSG00000174282	1,125345618	0,014060068	up	miR-181d
GPR108	ENSG00000125734	0,941933382	0,017850816	down	miR-181d
VPS4A	ENSG00000132612	1,151830191	0,029278332	up	miR-181d
PDHA1	ENSG00000131828	1,206179554	0,01044099	up	miR-181d
SP3	ENSG00000172845	1,596179051	0,008034138	up	miR-181d
SOCS6	ENSG00000170677	1,455930592	0,007315852	up	miR-181d
SNAP25	ENSG00000132639	4,088488853	0,020512031	up	miR-181d
WASL	ENSG00000106299	0,879617498	0,022786217	down	miR-181d
MLYCD	ENSG00000103150	1,387927506	0,007391048	up	miR-181d
NDUFAB1	ENSG00000004779	0,696859049	0,023484666	down	miR-181d
PDLIM1	ENSG00000107438	2,748748457	0,005785915	up	miR-181d
PSMF1	ENSG00000125818	0,945850058	0,019131325	down	miR-181d
AARS	ENSG00000090861	1,124099094	0,009246908	up	miR-181d
CNOT1	ENSG00000125107	1,333569449	0,020432922	up	miR-181d
FGFR2	ENSG00000066468	2,023913079	0,003260822	up	miR-181d
FAM161B	ENSG00000156050	2,773434129	0,024760345	up	miR-181d
RABL2A	ENSG00000144134	1,794564611	0,010069147	up	miR-181d
FRK	ENSG00000111816	2,316547478	0,009277824	up	miR-181d
SLC35F6	ENSG00000213699	1,03411582	0,008551281	up	miR-181d
ARID1A	ENSG00000117713	1,723546146	0,041413824	up	miR-181d
ASXL2	ENSG00000143970	0,720186475	0,016995167	down	miR-181d
MMADHC	ENSG00000168288	0,912100235	0,012898338	down	miR-181d
CSNK1D	ENSG00000141551	0,9847214	0,002564869	down	miR-181d
CLK1	ENSG00000013441	1,065763266	0,002166509	up	miR-181d
SH3BGRL	ENSG00000131171	1,936229004	0,003017975	up	miR-181d
UPF1	ENSG00000005007	1,627399165	0,009516235	up	miR-181d
RFFL	ENSG00000092871	1,154708936	0,011162624	up	miR-181d
PIGF	ENSG00000151665	2,447818255	0,043664639	up	miR-181d
LOC100996717	ENSG00000271383	1,480428338	0,023845324	up	miR-181d
CCDC125	ENSG00000183323	1,606411832	0,001072215	up	miR-181d
ADI1	ENSG00000182551	1,807924806	0,002852647	up	miR-181d
BAG3	ENSG00000151929	1,861782802	0,007946259	up	miR-181d
FKBP4	ENSG00000004478	1,219121783	0,017548775	up	miR-181d
FBXO17	ENSG00000269190	1,731364821	0,033658156	up	miR-181d
CERS6	ENSG00000172292	1,324454705	0,025710601	up	miR-181d
ELMO3	ENSG00000102890	2,419434017	0,020354791	up	miR-181d
SLIRP	ENSG00000119705	1,267650614	0,006348542	up	miR-181d
CCDC58	ENSG00000160124	1,73954935	0,023314021	up	miR-181d
P4HTM	ENSG00000178467	1,139556713	0,041704308	up	miR-181d
SHMT1	ENSG00000176974	1,974897195	0,018380155	up	miR-181d
AP1G1	ENSG00000166747	1,029345081	0,012723669	up	miR-181d
GPR137B	ENSG00000077585	1,462237874	0,023511208	up	miR-181d
FAM63B	ENSG00000128923	0,839485648	0,048669462	down	miR-181d
ETFB	ENSG00000105379	1,046259975	0,006339732	up	miR-181d
KDM3A	ENSG00000115548	1,47854336	0,031912684	up	miR-181d
CYB561	ENSG00000008283	1,986196099	0,00232097	up	miR-181d
ECHDC1	ENSG00000093144	1,109317549	0,017132362	up	miR-181d
TMC4	ENSG00000167608	1,348267086	0,004694983	up	miR-181d
SGCB	ENSG00000163069	1,292019367	0,009346623	up	miR-181d
CS	ENSG00000062485	0,850152105	0,016901983	down	miR-181d
SENP3	ENSG00000161956	0,770634304	0,024260157	down	miR-181d
ZBTB33	ENSG00000177485	2,353883874	5,94909E-05	up	miR-181d
PLXNB1	ENSG00000164050	1,477813195	0,020712866	up	miR-181d
LYPLA2	ENSG00000011009	1,179963917	0,010634491	up	miR-181d
PPP1CB	ENSG00000213639	0,968640088	0,026999055	down	miR-181d
SEMA5B	ENSG00000082684	2,890546595	0,023230343	up	miR-181d
GOT2	ENSG00000125166	0,874809179	0,031062855	down	miR-181d
MYEOV2	ENSG00000172428	0,930400034	0,036016145	down	miR-181d
HIBCH	ENSG00000198130	0,864986951	0,004356442	down	miR-181d
FBXO11	ENSG00000138081	0,311252844	0,048852543	down	miR-181d
STX3	ENSG00000166900	0,769986871	0,020581892	down	miR-181d
RNF145	ENSG00000145860	2,545386919	0,000406507	up	miR-181d
SYNJ2BP	ENSG00000213463	0,975643716	0,006708645	down	miR-181d
BRK1	ENSG00000254999	1,402544477	0,014245828	up	miR-181d
CCL3	ENSG00000277632	-0,974773859	0,035920541	down	miR-181d
NAA50	ENSG00000121579	1,277316418	0,009339194	up	miR-181d
SAP18	ENSG00000150459	1,114836347	0,011902974	up	miR-181d
TMEM177	ENSG00000144120	1,47220369	0,013058613	up	miR-181d
NBR1	ENSG00000188554	0,809179283	0,014775598	down	miR-181d

ARMC1	ENSG00000104442	0,761623778	0,033531454	down	miR-181d
UBAC2	ENSG00000134882	1,175227963	0,040749366	up	miR-181d
HOOK2	ENSG00000095066	0,791205576	0,034741403	down	miR-181d
GCN1	ENSG00000089154	0,76429846	0,025159917	down	miR-181d
YES1	ENSG00000176105	1,019035675	0,015320535	up	miR-181d
HAUS6	ENSG00000147874	1,208610793	0,025413104	up	miR-181d
RIOK1	ENSG00000124784	0,735534478	0,042003799	down	miR-181d
SERPINA5	ENSG00000188488	3,403559681	0,021293696	up	miR-181d
ACAD9	ENSG00000177646	1,587223715	0,005991056	up	miR-181d
FAM103A1	ENSG00000169612	1,580294531	0,043252466	up	miR-181d
STX6	ENSG00000135823	1,366216508	0,008151731	up	miR-181d
BLOC1S1	ENSG00000135441	1,178574474	0,030454659	up	miR-181d
SMAD4	ENSG00000141646	1,614826174	0,006680703	up	miR-181d
ATP6AP1	ENSG00000071553	6,114678798	0,029274583	up	miR-181d
NUCB2	ENSG00000070081	1,40488331	0,002300154	up	miR-181d
NR2F6	ENSG00000160113	0,833268763	0,010603953	down	miR-181d
DCTPP1	ENSG00000179958	1,409149112	0,017179932	up	miR-181d
KIAA1804	ENSG00000143674	1,833947404	0,009526103	up	miR-181d
SLC39A6	ENSG00000141424	0,7452337	0,034001914	down	miR-181d
PTEN	ENSG00000171862	0,847670346	0,02344528	down	miR-181d
ZER1	ENSG00000160445	1,16992298	0,029744935	up	miR-181d
LRCH3	ENSG00000186001	0,801547572	0,009280485	down	miR-181d
MAL2	ENSG00000147676	1,396501347	0,006713001	up	miR-181d
ZNF581	ENSG00000171425	1,575320992	0,010362108	up	miR-181d
GLO1	ENSG00000124767	1,193980093	0,027634209	up	miR-181d
MAP3K1	ENSG00000095015	1,019763664	0,036984888	up	miR-181d
RNF10	ENSG00000022840	1,340161288	0,014207028	up	miR-181d
RAP2C	ENSG00000123728	1,685632268	0,009493148	up	miR-181d
PARG	ENSG00000227345	1,84387664	0,014508616	up	miR-181d
BCAR1	ENSG00000050820	1,362659066	0,006774395	up	miR-181d
DIEXF	ENSG00000117597	1,15445577	0,031795495	up	miR-181d
NPTN	ENSG00000156642	1,223402563	0,030390971	up	miR-181d
FZR1	ENSG00000105325	1,39467975	0,006862886	up	miR-181d
ATP6V1E2	ENSG00000250565	1,796134646	0,010735182	up	miR-181d
NMD3	ENSG00000169251	0,972903786	0,013189919	down	miR-181d
ERI2	ENSG00000196678	1,592548703	0,022603402	up	miR-181d
CDIPT	ENSG00000103502	1,189665318	0,004017982	up	miR-181d
CNIH4	ENSG00000143771	1,292783298	0,013148455	up	miR-181d
CDYL2	ENSG00000166446	1,323684939	0,031110865	up	miR-181d
TSNAX	ENSG00000116918	1,056306444	0,005607754	up	miR-181d
TKFC	ENSG00000149476	1,463453718	0,011144778	up	miR-181d
TMEM126B	ENSG00000171204	1,283853919	0,030679409	up	miR-181d
FAM83H	ENSG00000180921	1,284067415	0,001746915	up	miR-181d
MRPL55	ENSG00000162910	1,097950528	0,001166808	up	miR-181d
TET2	ENSG00000168769	0,894571328	0,013634668	down	miR-181d
CHMP4C	ENSG00000164695	2,381640669	0,000688363	up	miR-181d
ACOX1	ENSG00000161533	0,930369197	0,002058269	down	miR-181d
LBR	ENSG00000143815	1,510599868	0,001264822	up	miR-181d
LRP6	ENSG00000070018	0,903867363	0,043194597	down	miR-181d
FKBP5	ENSG00000096060	1,493276804	0,028871754	up	miR-181d
SLC27A5	ENSG00000083807	4,047579633	0,013315607	up	miR-181d
ASUN	ENSG00000064102	1,229237207	0,003461351	up	miR-181d
TANC2	ENSG00000170921	1,096431856	0,014934456	up	miR-181d
ASAP2	ENSG00000151693	1,872246979	0,000752394	up	miR-181d
SH3BP5L	ENSG00000175137	1,787435732	0,016843229	up	miR-181d
SMAD2	ENSG00000175387	0,535722458	0,045210057	down	miR-181d
PPM1B	ENSG00000138032	1,422389572	0,005349114	up	miR-181d
P3H2	ENSG00000090530	1,428914386	0,048854404	up	miR-181d
CPSF6	ENSG00000111605	1,14651594	0,017926739	up	miR-181d
SCOC	ENSG00000153130	0,753353926	0,028895367	down	miR-181d
RBBP5	ENSG00000117222	1,459953204	0,007242247	up	miR-181d
ZNF189	ENSG00000136870	1,641544121	0,04157333	up	miR-181d
CARD10	ENSG00000100065	0,940077679	0,037044496	down	miR-181d
FAM127B	ENSG00000203950	1,70064262	0,017725539	up	miR-181d
ATG9A	ENSG00000198925	1,024572466	0,00737135	up	miR-181d
SNRNP48	ENSG00000168566	1,038062316	0,003726746	up	miR-181d
GAS2L1	ENSG00000185340	1,21534984	0,031807129	up	miR-181d
MED6	ENSG00000133997	1,135001431	0,014472465	up	miR-181d
ARHGAP32	ENSG00000134909	1,012587245	0,048452286	up	miR-181d
CHKB	ENSG00000100288	2,123559122	0,002670186	up	miR-181d
EIF4EBP3	ENSG00000243056	1,006570965	0,037324498	up	miR-181d
METTL7B	ENSG00000170439	1,628760566	0,043306216	up	miR-181d
C17orf89	ENSG00000224877	1,15066987	0,001561775	up	miR-181d
MAPK6	ENSG00000069956	1,021136348	0,00990017	up	miR-181d
CNOT11	ENSG00000158435	1,201958906	0,002126188	up	miR-181d
GPALPP1	ENSG00000133114	1,234410554	0,016195347	up	miR-181d
AC011498.4	ENSG00000267385	2,841186127	0,026526439	up	miR-181d

CACFD1	ENSG00000160325	0,985001894	0,039479126	down	miR-181d
YWHAG	ENSG00000170027	0,227675137	0,009293421	down	miR-181d
EIF2AK1	ENSG00000086232	1,075334586	0,014071533	up	miR-181d
PGRMC1	ENSG00000101856	0,625219883	0,006217419	down	miR-181d
TMEM176B	ENSG00000106565	1,352713532	0,038647137	up	miR-181d
ING5	ENSG00000168395	1,335014103	0,006374599	up	miR-181d
FOXM1	ENSG00000111206	2,256096142	0,016601083	up	miR-181d
SENP5	ENSG00000119231	1,302806201	0,022814803	up	miR-181d
CUL3	ENSG00000036257	0,76874823	0,041337063	down	miR-181d
SLC25A5	ENSG00000005022	1,269002515	0,023459749	up	miR-181d
FXYD5	ENSG00000089327	1,415728084	0,009626006	up	miR-181d
CETN2	ENSG00000147400	1,388200952	0,002610313	up	miR-181d
PFKP	ENSG00000067057	1,187134979	0,002454359	up	miR-181d
DNPEP	ENSG00000123992	0,771005283	0,022014815	down	miR-181d
ETV4	ENSG00000175832	2,222030713	0,00085083	up	miR-181d
PSEN2	ENSG00000143801	1,978025611	0,00345363	up	miR-181d
C1orf115	ENSG00000162817	1,412800654	0,041637643	up	miR-181d
COPZ1	ENSG00000111481	1,872440325	0,000883075	up	miR-181d
PCYOX1	ENSG00000116005	1,044143322	0,005786289	up	miR-181d
PPP2CA	ENSG00000113575	1,973049231	0,000329428	up	miR-181d
MFAP1	ENSG00000140259	0,927827697	0,02705952	down	miR-181d
ATOX1	ENSG00000177556	0,797053274	0,034313631	down	miR-181d
FAM168B	ENSG00000152102	1,90878369	0,000608487	up	miR-181d
FAM161A	ENSG00000170264	1,464841686	0,026404169	up	miR-181d
ACSS3	ENSG00000111058	2,198207971	0,025243308	up	miR-181d
CYP4F11	ENSG00000171903	1,559630652	0,048788437	up	miR-181d
FAM195A	ENSG00000172366	1,21422653	0,041111041	up	miR-181d
QTRTD1	ENSG00000151576	1,186535088	0,007727855	up	miR-181d
TMEM74B	ENSG00000125895	2,787605035	0,033357218	up	miR-181d
AC093726.1	ENSG00000272760	1,969440682	0,010281965	up	miR-181d
IDI1	ENSG00000067064	1,626562994	0,010719444	up	miR-181d
FAM73B	ENSG00000148343	1,413220759	0,012192592	up	miR-181d
DEDD	ENSG00000158796	1,622753499	0,002331102	up	miR-181d
PLS1	ENSG00000120756	1,167770489	0,014264694	up	miR-181d
LPCAT1	ENSG00000153395	2,01073532	0,00507144	up	miR-181d
POLR2I	ENSG00000105258	1,323845869	0,009407056	up	miR-181d
ZBED6	ENSG00000257315	0,922858542	0,020549691	down	miR-181d
PPP4R1	ENSG00000154845	1,371752586	0,027868332	up	miR-181d
ZXDB	ENSG00000198455	2,964140824	0,034085334	up	miR-181d
POLDIP2	ENSG00000004142	0,992173446	0,007861699	down	miR-181d
ZNF43	ENSG00000198521	1,169635202	0,040788717	up	miR-181d
CLDN12	ENSG00000157224	1,189737039	0,020125253	up	miR-181d
BPHL	ENSG00000137274	1,445550034	0,016383202	up	miR-181d
GATM	ENSG00000171766	1,697961132	0,012527295	up	miR-181d
HADH	ENSG00000138796	1,782433604	0,048967057	up	miR-181d
PCBD1	ENSG00000166228	1,164695316	0,009288563	up	miR-181d
DAPK3	ENSG00000167657	1,245865984	0,00453236	up	miR-181d
YIPF2	ENSG00000130733	0,965686875	0,005277664	down	miR-181d
DCAKD	ENSG00000172992	1,349911527	0,011095597	up	miR-181d
WAC-AS1	ENSG00000254635	1,398703983	0,001908847	up	miR-181d
COA5	ENSG00000183513	0,9897671	0,03325429	down	miR-181d
TIMM23	ENSG00000265354	1,251556672	0,02309299	up	miR-181d
DCUN1D1	ENSG00000043093	1,418321923	0,009348452	up	miR-181d
KCNK1	ENSG00000135750	1,326411115	0,049174558	up	miR-181d
LRRC16A	ENSG00000079691	1,247435646	0,007072645	up	miR-181d
EFHD2	ENSG00000142634	5,980755605	0,011956144	up	miR-181d
PSMB5	ENSG00000100804	1,211489544	0,001619353	up	miR-181d
GUCY1A2	ENSG00000152402	1,496202695	0,047757357	up	miR-181d
BCOR	ENSG00000183337	2,049522507	0,000293342	up	miR-181d
UXT	ENSG00000126756	1,359673524	0,017248471	up	miR-181d
ATXN1L	ENSG00000224470	1,564146818	0,049388681	up	miR-181d
FLVCR1	ENSG00000162769	3,222126364	0,001009783	up	miR-181d
ATP2C1	ENSG00000017260	1,25267688	0,02643113	up	miR-181d
NMT2	ENSG00000152465	1,181291416	0,042295936	up	miR-181d
MRPS35	ENSG00000061794	0,851362553	0,022164111	down	miR-181d
BOK	ENSG00000176720	1,345634283	0,046772381	up	miR-181d
TBC1D15	ENSG00000121749	1,798143701	0,002778386	up	miR-181d
ZNF37A	ENSG00000075407	1,097810795	0,014646677	up	miR-181d
ADCK3	ENSG00000163050	2,127198907	0,009303019	up	miR-181d
C4orf3	ENSG00000164096	1,039037661	0,009743621	up	miR-181d
NVL	ENSG00000143748	0,90218791	0,001758954	down	miR-181d
KIF3A	ENSG00000131437	0,736836513	0,041510858	down	miR-181d
LANCL2	ENSG00000132434	2,850264152	0,002115982	up	miR-181d
ALG10B	ENSG00000175548	2,700927538	0,020805593	up	miR-181d
IPO5	ENSG00000065150	1,000686183	0,011706232	up	miR-181d
ABHD2	ENSG00000140526	1,211093139	0,018040649	up	miR-181d
MTL5	ENSG00000132749	1,81692538	0,034150297	up	miR-181d

DGCR8	ENSG00000128191	1,167524455	0,040229455	up	miR-181d
POMP	ENSG00000132963	1,999001666	0,002292922	up	miR-181d
FTCD	ENSG00000160282	4,259564626	0,02409988	up	miR-181d
ARL6IP6	ENSG00000177917	2,909583443	0,013889766	up	miR-181d
SLC39A9	ENSG00000029364	1,017033513	0,026894581	up	miR-181d
NOXA1	ENSG00000188747	2,935458907	0,003642664	up	miR-181d
RANBP10	ENSG00000141084	1,60684905	0,03636765	up	miR-181d
IFT172	ENSG00000138002	2,02245675	0,027682489	up	miR-181d
HIST1H2AC	ENSG00000180573	1,585973361	0,008164463	up	miR-181d
MYH14	ENSG00000105357	1,381021409	0,012321143	up	miR-181d
KIAA1147	ENSG00000257093	0,968263189	0,045174366	down	miR-181d
C2orf54	ENSG00000172478	2,770351109	0,031216883	up	miR-181d
PAFAH2	ENSG00000158006	1,7361959	0,031582497	up	miR-181d
INO80B	ENSG00000115274	1,436098763	0,026084474	up	miR-181d
CEP295	ENSG00000166004	0,800232089	0,008461892	down	miR-181d
SMS	ENSG00000102172	1,360468894	0,037978456	up	miR-181d
TLE1	ENSG00000196781	1,760868941	0,001660547	up	miR-181d
CCDC71	ENSG00000177352	2,522663857	0,026558644	up	miR-181d
RAB3GAP1	ENSG00000115839	1,129624691	0,022901379	up	miR-181d
NPM2	ENSG00000158806	2,827801684	0,007095591	up	miR-181d
RILP	ENSG00000167705	1,893405612	0,023685291	up	miR-181d
S100A16	ENSG00000188643	0,687264579	0,034418597	down	miR-181d
FAM214B	ENSG00000005238	2,088617603	0,005633086	up	miR-181d
ILVBL	ENSG00000105135	1,416941249	0,0389949	up	miR-181d
AKR1A1	ENSG00000117448	3,650890005	0,008477016	up	miR-181d
PPTC7	ENSG00000196850	1,245184889	0,018239068	up	miR-181d
DNM1L	ENSG00000087470	1,326624527	0,005076844	up	miR-181d
PAXIP1-AS1	ENSG00000273344	2,632845522	0,034028157	up	miR-181d
PTPN3	ENSG00000070159	1,193343241	0,043940488	up	miR-181d
TSPAN15	ENSG00000099282	1,830147036	0,01896983	up	miR-181d
EPHX1	ENSG00000143819	1,160828235	0,018663184	up	miR-181d
LGR4	ENSG00000205213	1,894868976	0,001687751	up	miR-181d
SNX20	ENSG00000167208	1,607893552	0,046521766	up	miR-181d
GSPT2	ENSG00000189369	2,28254518	0,000939272	up	miR-181d
NUDT5	ENSG00000165609	1,02853537	0,046445369	up	miR-181d
PAAF1	ENSG00000175575	1,283323302	0,040628618	up	miR-181d
STK16	ENSG00000115661	1,311834408	0,032586216	up	miR-181d
ANKHD1	ENSG00000131503	1,978170323	0,00379399	up	miR-181d
EPB41L5	ENSG00000115109	2,104739587	0,00521256	up	miR-181d
C19orf70	ENSG00000174917	2,336770395	0,000396666	up	miR-181d
GPRIN1	ENSG00000169258	1,500695797	0,04161825	up	miR-181d
ZNF136	ENSG00000196646	1,351294774	0,042142485	up	miR-181d
ZNF785	ENSG00000197162	1,264160541	0,029966742	up	miR-181d
DPY19L3	ENSG00000178904	2,299425149	0,008060218	up	miR-181d
UGT2B15	ENSG00000196620	5,107280648	0,009359291	up	miR-181d
SNAP23	ENSG00000092531	0,963136082	0,032500436	down	miR-181d
FNDC4	ENSG00000115226	2,767793555	0,017934871	up	miR-181d
MAP1LC3A	ENSG00000101460	1,500006139	0,015848255	up	miR-181d
SLC35C2	ENSG00000080189	1,227414897	0,005780229	up	miR-181d
TOR4A	ENSG00000198113	1,402942423	0,002326462	up	miR-181d
TMEM185B	ENSG00000226479	2,788251591	0,005061664	up	miR-181d
SELENBP1	ENSG00000143416	1,247856849	0,027500199	up	miR-181d
MPND	ENSG00000008382	1,101122	0,03884098	up	miR-181d
ACD	ENSG00000102977	1,297137833	0,043187094	up	miR-181d
C12orf75	ENSG00000235162	2,294201931	0,014470063	up	miR-181d
RPH3AL	ENSG00000181031	2,004851876	0,02296749	up	miR-181d
MST1	ENSG00000173531	2,402831545	0,002611091	up	miR-181d
TMEM44	ENSG00000145014	1,410767246	0,033058505	up	miR-181d
NADK2	ENSG00000152620	1,610034602	0,006139488	up	miR-181d
FCN3	ENSG00000142748	3,996147053	0,022884934	up	miR-181d
FBXL4	ENSG00000112234	2,299904154	0,009351038	up	miR-181d
ARSE	ENSG00000157399	2,592612113	0,014478371	up	miR-181d
ZNF593	ENSG00000142684	1,421504045	0,015711746	up	miR-181d
AL451165.2	ENSG00000272288	3,524663667	0,027005112	up	miR-181d
AP000253.1	ENSG00000234509	2,011206123	0,020331465	up	miR-181d
USP49	ENSG00000164663	1,803424246	0,009396919	up	miR-181d
FAM66C	ENSG00000226711	2,826426147	0,025871951	up	miR-181d
C6orf141	ENSG00000197261	2,722048184	0,018186057	up	miR-181d
ARHGEF5	ENSG00000050327	1,475173138	0,00519372	up	miR-181d
E2F6	ENSG00000169016	2,613199678	0,012698672	up	miR-181d
SRP9	ENSG00000143742	2,208405984	0,004978933	up	miR-181d
BEND3	ENSG00000178409	2,733026039	0,031759733	up	miR-181d
IRX3	ENSG00000177508	3,368409169	0,026088529	up	miR-181d
PRELID2	ENSG00000186314	2,420905083	0,042898762	up	miR-181d
SYBU	ENSG00000147642	2,399854954	0,046017766	up	miR-181d
TMEM92	ENSG00000167105	2,652941798	0,00925936	up	miR-181d
CCER2	ENSG00000262484	2,136252983	0,018019918	up	miR-181d

AC099518.2	ENSG00000261357	2,873612886	0,041231811	up	miR-181d
AC007938.3	ENSG00000270953	1,645936042	0,035896685	up	miR-181d
IGFBP1	ENSG00000146678	3,493989558	0,043314798	up	miR-181d
AMY2B	ENSG00000240038	3,007226928	0,022498535	up	miR-181d
ZNF256	ENSG00000152454	1,902589131	0,030992526	up	miR-181d
IRX2	ENSG00000170561	-2,219227689	0,027127374	down	miR-181d
NDUFAF1	ENSG00000137806	-1,749292937	0,028868975	down	miR-181d
UBR7	ENSG00000012963	-0,943443378	0,044063877	down	miR-181d
TRMT13	ENSG00000122435	1,014936289	0,00262015	up	miR-181d
HKR1	ENSG00000181666	1,074937757	0,036616697	up	miR-181d
SLC25A46	ENSG00000164209	0,821755905	0,036866508	down	miR-181d
AHDC1	ENSG00000126705	1,096615908	0,043832093	up	miR-181d
SCRIB	ENSG00000180900	1,229440381	0,01484774	up	miR-181d
TGFBR1	ENSG00000106799	0,898099058	0,03820927	down	miR-181d
FAM188A	ENSG00000148481	1,469486971	0,032149856	up	miR-181d
ZNF865	ENSG00000261221	1,609226956	0,030975863	up	miR-181d
SFXN4	ENSG00000183605	0,938373199	0,025382742	down	miR-181d
TMEM258	ENSG00000134825	2,174157197	0,005717558	up	miR-181d
NTAN1	ENSG00000157045	1,390392133	0,032144365	up	miR-181d
MED29	ENSG00000063322	0,968362018	0,030341336	down	miR-181d
DAZAP2	ENSG00000183283	0,958624585	0,022334843	down	miR-181d
TM7SF3	ENSG00000064115	1,027963618	0,037565886	up	miR-181d
KLHL29	ENSG00000119771	1,380776688	0,039905282	up	miR-181d
NCOA2	ENSG00000140396	1,209885083	0,008193269	up	miR-181d
COX6A1	ENSG00000111775	1,860986504	0,004583172	up	miR-181d
PROSER2	ENSG00000148426	2,695253738	0,013925986	up	miR-181d
C11orf54	ENSG00000182919	0,91623587	0,019654729	down	miR-181d
RAB3D	ENSG00000105514	1,28904312	0,007153195	up	miR-181d
RNF7	ENSG00000114125	0,940364122	0,0238932	down	miR-181d
FAM199X	ENSG00000123575	1,500812218	0,032450952	up	miR-181d
ATF2	ENSG00000115966	0,860793232	0,016677902	down	miR-181d
IQCK	ENSG00000174628	1,633558685	0,018579507	up	miR-181d
AKAP6	ENSG00000151320	-1,931772052	0,027162879	down	miR-181c
PRNCR1	ENSG00000282961	-2,009969631	0,045485028	down	miR-181c
AC104653.1	ENSG00000228857	-2,005209213	0,02356805	down	miR-181c
FANCD2	ENSG00000144554	-1,083069618	0,016078332	down	miR-181c
HOXA10	ENSG00000253293	-2,111729969	0,004235417	down	miR-181c
RGMA	ENSG00000182175	-2,049181329	0,004453382	down	miR-181c
MECR	ENSG00000116353	-1,021514705	0,03388029	down	miR-181c
HEATR3	ENSG00000155393	-1,355498802	0,009713255	down	miR-181c
MICAL3	ENSG00000243156	-0,634310385	0,034267878	down	miR-181c
MRFAP1	ENSG00000179010	1,589747492	0,001908633	up	miR-181c
DAZAP2	ENSG00000183283	1,177683559	0,008961337	up	miR-181c
ZMYND15	ENSG00000141497	-1,981098697	0,000490311	down	miR-181c
AC007336.1	ENSG00000261997	-1,574796703	0,032491248	down	miR-181c
GPRC5A	ENSG00000013588	-3,023103224	0,008361812	down	miR-181c
NCKAP1L	ENSG00000123338	-0,676263194	0,005158316	down	miR-181c
FADS1	ENSG00000149485	-0,530335714	0,003615083	down	miR-181c
ZC3H3	ENSG00000014164	-1,124686709	0,025581964	down	miR-181c
TMEM106B	ENSG00000106460	0,470828107	0,026168789	down	miR-181c
UNC13D	ENSG00000092929	-1,043729666	0,028825832	down	miR-181c
MAGI1	ENSG00000151276	-0,359885459	0,036449192	down	miR-181c
PPP2R5B	ENSG00000068971	-1,194434435	0,00185214	down	miR-181c
SGK223	ENSG00000275342	-1,044053374	0,012152841	down	miR-181c
WWOX	ENSG00000186153	-0,874084524	0,021923647	down	miR-181c
TAF1A	ENSG00000143498	-1,580541364	0,038319446	down	miR-181c
GLI3	ENSG00000106571	-1,239844838	0,005600817	down	miR-181c
SIRT1	ENSG00000096717	-0,820927497	0,049736872	down	miR-181c
TACC2	ENSG00000138162	-1,442901785	0,000351475	down	miR-181c
ZNF714	ENSG00000160352	-0,866379648	0,027641781	down	miR-181c
AL133367.1	ENSG00000260285	-2,358449073	0,002149438	down	miR-181c
TUBE1	ENSG00000074935	-1,258350823	0,036317702	down	miR-181c
ADCY4	ENSG00000129467	-0,878453697	0,023560913	down	miR-181c
AC109466.1	ENSG00000241956	-2,424501671	0,030773224	down	miR-181c
TOP3A	ENSG00000177302	-0,845879788	0,0054525	down	miR-181c
ZCCHC8	ENSG00000033030	-0,579175195	0,033177819	down	miR-181c
NEK8	ENSG00000160602	-0,928802524	0,021164068	down	miR-181c
LINC00971	ENSG00000242641	-1,8427385	0,039451674	down	miR-181c
FAM84A	ENSG00000162981	-1,152845728	0,009610163	down	miR-181c
JAKMIP2	ENSG00000176049	-1,544124787	0,032779682	down	miR-181c
KCNK3	ENSG00000171303	-2,507245912	0,016327473	down	miR-181c
AC005523.2	ENSG00000269604	-2,770808653	0,041631778	down	miR-181c
AC003986.2	ENSG00000232821	-3,408963642	0,02579972	down	miR-181c
TENM3	ENSG00000218336	-1,756877978	0,018506512	down	miR-181c
LRRC15	ENSG00000172061	-1,625396363	0,032070807	down	miR-181c
TSPAN1	ENSG00000117472	-1,741085035	0,043496501	down	miR-181c
CBLC	ENSG00000142273	-2,04555127	0,002658917	down	miR-181c

RIMS2	ENSG00000176406	-2,221890397	0,04843935	down	miR-181c
MUC16	ENSG00000181143	-4,798299211	0,001827155	down	miR-181c
DEFB118	ENSG00000131068	-2,95222908	0,022199375	down	miR-181c
AL158163.2	ENSG00000278601	-1,107177751	0,029401311	down	miR-181c
UQCRHL	ENSG00000233954	-1,671239692	0,039003095	down	miR-181c
AL137786.1	ENSG00000258702	-3,371800499	0,019774283	down	miR-181c
MAEL	ENSG00000143194	-3,584388044	0,018088178	down	miR-181c
VILL	ENSG00000136059	-1,939889012	0,011391523	down	miR-181c
MORN3	ENSG00000139714	-1,661031952	0,019080468	down	miR-181c
CDC42EP5	ENSG00000167617	-1,236592403	0,018920004	down	miR-181c
SCIN	ENSG00000006747	-2,015351453	0,009921003	down	miR-181c
CLLU1	ENSG00000257127	-2,711221354	0,01712959	down	miR-181c
PLEKHS1	ENSG00000148735	-1,670477352	0,02078108	down	miR-181c
DPP6	ENSG00000130226	-2,784443422	0,009599699	down	miR-181c
Z95114.1	ENSG00000279217	-2,639662619	0,01719844	down	miR-181c
SLC4A8	ENSG00000050438	-1,806151802	0,038444767	down	miR-181c
AC025048.4	ENSG00000267416	-2,853321644	0,006923565	down	miR-181c
C1orf110	ENSG00000185860	-2,095906422	0,020450634	down	miR-181c
AL031595.2	ENSG00000280011	-2,615938612	0,042148623	down	miR-181c
SLC35G3	ENSG00000164729	-2,394023856	0,04965902	down	miR-181c
SLC6A20	ENSG00000163817	-2,003173189	0,017234117	down	miR-181c
NOS1	ENSG00000089250	-2,537581969	0,005706949	down	miR-181c
LINC01224	ENSG00000269416	-3,214687305	0,010385083	down	miR-181c
POU2F3	ENSG00000137709	-1,720588777	0,044913341	down	miR-181c
CTCFL	ENSG00000124092	-1,468795522	0,013227429	down	miR-181c
DLGAP1	ENSG00000170579	-2,657488175	0,034976374	down	miR-181c
EML5	ENSG00000165521	-3,036695407	0,004981814	down	miR-181c
GABRB1	ENSG00000163288	-3,3786742	0,018328048	down	miR-181c
MIR133A1HG	ENSG00000265142	-3,174186367	0,044761409	down	miR-181c
SH3BGR	ENSG00000185437	1,168872703	0,010345268	up	miR-181c
CSTA	ENSG00000121552	1,319066229	0,032235251	up	miR-181c
PPP1R1C	ENSG00000150722	1,840250703	0,047295573	up	miR-181c
APOBEC3H	ENSG00000100298	1,395897118	0,023922939	up	miR-181c
KLLN	ENSG00000227268	1,100700228	0,020042371	up	miR-181c
INTS12	ENSG00000138785	2,642006933	0,000272122	up	miR-181c
E2F7	ENSG00000165891	3,469250952	0,042485381	up	miR-181c
SYNGR1	ENSG00000100321	1,81441566	0,008906679	up	miR-181c
TAS2R19	ENSG00000212124	2,237434245	0,000180253	up	miR-181c
VSIG8	ENSG00000243284	1,312748323	0,037371744	up	miR-181c
AC104964.4	ENSG00000272505	0,929444699	0,029584945	down	miR-181c
LINC02015	ENSG00000231574	2,408889579	0,049592627	up	miR-181c
LOH12CR2	ENSG00000205791	1,06456623	0,008487958	up	miR-181c
LMO7-AS1	ENSG00000261105	1,048936883	0,04410998	up	miR-181c
RTN4RL1	ENSG00000185924	3,148875288	0,004245395	up	miR-181c
PPM1J	ENSG00000155367	1,77771223	0,005940734	up	miR-181c
AC078846.1	ENSG00000273329	2,18852378	0,004498879	up	miR-181c
BTBD1	ENSG00000064726	2,235387324	0,002103204	up	miR-181c
TMEM132E	ENSG00000181291	2,217725909	0,035827056	up	miR-181c
ACTL10	ENSG00000288649	2,109266139	0,010690187	up	miR-181c
TAF9B	ENSG00000187325	2,056732934	0,029111533	up	miR-181c
PCDHGA7	ENSG00000253537	1,386686454	0,01595457	up	miR-181c
NHEJ1	ENSG00000187736	1,273473518	0,049642316	up	miR-181c
CDK5R1	ENSG00000176749	1,513174471	0,030984195	up	miR-181c
BATF3	ENSG00000123685	2,095370643	0,002528503	up	miR-181c
LINC01637	ENSG00000237476	1,893004336	0,011985432	up	miR-181c
COQ10A	ENSG00000135469	1,206655755	0,022714394	up	miR-181c
SHC4	ENSG00000185634	1,452713352	0,015600377	up	miR-181c
NR2F1-AS1	ENSG00000237187	1,158457196	0,03575727	up	miR-181c
ZNF253	ENSG00000256771	1,951511282	0,007892454	up	miR-181c
CSF2RB	ENSG00000100368	1,122698693	0,045813077	up	miR-181c
ZNF175	ENSG00000105497	1,886486335	0,001658427	up	miR-181c
DEFB1	ENSG00000164825	3,069023485	0,000852893	up	miR-181c
GCLC	ENSG00000001084	1,866997594	0,000153393	up	miR-181c
NAP1L2	ENSG00000186462	1,206743368	0,008650437	up	miR-181c
AMOTL1	ENSG00000166025	0,734630646	0,046112195	down	miR-181c
SHANK2	ENSG00000162105	1,183851707	0,020293612	up	miR-181c
RBAK	ENSG00000146587	0,767192931	0,026480424	down	miR-181c
PARVB	ENSG00000188677	0,933573094	0,009572233	down	miR-181c
NGFR	ENSG00000064300	1,794103295	0,011639756	up	miR-181c
HIST1H3E	ENSG00000274750	2,113251721	0,024049233	up	miR-181c
KLHL12	ENSG00000117153	1,850639991	0,000950324	up	miR-181c
KRT86	ENSG00000170442	2,031906648	0,008678888	up	miR-181c
C16orf59	ENSG00000162062	1,133297042	0,036696775	up	miR-181c
N6AMT1	ENSG00000156239	3,135352006	4,89297E-06	up	miR-181c
SPRY4	ENSG00000187678	1,064719363	0,014941911	up	miR-181c
AC008105.1	ENSG00000233175	1,159237	0,014506648	up	miR-181c
GALNT18	ENSG00000110328	2,150593493	0,003572127	up	miR-181c

NABP1	ENSG00000173559	0,729993809	0,014822309	down	miR-181c
PAK6	ENSG00000137843	1,560978772	0,034469602	up	miR-181c
AC245884.8	ENSG00000267838	1,417927885	0,020915521	up	miR-181c
ASB16-AS1	ENSG00000267080	1,47370079	0,013590075	up	miR-181c
NUAK2	ENSG00000163545	1,915644711	0,016303748	up	miR-181c
PREX2	ENSG00000046889	1,650033045	0,002348074	up	miR-181c
PHOSPHO2	ENSG00000144362	1,173808251	0,033342765	up	miR-181c
HCFC2	ENSG00000111727	1,754181317	0,000136361	up	miR-181c
MDFIC	ENSG00000135272	1,522030297	0,002477971	up	miR-181c
NUP50	ENSG00000093000	1,536189049	0,000109255	up	miR-181c
LINC01671	ENSG00000225431	2,034024422	0,017996565	up	miR-181c
AC020915.3	ENSG00000268516	2,142281557	0,001252332	up	miR-181c
METRN	ENSG00000103260	1,182537195	0,025958301	up	miR-181c
ZNF92	ENSG00000146757	1,465954282	0,010617876	up	miR-181c
MDP1	ENSG00000213920	1,461254766	0,006775865	up	miR-181c
TNFRSF10B	ENSG00000120889	0,983724795	0,021484854	down	miR-181c
C9orf156	ENSG00000136932	1,590272586	0,000747791	up	miR-181c
RMND5A	ENSG00000153561	1,246724784	0,012438535	up	miR-181c
ZNF781	ENSG00000196381	1,646704636	0,002228179	up	miR-181c
DGCR5	ENSG00000237517	2,072556397	0,022247517	up	miR-181c
ISG20	ENSG00000172183	1,841753239	0,001689456	up	miR-181c
SNX33	ENSG00000173548	0,934487868	0,006522482	down	miR-181c
NRAV	ENSG00000248008	2,240405139	0,015293455	up	miR-181c
SNAI3	ENSG00000185669	1,299120116	0,01570163	up	miR-181c
HYLS1	ENSG00000198331	2,111608562	0,013455981	up	miR-181c
DRAM1	ENSG00000136048	1,017323859	0,02848422	up	miR-181c
NOB1	ENSG00000141101	1,145734168	0,009130755	up	miR-181c
ZNF837	ENSG00000152475	1,611495092	0,006125673	up	miR-181c
DNAL4	ENSG00000100246	1,943155329	0,000871214	up	miR-181c
FAM173A	ENSG00000103254	1,051038371	0,022489832	up	miR-181c
PLCB1	ENSG00000182621	2,018285272	0,000965616	up	miR-181c
AC107464.3	ENSG00000272927	2,313161025	0,00198097	up	miR-181c
RHOU	ENSG00000116574	1,417437898	0,003764219	up	miR-181c
RPUSD3	ENSG00000156990	1,354416533	0,007780699	up	miR-181c
TP53TG1	ENSG00000182165	1,100166344	0,027112182	up	miR-181c
AC027307.2	ENSG00000267317	1,402957805	0,024067711	up	miR-181c
USP32	ENSG00000170832	1,068163382	0,039125541	up	miR-181c
OXSM	ENSG00000151093	2,188987314	0,000108272	up	miR-181c
SUPV3L1	ENSG00000156502	0,987924844	0,011298021	down	miR-181c
TRAPPC4	ENSG00000196655	0,918410787	0,033574709	down	miR-181c
RBM12B	ENSG00000183808	0,80397881	0,020804803	down	miR-181c
THBD	ENSG00000178726	1,457969794	0,015770507	up	miR-181c
TMEM38B	ENSG00000095209	1,534948106	0,015869904	up	miR-181c
PRKAA2	ENSG00000162409	1,354213939	0,023400494	up	miR-181c
GALNT11	ENSG00000178234	0,717224687	0,042215311	down	miR-181c
ARHGEF17	ENSG00000110237	1,186430743	0,001157408	up	miR-181c
KLF13	ENSG00000169926	1,186339949	0,010130229	up	miR-181c
PNMAL1	ENSG00000182013	3,198008717	0,012338925	up	miR-181c
HACD4	ENSG00000188921	1,807701807	0,000859644	up	miR-181c
MRPL10	ENSG00000159111	0,819035894	0,024284198	down	miR-181c
CHST4	ENSG00000140835	4,308850737	0,018516166	up	miR-181c
CREB5	ENSG00000146592	1,447907493	0,001004646	up	miR-181c
GJA4	ENSG00000187513	1,923819305	8,95417E-05	up	miR-181c
RFX7	ENSG00000181827	0,935681398	0,049750812	down	miR-181c
COA1	ENSG00000106603	1,139610178	0,021610268	up	miR-181c
HEXIM2	ENSG00000168517	0,978420326	0,040991698	down	miR-181c
SREBF2	ENSG00000198911	0,896280288	0,009130299	down	miR-181c
PHC3	ENSG00000173889	1,118767651	2,48489E-05	up	miR-181c
C9orf106	ENSG00000179082	2,142576394	0,005897241	up	miR-181c
MTX2	ENSG00000128654	1,721045425	0,006678669	up	miR-181c
RAB23	ENSG00000112210	1,456864214	0,015168855	up	miR-181c
NEO1	ENSG00000067141	1,562782447	0,003595494	up	miR-181c
GEMIN4	ENSG00000179409	1,08555383	0,028269922	up	miR-181c
RGL1	ENSG00000143344	0,67234784	0,047700094	down	miR-181c
EVC2	ENSG00000173040	1,513627716	0,033493362	up	miR-181c
CCND2	ENSG00000118971	0,963410543	0,019795462	down	miR-181c
MIF4GD	ENSG00000125457	1,402838836	0,006319858	up	miR-181c
AL513329.1	ENSG00000227740	1,586413301	0,0191156	up	miR-181c
TMEM129	ENSG00000168936	1,170056104	0,005007301	up	miR-181c
POC1A	ENSG00000164087	1,643227333	0,011538509	up	miR-181c
PTAR1	ENSG00000188647	0,815870963	0,00563268	down	miR-181c
MAP2K1	ENSG00000169032	3,401726977	0,000221033	up	miR-181c
TGFBR3	ENSG00000069702	0,872929896	0,03868919	down	miR-181c
AIFM2	ENSG00000042286	1,563173902	0,001475557	up	miR-181c
MORF4L1	ENSG00000185787	1,014676374	0,034703547	up	miR-181c
CSTF2T	ENSG00000177613	1,210897373	0,011276023	up	miR-181c
TMEM42	ENSG00000169964	1,128485633	0,025655934	up	miR-181c

SVBP	ENSG00000177868	1,501089107	0,001252838	up	miR-181c
ITGBL1	ENSG00000198542	1,566970602	0,003412452	up	miR-181c
TSC22D3	ENSG00000157514	0,77227675	0,047346978	down	miR-181c
SCTR	ENSG00000080293	2,465271611	0,029702737	up	miR-181c
MYO1D	ENSG00000176658	1,303448009	0,016469671	up	miR-181c
SETD7	ENSG00000145391	0,598594209	0,030825604	down	miR-181c
JAG1	ENSG00000101384	0,880820406	0,028913744	down	miR-181c
CD8B	ENSG00000172116	1,715853855	0,017665717	up	miR-181c
HEG1	ENSG00000173706	0,993134253	0,013579433	down	miR-181c
KRBA1	ENSG00000133619	1,613862145	0,037148843	up	miR-181c
ADAMTS16	ENSG00000145536	2,628288721	0,038427568	up	miR-181c
HIPK3	ENSG00000110422	3,131293348	6,52496E-08	up	miR-181c
SURF4	ENSG00000148248	0,634558683	0,005796746	down	miR-181c
TDG	ENSG00000139372	0,985667676	0,00307206	down	miR-181c
KIAA1191	ENSG00000122203	1,525294613	0,000267805	up	miR-181c
C5orf45	ENSG00000161010	1,15826288	0,024408246	up	miR-181c
RPUSD2	ENSG00000166133	2,189815465	0,002168348	up	miR-181c
FAM174A	ENSG00000174132	1,993733441	0,002686274	up	miR-181c
ZNF823	ENSG00000197933	1,407120782	0,017428468	up	miR-181c
AGPS	ENSG00000018510	1,326368476	0,002620616	up	miR-181c
GZMK	ENSG00000113088	2,288369911	0,019898528	up	miR-181c
FAM50B	ENSG00000145945	2,016927207	0,003952831	up	miR-181c
KIAA1586	ENSG00000168116	1,533350661	0,001443363	up	miR-181c
VTCN1	ENSG00000134258	2,772931722	0,03011763	up	miR-181c
ITFG1	ENSG00000129636	0,639942648	0,041221996	down	miR-181c
ZNF529-AS1	ENSG00000233527	1,891904226	0,029483376	up	miR-181c
ZNF90	ENSG00000213988	1,457654773	0,009498982	up	miR-181c
ZKSCAN4	ENSG00000187626	2,090697324	0,00797692	up	miR-181c
SNU13	ENSG00000100138	0,75858761	0,000610824	down	miR-181c
TNFAIP8L3	ENSG00000183578	1,450911691	0,015757584	up	miR-181c
KLF7	ENSG00000118263	0,702819949	0,008888813	down	miR-181c
LOXL4	ENSG00000138131	1,84140893	0,034137394	up	miR-181c
TRAFD1	ENSG00000135148	1,024020603	0,044739877	up	miR-181c
ATP5H	ENSG00000167863	1,224451308	0,003822939	up	miR-181c
AMIGO1	ENSG00000181754	1,775084581	0,020311658	up	miR-181c
RNMTL1	ENSG00000171861	1,583919267	0,003359491	up	miR-181c
RAP2A	ENSG00000125249	1,173659866	0,037028851	up	miR-181c
LINC01238_1	ENSG00000237940	1,360430425	0,020599735	up	miR-181c
NTPCR	ENSG00000135778	1,64540089	0,001150449	up	miR-181c
HAAO	ENSG00000162882	1,769002913	0,008772395	up	miR-181c
WBP1	ENSG00000239779	1,202181175	0,021254925	up	miR-181c
PANK3	ENSG00000120137	0,87316538	0,006717475	down	miR-181c
ZNF625	ENSG00000257591	1,422406674	0,023046406	up	miR-181c
NIPSNAP1	ENSG00000184117	0,98295666	0,016557526	down	miR-181c
ZSCAN23	ENSG00000187987	2,130156184	0,018065846	up	miR-181c
KRT10	ENSG00000186395	1,59191813	0,005506047	up	miR-181c
NDUFAF3	ENSG00000178057	1,012976417	0,002945903	up	miR-181c
CPTP	ENSG00000224051	2,281608871	2,83014E-05	up	miR-181c
GMEB2	ENSG00000101216	1,110759316	0,019797142	up	miR-181c
C1GALT1C1	ENSG00000171155	2,245800124	0,000811043	up	miR-181c
MOSPD3	ENSG00000106330	1,080318621	0,009601163	up	miR-181c
SDF2	ENSG00000132581	1,169743538	0,000421476	up	miR-181c
PPAN	ENSG00000130810	0,913345912	0,002867285	down	miR-181c
GPN3	ENSG00000111231	1,98593854	0,00854973	up	miR-181c
FIP1L1	ENSG00000145216	0,59569442	0,019977232	down	miR-181c
PRDX3	ENSG00000165672	1,454384524	0,004136898	up	miR-181c
ANP32A	ENSG00000140350	1,023105319	0,001211677	up	miR-181c
TNFSF14	ENSG00000125735	1,660378764	0,023587281	up	miR-181c
CBX2	ENSG00000173894	2,005935515	0,040858075	up	miR-181c
AC006213.2	ENSG00000267058	1,494934185	0,03967533	up	miR-181c
NID2	ENSG00000087303	1,403564355	0,006519141	up	miR-181c
TCAF2	ENSG00000170379	2,224649326	0,007264653	up	miR-181c
PPP1R14A	ENSG00000167641	1,551714132	0,008415868	up	miR-181c
ZNF449	ENSG00000173275	1,652764197	0,001072457	up	miR-181c
SMDT1	ENSG00000183172	1,056566275	0,004748055	up	miR-181c
LCORL	ENSG00000178177	0,972340019	0,036221838	down	miR-181c
ECI1	ENSG00000167969	0,882119445	0,030619824	down	miR-181c
FBXO28	ENSG00000143756	1,377605996	0,018902901	up	miR-181c
LRRC47	ENSG00000130764	1,20924009	0,000808423	up	miR-181c
HBP1	ENSG00000105856	0,906532347	0,00113319	down	miR-181c
TMEM121	ENSG00000184986	2,36651558	0,008892667	up	miR-181c
SEC61B	ENSG00000106803	0,839136227	0,039927481	down	miR-181c
EPHX3	ENSG00000105131	1,425928323	0,01344715	up	miR-181c
NDUFB9	ENSG00000147684	1,008650228	0,003502431	up	miR-181c
RBM4	ENSG00000173933	1,021505596	0,02978876	up	miR-181c
PLD1	ENSG00000075651	0,912433276	0,027074577	down	miR-181c
SSC5D	ENSG00000179954	0,891904764	0,019523918	down	miR-181c

UPRT	ENSG00000094841	2,257047529	0,000439054	up	miR-181c
ST7-AS1	ENSG00000227199	0,768049753	0,021191718	down	miR-181c
GNB2	ENSG00000172354	0,9626323	0,017450814	down	miR-181c
NUDT2	ENSG00000164978	1,384616258	0,001314697	up	miR-181c
UNC5B	ENSG00000107731	1,560072656	0,000268142	up	miR-181c
ANKRD54	ENSG00000100124	1,146284509	0,012649272	up	miR-181c
NDUFA4	ENSG00000189043	0,75125458	0,012035374	down	miR-181c
ASTN2	ENSG00000148219	1,255174779	0,011914147	up	miR-181c
KCMF1	ENSG00000176407	0,634901404	0,045922933	down	miR-181c
HRSP12	ENSG00000132541	1,3638828	0,034733479	up	miR-181c
TRIB3	ENSG00000101255	1,648253946	0,031562051	up	miR-181c
REPIN1	ENSG00000214022	0,856841826	0,027443549	down	miR-181c
CHRNB1	ENSG00000170175	2,235855118	0,005441042	up	miR-181c
MON2	ENSG00000061987	0,778303252	0,01739367	down	miR-181c
HGD	ENSG00000113924	1,518575656	0,030979013	up	miR-181c
RANGRF	ENSG00000108961	1,587981329	0,002253841	up	miR-181c
ATG4C	ENSG00000125703	1,990618815	0,000334497	up	miR-181c
TMEM127	ENSG00000135956	0,959305218	0,000719845	down	miR-181c
C5orf24	ENSG00000181904	0,795201912	0,016747098	down	miR-181c
PXDC1	ENSG00000168994	1,255207522	0,022129114	up	miR-181c
TEX9	ENSG00000151575	2,02714384	0,003200002	up	miR-181c
CHMP5	ENSG00000086065	1,948965082	4,02856E-05	up	miR-181c
C11orf73	ENSG00000149196	0,927979972	0,024532117	down	miR-181c
F2R	ENSG00000181104	1,513759228	0,002533531	up	miR-181c
SNRK	ENSG00000163788	1,272406341	0,000482098	up	miR-181c
IVD	ENSG00000128928	0,736171024	0,033492708	down	miR-181c
SMARCAD1	ENSG00000163104	0,887433988	0,006487204	down	miR-181c
GUCY1A3	ENSG00000164116	1,069308896	0,016577155	up	miR-181c
FECH	ENSG00000066926	1,515139532	0,010489396	up	miR-181c
PAFAH1B1	ENSG00000007168	0,727842038	0,027881243	down	miR-181c
C19orf38	ENSG00000214212	1,342341999	0,036128611	up	miR-181c
SHISA9	ENSG00000237515	3,879327112	0,017591057	up	miR-181c
WASF3	ENSG00000132970	1,631334232	0,008061677	up	miR-181c
ODF3B	ENSG00000177989	2,223919278	0,000296047	up	miR-181c
SCAMP1	ENSG00000085365	0,848468321	0,021716094	down	miR-181c
CENPP	ENSG00000188312	2,129630561	0,001711099	up	miR-181c
SZRD1	ENSG00000055070	0,835791649	0,042522704	down	miR-181c
UGDH	ENSG00000109814	1,230773774	0,000779307	up	miR-181c
SEC24A	ENSG00000113615	1,146060801	0,025368581	up	miR-181c
ADPRHL2	ENSG00000116863	1,655436576	0,002667414	up	miR-181c
SMG7	ENSG00000116698	0,697728787	0,035271952	down	miR-181c
CCR2	ENSG00000121807	2,789341113	0,002918921	up	miR-181c
XBP1	ENSG00000100219	1,011698523	0,016438842	up	miR-181c
PITPNB	ENSG00000180957	0,677298559	0,026681174	down	miR-181c
FOXJ3	ENSG00000198815	1,183846092	0,000740444	up	miR-181c
SPG21	ENSG00000090487	1,079668104	0,010430568	up	miR-181c
ITGB2	ENSG00000160255	1,226805021	0,003553102	up	miR-181c
GLIS3	ENSG00000107249	0,782304383	0,001013325	down	miR-181c
NDUFA7	ENSG00000267855	2,509032876	1,33206E-05	up	miR-181c
SCNM1	ENSG00000163156	0,783959167	0,02277139	down	miR-181c
SNRPB2	ENSG00000125870	0,918814402	0,017915011	down	miR-181c
NSUN6	ENSG00000241058	0,862893463	0,038837003	down	miR-181c
CUL4A	ENSG00000139842	0,673885911	0,03449122	down	miR-181c
DOK1	ENSG00000115325	1,804276237	0,002699005	up	miR-181c
IFNAR1	ENSG00000142166	0,590236013	0,026773906	down	miR-181c
RPL39L	ENSG00000163923	1,602685197	0,022578942	up	miR-181c
TBC1D2	ENSG00000095383	2,721136071	0,016895377	up	miR-181c
IL1RAP	ENSG00000196083	1,427273441	0,006032225	up	miR-181c
RPSA	ENSG00000168028	0,54747837	0,048416432	down	miR-181c
SRPRB	ENSG00000144867	0,959784349	0,002954075	down	miR-181c
SESN2	ENSG00000130766	0,962803286	0,008928025	down	miR-181c
NRAS	ENSG00000213281	0,981420506	0,014752491	down	miR-181c
NDUFC2	ENSG00000151366	0,76573168	0,011703622	down	miR-181c
ATAD1	ENSG00000138138	0,78902149	0,016364792	down	miR-181c
OSTC	ENSG00000198856	1,696527052	4,59981E-05	up	miR-181c
TMTC3	ENSG00000139324	0,80692316	0,022467133	down	miR-181c
ARPC5	ENSG00000162704	0,523810999	0,024904889	down	miR-181c
PPP2R2C	ENSG00000074211	3,173328458	0,003450038	up	miR-181c
ANO8	ENSG00000074855	1,463941015	0,032910401	up	miR-181c
GOLPH3	ENSG00000113384	1,473470913	0,001810712	up	miR-181c
OSCP1	ENSG00000116885	1,759418242	0,023320979	up	miR-181c
RAB1A	ENSG00000138069	0,874558075	0,015392894	down	miR-181c
HACD3	ENSG00000074696	0,82764358	0,003662778	down	miR-181c
AJUBA	ENSG00000129474	1,063676047	0,048937671	up	miR-181c
UBE2J1	ENSG00000198833	0,760376097	0,004535213	down	miR-181c
PCBD2	ENSG00000132570	1,381194233	0,006396676	up	miR-181c
AL355488.1	ENSG00000273373	2,641013685	2,46866E-05	up	miR-181c

TINF2	ENSG00000092330	1,319666569	0,004722751	up	miR-181c
TXNDC11	ENSG00000153066	2,050116128	4,27834E-05	up	miR-181c
SHB	ENSG00000107338	1,130657763	0,016715611	up	miR-181c
RPE	ENSG00000197713	1,063651841	0,010769312	up	miR-181c
GAMT	ENSG00000130005	2,207617734	0,00506754	up	miR-181c
NDFIP1	ENSG00000131507	0,656482861	0,013329699	down	miR-181c
ADO	ENSG00000181915	1,628454138	0,000441174	up	miR-181c
ZNF768	ENSG00000169957	0,929388515	0,02326451	down	miR-181c
CBX4	ENSG00000141582	0,920025547	0,005047114	down	miR-181c
EDA2R	ENSG00000131080	2,325697696	0,001664498	up	miR-181c
LNPEP	ENSG00000113441	0,70282519	0,002819633	down	miR-181c
KIAA1429	ENSG00000164944	0,671813537	0,011800581	down	miR-181c
ADNP	ENSG00000101126	0,910974018	0,001475451	down	miR-181c
DHX9	ENSG00000135829	0,988246126	0,006707488	down	miR-181c
CLYBL	ENSG00000125246	1,591660154	0,034379595	up	miR-181c
FBXO18	ENSG00000134452	0,679611732	0,016842718	down	miR-181c
CFI	ENSG00000205403	0,990766081	0,020004761	down	miR-181c
MRPL34	ENSG00000130312	3,073815687	0,000534828	up	miR-181c
PARL	ENSG00000175193	0,782719532	0,009080289	down	miR-181c
C4orf19	ENSG00000154274	2,121703039	0,00354884	up	miR-181c
AFG3L2	ENSG00000141385	1,059019427	0,001600875	up	miR-181c
ACLY	ENSG00000131473	0,528717584	0,029826637	down	miR-181c
TMEM109	ENSG00000110108	0,974787747	0,001964439	down	miR-181c
PLK2	ENSG00000145632	1,017915238	0,016914319	up	miR-181c
ZNF598	ENSG00000167962	0,729889086	0,039270164	down	miR-181c
METTL2B	ENSG00000165055	0,948787841	0,006116266	down	miR-181c
TMEM140	ENSG00000146859	1,147966125	0,020802065	up	miR-181c
PSMD13	ENSG00000185627	0,859787612	0,00385757	down	miR-181c
ETF1	ENSG00000120705	0,638582022	0,0237397	down	miR-181c
AC010503.4	ENSG00000275234	1,645735274	0,013423401	up	miR-181c
BCL2	ENSG00000171791	1,020729308	0,012914207	up	miR-181c
PPP2CB	ENSG00000104695	0,67911617	0,024745159	down	miR-181c
EPT1	ENSG00000138018	1,195541798	0,004931779	up	miR-181c
FIBIN	ENSG00000176971	1,651179778	0,001326116	up	miR-181c
IFNGR1	ENSG00000027697	1,458078434	0,006014319	up	miR-181c
FAAH2	ENSG00000165591	2,086137556	0,00858262	up	miR-181c
AGL	ENSG00000162688	1,316883579	0,000963464	up	miR-181c
SNRPD1	ENSG00000167088	0,587696941	0,029163814	down	miR-181c
RABEP1	ENSG00000029725	0,693360526	0,006359637	down	miR-181c
GSK3B	ENSG00000082701	0,49606785	0,034041915	down	miR-181c
RPL17	ENSG00000265681	1,305729029	0,036413273	up	miR-181c
SNRPD3	ENSG00000100028	0,454341146	0,028635136	down	miR-181c
GLT8D1	ENSG00000016864	0,978800829	0,003295694	down	miR-181c
VPS33B	ENSG00000184056	1,256377044	0,046728585	up	miR-181c
PRPF19	ENSG00000110107	0,478305678	0,044858268	down	miR-181c
BAX	ENSG00000087088	0,843536525	0,006161342	down	miR-181c
CREG1	ENSG00000143162	0,820450699	0,012145292	down	miR-181c
LYPLA1	ENSG00000120992	1,182384782	0,003478491	up	miR-181c
MRO	ENSG00000134042	0,485385533	0,036846645	down	miR-181c
NAA25	ENSG00000111300	0,880768368	0,01489335	down	miR-181c
TUSC1	ENSG00000198680	1,360090728	0,034280498	up	miR-181c
HIST1H4E	ENSG00000276966	2,57838533	0,044399456	up	miR-181c
LTN1	ENSG00000198862	0,659688813	0,019243598	down	miR-181c
OSBPL8	ENSG00000091039	1,70277565	0,015381729	up	miR-181c
RRP1B	ENSG00000160208	0,635328382	0,048207254	down	miR-181c
ICE1	ENSG00000164151	0,713087425	0,023333735	down	miR-181c
LPGAT1	ENSG00000123684	0,795283051	0,010917054	down	miR-181c
AKR1B10	ENSG00000198074	1,835752017	0,018288725	up	miR-181c
C18orf32	ENSG00000177576	1,280766841	0,01380719	up	miR-181c
RPS7	ENSG00000171863	0,854483576	0,034589716	down	miR-181c
GNL2	ENSG00000134697	1,00907688	0,031918064	up	miR-181c
NAT6	ENSG00000243477	2,051652304	0,001029632	up	miR-181c
FBXW2	ENSG00000119402	0,738326911	0,003616096	down	miR-181c
CDKN2AIP	ENSG00000168564	0,930450607	0,004110783	down	miR-181c
LDLRAD3	ENSG00000179241	1,158146116	0,014368757	up	miR-181c
RFNG	ENSG00000169733	0,686942508	0,031247272	down	miR-181c
ELAVL1	ENSG00000066044	0,786577567	0,009965691	down	miR-181c
NOL6	ENSG00000165271	1,084696944	0,02569426	up	miR-181c
CAMSAP2	ENSG00000118200	1,067630954	0,044255394	up	miR-181c
METTL13	ENSG00000010165	1,481088364	0,005796174	up	miR-181c
RPS6KC1	ENSG00000136643	1,649853199	0,000359257	up	miR-181c
TSTD1	ENSG00000215845	0,947422476	0,001020247	down	miR-181c
SPOPL	ENSG00000144228	0,896104537	0,048247358	down	miR-181c
TMEM9B	ENSG00000175348	0,98595838	0,009491759	down	miR-181c
INPP5E	ENSG00000148384	1,382716264	0,046601864	up	miR-181c
AC106869.1	ENSG00000234690	1,423763744	0,025066551	up	miR-181c
ZNF839	ENSG00000022976	0,818664834	0,03688849	down	miR-181c

SLC30A9	ENSG00000014824	0,821448813	0,006098096	down	miR-181c
GSK3A	ENSG00000105723	1,376598265	0,000281762	up	miR-181c
MRPL24	ENSG00000143314	1,201479536	0,002580482	up	miR-181c
PCNP	ENSG00000081154	0,648681744	0,008516944	down	miR-181c
CHMP2A	ENSG00000130724	0,604354258	0,029861508	down	miR-181c
PSMB2	ENSG00000126067	0,6298803	0,00220477	down	miR-181c
RASSF8	ENSG00000123094	0,757497776	0,003819053	down	miR-181c
TMEM230	ENSG00000089063	1,335292323	0,020343259	up	miR-181c
SIRPA	ENSG00000198053	1,060563031	0,010301445	up	miR-181c
EPS15	ENSG00000085832	0,667726483	0,007095458	down	miR-181c
FZD5	ENSG00000163251	1,55911023	0,006557651	up	miR-181c
MTF1	ENSG00000188786	1,031479851	0,014005515	up	miR-181c
CSNK1G3	ENSG00000151292	0,794967401	0,01599736	down	miR-181c
KLHL9	ENSG00000198642	1,028164494	0,01033395	up	miR-181c
PIKFYVE	ENSG00000115020	0,618682531	0,014531387	down	miR-181c
PUM2	ENSG00000055917	0,784558833	0,007734495	down	miR-181c
ACMSD	ENSG00000153086	2,754294931	0,008454669	up	miR-181c
MBNL3	ENSG00000076770	2,123028988	0,009518915	up	miR-181c
GNPTG	ENSG00000090581	0,863182921	0,003436803	down	miR-181c
RRP15	ENSG00000067533	0,745152817	0,026875528	down	miR-181c
ATMIN	ENSG00000166454	0,699900616	0,041764849	down	miR-181c
DENR	ENSG00000139726	0,645190468	0,013035565	down	miR-181c
WWC1	ENSG00000113645	0,904936603	0,006634873	down	miR-181c
PON1	ENSG00000005421	4,25991881	0,016463118	up	miR-181c
DIABLO	ENSG00000184047	1,238359607	0,003819991	up	miR-181c
URI1	ENSG00000105176	0,757111973	0,01825245	down	miR-181c
MRPL11	ENSG00000174547	1,340829165	0,000504976	up	miR-181c
ASCC1	ENSG00000138303	1,249804487	0,014549026	up	miR-181c
CKB	ENSG00000166165	1,136884032	0,016379885	up	miR-181c
SNRNP27	ENSG00000124380	2,195126156	0,000448564	up	miR-181c
TMA16	ENSG00000198498	0,816121448	0,008748163	down	miR-181c
KCNJ11	ENSG00000187486	2,15328337	0,014434046	up	miR-181c
MED27	ENSG00000160563	0,82763762	0,011928093	down	miR-181c
MRPS18C	ENSG00000163319	1,523374265	0,000136432	up	miR-181c
KLHL8	ENSG00000145332	0,721883789	0,034388655	down	miR-181c
CLSTN2	ENSG00000158258	1,863322872	0,000362078	up	miR-181c
ANAPC11	ENSG00000141552	1,157094931	0,016764512	up	miR-181c
RAD54B	ENSG00000197275	1,17980347	0,008298324	up	miR-181c
LINC02027	ENSG00000243694	1,140810996	0,011112111	up	miR-181c
USP1	ENSG00000162607	1,119053712	0,000259297	up	miR-181c
TMEM59	ENSG00000116209	0,838446191	0,000959525	down	miR-181c
SH3YL1	ENSG00000035115	1,003327322	0,001632616	up	miR-181c
BICD2	ENSG00000185963	0,725166622	0,024117609	down	miR-181c
KLHDC3	ENSG00000124702	0,892455076	0,004871339	down	miR-181c
HKDC1	ENSG00000156510	1,398468692	0,008457857	up	miR-181c
EPN2	ENSG00000072134	0,628532614	0,026399901	down	miR-181c
XPO7	ENSG00000130227	0,828963089	0,004903676	down	miR-181c
ZDHHC7	ENSG00000153786	1,075420971	0,003282486	up	miR-181c
ULK1	ENSG00000177169	0,750253181	0,049958178	down	miR-181c
CX3CR1	ENSG00000168329	1,303259091	0,032545921	up	miR-181c
ATP5C1	ENSG00000165629	0,912526421	0,028056434	down	miR-181c
IGSF8	ENSG00000162729	2,584644921	6,04912E-05	up	miR-181c
TIGD1	ENSG00000221944	1,596512477	0,030538728	up	miR-181c
DSE	ENSG00000111817	0,776221505	0,018921152	down	miR-181c
ILF2	ENSG00000143621	0,833395953	0,035563722	down	miR-181c
CAPRIN1	ENSG00000135387	0,657669553	0,011965143	down	miR-181c
AIP	ENSG00000110711	1,137202508	0,025452314	up	miR-181c
BMS1	ENSG00000165733	1,725357216	0,000112929	up	miR-181c
COX14	ENSG00000178449	0,891458329	0,031025656	down	miR-181c
TMEM11	ENSG00000178307	0,915999598	0,010296299	down	miR-181c
RBBP8	ENSG00000101773	0,718594962	0,017927593	down	miR-181c
OTUD5	ENSG00000068308	0,340254483	0,032214161	down	miR-181c
MMP2	ENSG00000087245	1,45889872	0,0209354	up	miR-181c
TOP2B	ENSG00000077097	0,817557805	0,006390374	down	miR-181c
LRRC58	ENSG00000163428	0,736933617	0,008816901	down	miR-181c
CLPTM1L	ENSG00000049656	0,658922387	0,004039268	down	miR-181c
EPB41L4A-AS1	ENSG00000224032	0,880323977	0,03712194	down	miR-181c
EIF1AX	ENSG00000173674	0,846535863	0,002251485	down	miR-181c
HOOK1	ENSG00000134709	1,119813546	0,01267395	up	miR-181c
FHOD1	ENSG00000135723	1,450158748	0,004601641	up	miR-181c
CDK4	ENSG00000135446	0,645018455	0,016174184	down	miR-181c
TAF1	ENSG00000147133	1,002994731	0,0051369	up	miR-181c
AC007541.1	ENSG00000260329	1,749951095	0,009342662	up	miR-181c
TXNDC17	ENSG00000129235	1,043926732	0,001892767	up	miR-181c
NUDC	ENSG00000090273	1,04959518	0,000443882	up	miR-181c
SNRPC	ENSG00000124562	0,476154125	0,046313441	down	miR-181c
C19orf53	ENSG00000104979	1,248947354	0,001929881	up	miR-181c

LRRC49	ENSG00000137821	1,614962023	0,0069544	up	miR-181c
HOMER3	ENSG00000051128	0,804595578	0,019942754	down	miR-181c
MYDGF	ENSG00000074842	0,730974501	0,027521025	down	miR-181c
TMEM14B	ENSG00000137210	1,99204885	0,000879297	up	miR-181c
USP3	ENSG00000140455	0,735153516	0,01350979	down	miR-181c
FAM172A	ENSG00000113391	0,56039455	0,042547437	down	miR-181c
MRPS21	ENSG00000266472	0,974601554	0,006617802	down	miR-181c
HNMT	ENSG00000150540	1,120940105	0,008893781	up	miR-181c
PTGDR	ENSG00000168229	1,547803354	0,019791501	up	miR-181c
EXOSC6	ENSG00000223496	0,803674712	0,010729112	down	miR-181c
PFKFB3	ENSG00000170525	1,296723443	0,000834925	up	miR-181c
TSPAN12	ENSG00000106025	1,483879363	0,018685314	up	miR-181c
AC018797.2	ENSG00000246560	1,21584706	0,038680244	up	miR-181c
FASTKD2	ENSG00000118246	0,920224024	0,042596711	down	miR-181c
NT5C3B	ENSG00000141698	1,095028063	0,019464702	up	miR-181c
POLR2G	ENSG00000168002	0,83403239	0,008371832	down	miR-181c
PHC2	ENSG00000134686	0,925073522	0,005020636	down	miR-181c
ZBTB10	ENSG00000205189	1,042442966	0,011008071	up	miR-181c
NFX1	ENSG00000086102	0,755816951	0,026260437	down	miR-181c
NECAP2	ENSG00000157191	1,35208594	0,005537784	up	miR-181c
LARP4	ENSG00000161813	0,79435085	0,018557562	down	miR-181c
FBXO7	ENSG00000100225	0,945544583	0,008188791	down	miR-181c
ACAA1	ENSG00000060971	0,751586219	0,042201201	down	miR-181c
CDH24	ENSG00000139880	2,154707502	0,010327053	up	miR-181c
CWC22	ENSG00000163510	0,793567961	0,003945489	down	miR-181c
MAGOHB	ENSG00000111196	0,976575234	0,025136952	down	miR-181c
AHCTF1	ENSG00000153207	0,655890443	0,047589947	down	miR-181c
SLC22A17	ENSG00000092096	1,850408584	0,002065417	up	miR-181c
UBE2B	ENSG00000119048	0,801728955	0,008486589	down	miR-181c
PPP1R8	ENSG00000117751	0,680290962	0,038406801	down	miR-181c
IDH1	ENSG00000138413	0,704847606	0,023886116	down	miR-181c
TOMM70A	ENSG00000154174	0,825589339	0,010911693	down	miR-181c
SEPHS1	ENSG00000086475	0,754193017	0,014469953	down	miR-181c
VDAC1	ENSG00000213585	0,881727708	0,009713735	down	miR-181c
UCHL5	ENSG00000116750	0,690645422	0,032211507	down	miR-181c
RAB2B	ENSG00000129472	1,726003943	0,000158493	up	miR-181c
GAPVD1	ENSG00000165219	1,226379749	0,000669459	up	miR-181c
PARP6	ENSG00000137817	0,818550125	0,003153265	down	miR-181c
STK24	ENSG00000102572	1,003126492	0,004461142	up	miR-181c
RALGAPB	ENSG00000170471	0,626550966	0,020746403	down	miR-181c
GNE	ENSG00000159921	1,496507272	0,016306374	up	miR-181c
ASCC2	ENSG00000100325	1,035045112	0,006116869	up	miR-181c
CTDNEP1	ENSG00000175826	0,609564415	0,021905337	down	miR-181c
ZNF772	ENSG00000197128	1,50212768	0,005867924	up	miR-181c
ARPP19	ENSG00000128989	1,132871926	0,002252486	up	miR-181c
RANBP2	ENSG00000153201	0,559933957	0,004000137	down	miR-181c
TOR1AIP1	ENSG00000143337	0,767086215	0,02742346	down	miR-181c
STRBP	ENSG00000165209	0,802277805	0,0494053	down	miR-181c
TCTN2	ENSG00000168778	1,517973771	0,008773882	up	miR-181c
ST14	ENSG00000149418	0,713768707	0,028728654	down	miR-181c
TTC38	ENSG00000075234	0,861403828	0,021470399	down	miR-181c
STK33	ENSG00000130413	2,450424294	0,004529161	up	miR-181c
FAM8A1	ENSG00000137414	1,215326965	0,002507775	up	miR-181c
YPEL5	ENSG00000119801	0,999737714	0,000972471	down	miR-181c
KIF16B	ENSG00000089177	0,645930813	0,029850974	down	miR-181c
MBD2	ENSG00000134046	0,76154472	0,003701221	down	miR-181c
MSRB3	ENSG00000174099	1,70103013	0,000753006	up	miR-181c
PYGO2	ENSG00000163348	1,6402251	1,55262E-05	up	miR-181c
HNF4A	ENSG00000101076	2,348040544	0,000733927	up	miR-181c
UBE2Q1	ENSG00000160714	0,684245325	0,006181952	down	miR-181c
FNIP1	ENSG00000217128	0,892432698	0,008007742	down	miR-181c
MIB1	ENSG00000101752	0,766006626	0,001004619	down	miR-181c
ZFAS1	ENSG00000177410	0,47229587	0,029009464	down	miR-181c
CCSER2	ENSG00000107771	0,745703178	0,01541347	down	miR-181c
PIP4K2A	ENSG00000150867	0,783456663	0,027715418	down	miR-181c
EBLN3P	ENSG00000281649	0,981134056	0,000461896	down	miR-181c
RAB29	ENSG00000117280	1,004920549	0,002865154	up	miR-181c
CNN3	ENSG00000117519	2,060439847	6,25434E-05	up	miR-181c
CEP70	ENSG00000114107	0,861283726	0,040573731	down	miR-181c
WDR31	ENSG00000148225	1,170856761	0,038300963	up	miR-181c
PAK4	ENSG00000130669	0,657365944	0,014669425	down	miR-181c
CFTR	ENSG00000001626	1,360754609	0,035502567	up	miR-181c
ASF1A	ENSG00000111875	0,802465141	0,031393201	down	miR-181c
SNRPN	ENSG00000128739	1,61300959	0,001862279	up	miR-181c
NDUFB8	ENSG00000166136	0,732234407	0,016689383	down	miR-181c
SFXN1	ENSG00000164466	0,515594824	0,048024146	down	miR-181c
ABI1	ENSG00000136754	0,81616014	0,0168999	down	miR-181c

ACTR1B	ENSG00000115073	0,453836227	0,003397216	down	miR-181c
AEN	ENSG00000181026	1,374251073	0,000518756	up	miR-181c
DUSP22	ENSG00000112679	0,866307146	0,002200995	down	miR-181c
MRPL43	ENSG00000055950	0,892355535	0,014630281	down	miR-181c
IDH2	ENSG00000182054	0,763841949	0,020598934	down	miR-181c
ATP6V1E1	ENSG00000131100	1,047834874	0,001014633	up	miR-181c
SLC37A4	ENSG00000137700	1,117277538	0,012726585	up	miR-181c
SF3A1	ENSG00000099995	0,602466812	0,031053437	down	miR-181c
TMEM106C	ENSG00000134291	1,052531136	0,010412875	up	miR-181c
RGN	ENSG00000130988	2,012696952	0,030660077	up	miR-181c
ALKBH5	ENSG00000091542	0,700029688	0,043473299	down	miR-181c
BORCS7	ENSG00000166275	1,099348435	0,013138438	up	miR-181c
ACP5	ENSG00000102575	1,412063899	0,017922252	up	miR-181c
RAB8A	ENSG00000167461	0,578660124	0,015286229	down	miR-181c
TMEM62	ENSG00000137842	1,59322925	0,001534675	up	miR-181c
TRADD	ENSG00000102871	0,637931189	0,048750181	down	miR-181c
VEZF1	ENSG00000136451	0,692674956	0,008011486	down	miR-181c
ESYT1	ENSG00000139641	0,783829384	0,01914778	down	miR-181c
FRG1	ENSG00000109536	0,648415258	0,010608907	down	miR-181c
TJP1	ENSG00000104067	0,810822892	0,035348758	down	miR-181c
IFFO1	ENSG00000010295	1,095182586	0,003920833	up	miR-181c
LRRC42	ENSG00000116212	2,060010144	0,000150181	up	miR-181c
SDCBP2-AS1	ENSG00000234684	1,602406123	0,00350947	up	miR-181c
LRRC41	ENSG00000132128	1,0977108	0,015190402	up	miR-181c
TEX2	ENSG00000136478	0,843444728	0,036670734	down	miR-181c
PLSCR1	ENSG00000188313	1,314231357	0,002284515	up	miR-181c
HTRA2	ENSG00000115317	1,251340727	0,002038029	up	miR-181c
FBXL5	ENSG00000118564	0,652040045	0,005767755	down	miR-181c
CDC42	ENSG00000070831	1,181320611	0,000681978	up	miR-181c
COA6	ENSG00000168275	1,743070629	0,012059945	up	miR-181c
COPS2	ENSG00000166200	0,583435327	0,012236692	down	miR-181c
AP3S1	ENSG00000177879	0,786059245	0,038650354	down	miR-181c
VTI1B	ENSG00000100568	0,723262644	0,027364431	down	miR-181c
RCOR3	ENSG00000117625	0,939818824	0,001676828	down	miR-181c
EIF3H	ENSG00000147677	0,808612491	0,002528339	down	miR-181c
STARD7	ENSG00000084090	0,642208117	0,03613028	down	miR-181c
FAM98B	ENSG00000171262	1,301247117	0,000875717	up	miR-181c
DGUOK	ENSG00000114956	1,351647595	0,001288792	up	miR-181c
G3BP2	ENSG00000138757	0,64590111	0,008609108	down	miR-181c
FCGR3A	ENSG00000203747	0,658995061	0,034159605	down	miR-181c
ZNF280D	ENSG00000137871	0,631420196	0,00773141	down	miR-181c
TMEM248	ENSG00000106609	0,700177461	0,037635578	down	miR-181c
GBAS	ENSG00000146729	0,890595856	0,027530748	down	miR-181c
PIK3CA	ENSG00000121879	0,30003865	0,039702114	down	miR-181c
AAMP	ENSG00000127837	0,868630532	0,012159337	down	miR-181c
UBQLN1	ENSG00000135018	1,174104286	9,67416E-05	up	miR-181c
IFT46	ENSG00000118096	1,12804399	0,011072615	up	miR-181c
CENPC	ENSG00000145241	0,778791093	0,023744223	down	miR-181c
IER3IP1	ENSG00000134049	1,394841037	0,001549178	up	miR-181c
TMEM245	ENSG00000106771	0,427971824	0,013395647	down	miR-181c
RNF181	ENSG00000168894	0,830109428	0,009218751	down	miR-181c
NOP10	ENSG00000182117	1,291865675	0,02008773	up	miR-181c
FKBP3	ENSG00000100442	0,572405652	0,013792409	down	miR-181c
TPP2	ENSG00000134900	0,725914569	0,000597105	down	miR-181c
ATP11C	ENSG00000101974	0,573063245	0,005881963	down	miR-181c
SLC25A36	ENSG00000114120	0,47654089	0,029718191	down	miR-181c
CBR1	ENSG00000159228	1,33474373	0,002131245	up	miR-181c
FH	ENSG00000091483	1,513643765	0,002923517	up	miR-181c
KANK3	ENSG00000186994	1,315000665	0,009504494	up	miR-181c
SNAP25	ENSG00000132639	2,630025676	0,011592185	up	miR-181c
SMIM6	ENSG00000259120	1,430528933	0,034195444	up	miR-181c
TRIM26	ENSG00000234127	1,154532158	0,003241088	up	miR-181c
PSMB4	ENSG00000159377	1,116788619	0,00476923	up	miR-181c
ATL3	ENSG00000184743	0,768023333	0,021836281	down	miR-181c
BUB3	ENSG00000154473	1,331190663	0,000106834	up	miR-181c
NDUFAB1	ENSG00000004779	0,880738298	0,002667513	down	miR-181c
PSMF1	ENSG00000125818	0,976049114	0,003714966	down	miR-181c
C11orf68	ENSG00000175573	0,342234128	0,016850458	down	miR-181c
PLIN2	ENSG00000147872	1,047744825	0,009106048	up	miR-181c
EBNA1BP2	ENSG00000117395	0,960836079	0,006863005	down	miR-181c
LAD1	ENSG00000159166	1,318297206	0,003539023	up	miR-181c
CCDC43	ENSG00000180329	0,605693578	0,029183786	down	miR-181c
RABL2A	ENSG00000144134	1,240027797	0,022573884	up	miR-181c
SLC35F6	ENSG00000213699	1,081167887	0,000823176	up	miR-181c
TBC1D2B	ENSG00000167202	0,750026059	0,014724522	down	miR-181c
ASXL2	ENSG00000143970	0,698227491	0,011024039	down	miR-181c
MMP7	ENSG00000137673	1,583824219	0,014078636	up	miR-181c

CSNK1D	ENSG00000141551	0,565102235	0,033295793	down	miR-181c
CLK1	ENSG00000013441	1,222228737	0,000301173	up	miR-181c
VAPB	ENSG00000124164	0,753586912	0,012696428	down	miR-181c
SGPL1	ENSG00000166224	0,545714139	0,024453163	down	miR-181c
UPF1	ENSG00000005007	1,111408204	0,00251538	up	miR-181c
SOS1	ENSG00000115904	0,649147123	0,011718157	down	miR-181c
ASPN	ENSG00000106819	0,742331394	0,035066871	down	miR-181c
ADI1	ENSG00000182551	0,879472628	0,022366423	down	miR-181c
ETFA	ENSG00000140374	0,895120157	0,019833562	down	miR-181c
RYK	ENSG00000163785	0,625907154	0,007019859	down	miR-181c
THAP11	ENSG00000168286	1,464644877	0,010897298	up	miR-181c
CPPED1	ENSG00000103381	1,427820775	0,0006914	up	miR-181c
MZT2A	ENSG00000173272	0,718727685	0,033163202	down	miR-181c
DNAJC13	ENSG00000138246	0,938263753	0,010296376	down	miR-181c
CCDC58	ENSG00000160124	2,694300452	3,49834E-07	up	miR-181c
TEFM	ENSG00000172171	1,654295996	0,002099501	up	miR-181c
TRPM7	ENSG00000092439	1,423717132	0,006124118	up	miR-181c
ZNF770	ENSG00000198146	0,624318052	0,007833197	down	miR-181c
ZSWIM8	ENSG00000214655	0,608849385	0,011650882	down	miR-181c
PMFBP1	ENSG00000118557	1,183278784	0,031072139	up	miR-181c
SECISBP2	ENSG00000187742	0,875226202	0,006543175	down	miR-181c
EMC4	ENSG00000128463	1,615648559	0,000127137	up	miR-181c
DCAF8	ENSG00000132716	1,0143908	0,014784681	up	miR-181c
EIF2A	ENSG00000144895	0,358446265	0,047259493	down	miR-181c
NT5C	ENSG00000125458	1,514033988	0,001428201	up	miR-181c
PSMB9	ENSG00000240065	0,799270923	0,022271307	down	miR-181c
TMEM150A	ENSG00000168890	1,290558135	0,044297099	up	miR-181c
FAM234A	ENSG00000167930	0,782605139	0,001835252	down	miR-181c
AGA	ENSG00000038002	1,093324837	0,037681622	up	miR-181c
AQR	ENSG00000021776	0,524739196	0,012519766	down	miR-181c
ZBTB33	ENSG00000177485	0,981917224	0,001718328	down	miR-181c
MRPS15	ENSG00000116898	0,89010976	0,009795633	down	miR-181c
CPEB2	ENSG00000137449	1,004212299	0,049210537	up	miR-181c
LYPLA2	ENSG00000011009	0,587776206	0,027213682	down	miR-181c
PPP1CB	ENSG00000213639	0,632950318	0,023490177	down	miR-181c
TRIAP1	ENSG00000170855	1,08271917	0,00968091	up	miR-181c
CASP8AP2	ENSG00000118412	0,637018125	0,036108524	down	miR-181c
SEMA5B	ENSG00000082684	2,230090939	0,001107105	up	miR-181c
FAM122B	ENSG00000156504	0,921936608	0,024065165	down	miR-181c
KIAA2018	ENSG00000176542	0,974027672	0,003112329	down	miR-181c
SRPR	ENSG00000182934	0,437710753	0,032698575	down	miR-181c
AMFR	ENSG00000159461	0,88406511	0,023657073	down	miR-181c
APPL1	ENSG00000157500	0,832574142	0,011003059	down	miR-181c
SYNJ2BP	ENSG00000213463	0,661233112	0,007143692	down	miR-181c
SEL1L3	ENSG00000091490	0,909477706	0,002917603	down	miR-181c
ASGR1	ENSG00000141505	2,184532281	0,022533011	up	miR-181c
NAA50	ENSG00000121579	0,770533616	0,023191176	down	miR-181c
GNPDA1	ENSG00000113552	0,986325942	0,018872304	down	miR-181c
MRPL41	ENSG00000182154	0,725293184	0,036193791	down	miR-181c
PDE8A	ENSG00000073417	0,872342457	0,025162626	down	miR-181c
EIF3M	ENSG00000149100	0,546231437	0,017550316	down	miR-181c
PPP2R1A	ENSG00000105568	0,795217224	0,010452746	down	miR-181c
DDX19B	ENSG00000157349	0,761154784	0,007021819	down	miR-181c
TCTN1	ENSG00000204852	1,428651987	0,00116305	up	miR-181c
NBR1	ENSG00000188554	0,989209502	0,009728663	down	miR-181c
ARMC1	ENSG00000104442	0,687507234	0,012287839	down	miR-181c
THOC7	ENSG00000163634	0,821025097	0,007508858	down	miR-181c
PNPLA7	ENSG00000130653	1,081950973	0,047475306	up	miR-181c
ERP44	ENSG00000023318	1,431476592	0,000274696	up	miR-181c
YES1	ENSG00000176105	1,339518282	5,83301E-05	up	miR-181c
SLC12A6	ENSG00000140199	0,741494767	0,033942895	down	miR-181c
IRAK1	ENSG00000184216	0,549062252	0,011748631	down	miR-181c
PLEKHO1	ENSG00000023902	1,426974556	0,019923029	up	miR-181c
HDHD2	ENSG00000167220	0,714763313	0,0381357	down	miR-181c
EIF4EBP2	ENSG00000148730	0,941124953	0,009204802	down	miR-181c
PCNT	ENSG00000160299	0,807128467	0,044958708	down	miR-181c
APLF	ENSG00000169621	1,102198152	0,012724046	up	miR-181c
COMMD4	ENSG00000140365	1,376960281	0,003117421	up	miR-181c
GLDC	ENSG00000178445	1,892366625	0,02615648	up	miR-181c
AC005899.6	ENSG00000274341	2,025835217	0,029126887	up	miR-181c
AL512306.3	ENSG00000240710	1,061667689	0,032408877	up	miR-181c
SMAD4	ENSG00000141646	1,018797641	0,007453907	up	miR-181c
COX7B	ENSG00000131174	1,495653053	0,002430076	up	miR-181c
MRPL3	ENSG00000114686	1,176153926	0,009315568	up	miR-181c
TMEM44-AS1	ENSG00000231770	1,875750318	0,011224437	up	miR-181c
NUCB2	ENSG00000070081	1,284094964	0,008037567	up	miR-181c
AL356356.1	ENSG00000237781	1,5633124	0,014466793	up	miR-181c

KANSL3	ENSG00000114982	1,360251679	0,005954405	up	miR-181c
MYRIP	ENSG00000170011	1,459026159	0,017316576	up	miR-181c
TLR1	ENSG00000174125	1,447282749	0,016799356	up	miR-181c
DCTPP1	ENSG00000179958	1,597665017	0,000392891	up	miR-181c
KIAA1804	ENSG00000143674	1,546558241	0,028911062	up	miR-181c
NDUFS1	ENSG00000023228	0,677502298	0,026960182	down	miR-181c
ERH	ENSG00000100632	1,733504905	0,000189683	up	miR-181c
F7	ENSG00000057593	4,592656496	0,027452041	up	miR-181c
PTEN	ENSG00000171862	0,604020509	0,007519752	down	miR-181c
ABHD5	ENSG00000011198	1,747762076	0,003403081	up	miR-181c
PCYOX1L	ENSG00000145882	2,144723536	0,001048608	up	miR-181c
TMEM107	ENSG00000179029	1,085631068	0,019232888	up	miR-181c
MAL2	ENSG00000147676	1,414925504	0,003153023	up	miR-181c
HMGN1	ENSG00000205581	1,370588814	0,008235136	up	miR-181c
GLO1	ENSG00000124767	1,28824984	0,002277747	up	miR-181c
NDUFA1	ENSG00000125356	0,688934211	0,012737508	down	miR-181c
C11orf31	ENSG00000211450	0,956488103	0,005263103	down	miR-181c
IDH3G	ENSG00000067829	0,702782723	0,024990789	down	miR-181c
NPTN	ENSG00000156642	0,789861782	0,013274004	down	miR-181c
ERI2	ENSG00000196678	1,603781626	0,0024221	up	miR-181c
CDYL2	ENSG00000166446	1,208310381	0,011833611	up	miR-181c
TKFC	ENSG00000149476	0,930396304	0,029890711	down	miR-181c
DEGS1	ENSG00000143753	1,101195299	0,002386462	up	miR-181c
MRPL55	ENSG00000162910	0,814664559	0,005724855	down	miR-181c
KCNJ5	ENSG00000120457	1,110654285	0,030883875	up	miR-181c
SUCLG2	ENSG00000172340	1,05932621	0,021022346	up	miR-181c
HINT2	ENSG00000137133	0,703026611	0,037710656	down	miR-181c
PBX1	ENSG00000185630	0,913536195	0,048939336	down	miR-181c
PNPLA2	ENSG00000177666	0,825299131	0,019524423	down	miR-181c
CEP104	ENSG00000116198	0,603784938	0,02038594	down	miR-181c
ARHGEF10L	ENSG00000074964	0,579901046	0,036451636	down	miR-181c
LBR	ENSG00000143815	1,126133313	0,001863082	up	miR-181c
SPSB1	ENSG00000171621	0,864936535	0,042661851	down	miR-181c
SLC27A5	ENSG00000083807	3,515041593	0,016275347	up	miR-181c
ZNF506	ENSG00000081665	1,55566133	0,005298698	up	miR-181c
PER1	ENSG00000179094	1,150875722	0,035451492	up	miR-181c
SMAD2	ENSG00000175387	0,659114599	0,020922846	down	miR-181c
PPM1B	ENSG00000138032	1,083616789	0,000668042	up	miR-181c
CPSF6	ENSG00000111605	0,994618174	0,00125541	down	miR-181c
RPL26L1	ENSG00000037241	1,250574403	0,007429438	up	miR-181c
ATG9A	ENSG00000198925	0,449711211	0,041865783	down	miR-181c
ZNF747	ENSG00000169955	1,762532155	0,002222824	up	miR-181c
GAS2L1	ENSG00000185340	1,43824448	0,002048884	up	miR-181c
C12orf10	ENSG00000139637	0,748770596	0,004773242	down	miR-181c
LAMTOR4	ENSG00000188186	0,635317072	0,018177528	down	miR-181c
AATF	ENSG00000275700	0,627575508	0,02556782	down	miR-181c
TVP23B	ENSG00000171928	1,413044031	0,035622617	up	miR-181c
C17orf89	ENSG00000224877	0,839054192	0,019598917	down	miR-181c
SMC2	ENSG00000136824	1,321659708	0,001006766	up	miR-181c
C14orf93	ENSG00000100802	1,780808255	0,000756761	up	miR-181c
DPCD	ENSG00000166171	1,338001966	0,008385368	up	miR-181c
LINC00116	ENSG00000175701	1,525656111	0,018739955	up	miR-181c
EIF2AK1	ENSG00000086232	0,533846934	0,00384373	down	miR-181c
CAMSAP1	ENSG00000130559	0,920672519	0,013919509	down	miR-181c
RFTN1	ENSG00000131378	0,750253039	0,014453374	down	miR-181c
CCDC7	ENSG00000216937	1,582994831	0,000402493	up	miR-181c
SLC25A5	ENSG00000005022	1,241430934	0,003181435	up	miR-181c
CMPK1	ENSG00000162368	1,068336084	0,005573103	up	miR-181c
ACBD5	ENSG00000107897	0,830567897	0,014573627	down	miR-181c
FAM210B	ENSG00000124098	1,628902619	0,016315381	up	miR-181c
ZNF467	ENSG00000181444	1,480641733	0,031165456	up	miR-181c
CBLN3	ENSG00000139899	2,579650067	6,63693E-05	up	miR-181c
NDUFB5	ENSG00000136521	0,995528041	0,022061242	down	miR-181c
GNS	ENSG00000135677	0,670881855	0,00774974	down	miR-181c
RTFDC1	ENSG00000022277	0,542130544	0,028161497	down	miR-181c
LARP1B	ENSG00000138709	0,549945347	0,028213607	down	miR-181c
SYS1	ENSG00000204070	0,919364036	0,002947475	down	miR-181c
PCYOX1	ENSG00000116005	0,929654896	0,013530607	down	miR-181c
PPP2CA	ENSG00000113575	0,730155011	0,023235516	down	miR-181c
CD58	ENSG00000116815	0,977266354	0,014608961	down	miR-181c
ALG3	ENSG00000214160	0,88406012	0,007887565	down	miR-181c
ZNF232	ENSG00000167840	1,517363989	0,018989713	up	miR-181c
SMAP1	ENSG00000112305	0,897446441	0,026107975	down	miR-181c
ZC3H6	ENSG00000188177	0,514050147	0,034809075	down	miR-181c
WSB2	ENSG00000176871	1,182399093	0,003734402	up	miR-181c
QTRTD1	ENSG00000151576	0,988223769	0,032522561	down	miR-181c
BX322562.1	ENSG00000273796	2,364750022	0,028107519	up	miR-181c

IDI1	ENSG00000067064	1,372772371	0,019099049	up	miR-181c
RPN1	ENSG00000163902	0,447759449	0,026537324	down	miR-181c
SMAGP	ENSG00000170545	0,815456066	0,026789637	down	miR-181c
TEF	ENSG00000167074	1,115761311	0,019647815	up	miR-181c
DEDD	ENSG00000158796	0,851798436	0,022327544	down	miR-181c
F12	ENSG00000131187	1,706687426	0,024183915	up	miR-181c
ACAP1	ENSG00000072818	0,867075604	0,049174182	down	miR-181c
TXNDC16	ENSG00000087301	1,565600651	0,014811212	up	miR-181c
POLDIP2	ENSG00000004142	0,818579445	0,030401637	down	miR-181c
ZYG11B	ENSG00000162378	0,887993056	0,010070423	down	miR-181c
TGFB1I1	ENSG00000140682	1,068582943	0,040926364	up	miR-181c
GAR1	ENSG00000109534	1,087794292	0,033581777	up	miR-181c
SLC35F3	ENSG00000183780	2,196987922	0,021037635	up	miR-181c
RNASEH1	ENSG00000171865	1,207742746	0,003066388	up	miR-181c
YIPF2	ENSG00000130733	0,777327717	0,023356858	down	miR-181c
ZNF22	ENSG00000165512	1,304676929	0,000329813	up	miR-181c
FBXL13	ENSG00000161040	1,514525298	0,004337617	up	miR-181c
NUP155	ENSG00000113569	1,301233078	0,00059697	up	miR-181c
OCIAD2	ENSG00000145247	1,697630799	0,000594182	up	miR-181c
DRG2	ENSG00000108591	0,606801195	0,030215224	down	miR-181c
WAC-AS1	ENSG00000254635	1,218852179	0,000256557	up	miR-181c
CCL17	ENSG00000102970	1,609073465	0,004773321	up	miR-181c
TRAPPC6B	ENSG00000182400	0,896239273	0,00316397	down	miR-181c
RASEF	ENSG00000165105	1,224087313	0,031507859	up	miR-181c
PPP2R2D	ENSG00000175470	0,941275214	0,001269101	down	miR-181c
AL161421.1	ENSG00000275202	1,076217168	0,041959884	up	miR-181c
TIMM23	ENSG00000265354	0,919758606	0,01110815	down	miR-181c
SMURF1	ENSG00000198742	0,681024887	0,002738429	down	miR-181c
LAT2	ENSG00000086730	1,005487743	0,028807788	up	miR-181c
ADIPOR2	ENSG00000006831	0,662150455	0,033880571	down	miR-181c
ZSCAN29	ENSG00000140265	0,833330897	0,020814729	down	miR-181c
PSMB5	ENSG00000100804	0,826030935	0,007454013	down	miR-181c
SWI5	ENSG00000175854	1,55177001	0,001069327	up	miR-181c
ANO7	ENSG00000146205	1,031366504	0,030179533	up	miR-181c
SMTN	ENSG00000183963	0,725356661	0,020956245	down	miR-181c
UXT	ENSG00000126756	1,57515633	0,003861203	up	miR-181c
PDCD5	ENSG00000105185	1,561882867	0,037867954	up	miR-181c
PCK2	ENSG00000100889	2,665460198	0,006682858	up	miR-181c
ATP2C1	ENSG00000017260	1,248955518	0,002982116	up	miR-181c
NPTX1	ENSG00000171246	1,933975199	0,008659786	up	miR-181c
ATXN7L3B	ENSG00000253719	0,489398942	0,013835155	down	miR-181c
DEPDC7	ENSG00000121690	1,993561318	0,009323398	up	miR-181c
SQRDL	ENSG00000137767	1,042531382	0,005540688	up	miR-181c
AC005261.3	ENSG00000268713	1,993102588	0,000859487	up	miR-181c
EIF4E3	ENSG00000163412	1,716340047	0,007518523	up	miR-181c
ZNF641	ENSG00000167528	1,021968843	0,005674559	up	miR-181c
GIMAP8	ENSG00000171115	2,257786691	0,001088536	up	miR-181c
ADORA1	ENSG00000163485	1,798679699	0,005804616	up	miR-181c
PGM3	ENSG00000013375	0,798478432	0,006950748	down	miR-181c
RNF126	ENSG00000070423	1,005691073	0,001151438	up	miR-181c
NIPSNAP3A	ENSG00000136783	0,93732015	0,0423282	down	miR-181c
WDR26	ENSG00000162923	1,108305664	0,004504575	up	miR-181c
ARF4	ENSG00000168374	1,391754054	0,004196025	up	miR-181c
AAED1	ENSG00000158122	2,838062731	0,009381635	up	miR-181c
ITPK1	ENSG00000100605	0,74629326	0,034905695	down	miR-181c
ALG10B	ENSG00000175548	1,74891425	0,005798216	up	miR-181c
HTATSF1	ENSG00000102241	1,111814068	0,000303847	up	miR-181c
UFL1	ENSG00000014123	0,664658354	0,007922251	down	miR-181c
HNRNPR	ENSG00000125944	0,668271754	0,03505897	down	miR-181c
MARCHF7	ENSG00000136536	0,806144958	0,002324082	down	miR-181c
GID8	ENSG00000101193	0,637741019	0,037422974	down	miR-181c
TSPOAP1-AS1	ENSG00000265148	1,503235394	0,021110485	up	miR-181c
RNF7	ENSG00000114125	0,6889077	0,027200859	down	miR-181c
POMP	ENSG00000132963	0,744177078	0,011004698	down	miR-181c
GLOD4	ENSG00000167699	1,311066832	0,007725472	up	miR-181c
ITGAX	ENSG00000140678	1,080134216	0,009629772	up	miR-181c
SPPL2A	ENSG00000138600	0,768890019	0,014515445	down	miR-181c
ACADL	ENSG00000115361	1,970492926	0,005043246	up	miR-181c
MRPL51	ENSG00000111639	0,968809957	0,011250345	down	miR-181c
REEP6	ENSG00000115255	2,070049013	0,041776792	up	miR-181c
FMNL1	ENSG00000184922	1,096123486	0,010727976	up	miR-181c
ZNF33A	ENSG00000189180	0,786206768	0,005397431	down	miR-181c
VMA21	ENSG00000160131	0,994715879	0,011134593	down	miR-181c
DRICH1	ENSG00000189269	1,097208361	0,023541358	up	miR-181c
WARS	ENSG00000140105	1,313587119	0,000673925	up	miR-181c
TLE1	ENSG00000196781	0,913456842	0,017166901	down	miR-181c
DNPH1	ENSG00000112667	1,329981309	0,002117574	up	miR-181c

NDRG3	ENSG00000101079	1,723844843	0,000627929	up	miR-181c
DHRSX	ENSG00000169084	1,0445349	0,039090345	up	miR-181c
NPM2	ENSG00000158806	2,411699654	0,002860095	up	miR-181c
FRAT1	ENSG00000165879	1,781227457	0,016699225	up	miR-181c
MSANTD4	ENSG00000170903	1,040686472	0,019258188	up	miR-181c
FAM214B	ENSG00000005238	1,476275234	0,014508104	up	miR-181c
THAP7-AS1	ENSG00000230513	1,301226498	0,044570859	up	miR-181c
DNM1L	ENSG00000087470	0,604266646	0,038898302	down	miR-181c
PIN1	ENSG00000127445	0,628981053	0,034961959	down	miR-181c
METTL23	ENSG00000181038	1,748129465	6,33873E-05	up	miR-181c
HIF1AN	ENSG00000166135	0,845154153	0,007525747	down	miR-181c
DNAJC22	ENSG00000178401	1,920083623	0,024167122	up	miR-181c
MOB3A	ENSG00000172081	1,101280709	0,008316568	up	miR-181c
FRMD3	ENSG00000172159	1,893385585	0,009701825	up	miR-181c
IMPACT	ENSG00000154059	0,747020993	0,038370055	down	miR-181c
LINC01843	ENSG00000251169	1,892063221	0,025615411	up	miR-181c
ZNF10	ENSG00000256223	1,858822544	0,006369424	up	miR-181c
SYT13	ENSG00000019505	1,333367631	0,042981928	up	miR-181c
RREB1	ENSG00000124782	0,681056509	0,02073373	down	miR-181c
FGFR4	ENSG00000160867	1,748682667	0,014199526	up	miR-181c
PPP5D1	ENSG00000230510	2,456052617	0,007361786	up	miR-181c
MOCS3	ENSG00000124217	1,522817005	0,001979051	up	miR-181c
MAPK14	ENSG00000112062	0,947579725	0,003972834	down	miR-181c
PGK1	ENSG00000102144	0,994153784	0,000477598	down	miR-181c
COL10A1	ENSG00000123500	1,075209086	0,04152243	up	miR-181c
SECTM1	ENSG00000141574	1,368136218	0,013704294	up	miR-181c
LAMP5	ENSG00000125869	2,208110058	0,006382333	up	miR-181c
F11R	ENSG00000158769	0,770008351	0,011214488	down	miR-181c
GPRIN1	ENSG00000169258	1,409065612	0,020611249	up	miR-181c
RNF14	ENSG00000013561	0,911133202	0,017772086	down	miR-181c
AC091133.1	ENSG00000230532	1,169260539	0,03704836	up	miR-181c
PDZK1IP1	ENSG00000162366	1,223110301	0,020523021	up	miR-181c
LIMS1	ENSG00000169756	0,552849654	0,04391907	down	miR-181c
ZNF441	ENSG00000197044	1,543484169	0,038238646	up	miR-181c
CPSF2	ENSG00000165934	0,863231555	0,001150623	down	miR-181c
TOR4A	ENSG00000198113	0,774841512	0,036771147	down	miR-181c
TMEM185B	ENSG00000226479	2,115900525	0,006881724	up	miR-181c
MPND	ENSG00000008382	1,094058986	0,004676256	up	miR-181c
ZNF699	ENSG00000196110	1,836380503	0,006038984	up	miR-181c
TCP1	ENSG00000120438	1,142016881	0,009492268	up	miR-181c
C12orf75	ENSG00000235162	1,259267242	0,02167927	up	miR-181c
CNIH3	ENSG00000143786	1,996595182	0,026933249	up	miR-181c
DTWD2	ENSG00000169570	2,041317332	0,004676383	up	miR-181c
RNASEK-C17orf49	ENSG00000161939	1,053739269	0,049203215	up	miR-181c
TPD52	ENSG00000076554	1,194959759	0,022172189	up	miR-181c
LMBRD1	ENSG00000168216	0,850477134	0,017390519	down	miR-181c
CCT2	ENSG00000166226	0,841752321	0,01204053	down	miR-181c
C6orf141	ENSG00000197261	2,231232031	0,01907603	up	miR-181c
ZNF805	ENSG00000204524	1,55563451	0,004331079	up	miR-181c
ZNF2	ENSG00000275111	1,587161415	0,020579514	up	miR-181c
AC009506.1	ENSG00000224152	1,397205784	0,022292863	up	miR-181c
SRP9	ENSG00000143742	1,777304798	0,000395359	up	miR-181c
BEND3	ENSG00000178409	1,722524967	0,011323942	up	miR-181c
C1orf210	ENSG00000253313	1,850864384	0,000133674	up	miR-181c
SLC25A47	ENSG00000140107	4,43397697	0,029103596	up	miR-181c
AP004609.3	ENSG00000278376	1,155826753	0,025941813	up	miR-181c
AC130324.1	ENSG00000263531	2,186140224	0,000287002	up	miR-181c
SLC22A7	ENSG00000137204	4,425221131	0,042408115	up	miR-181c
RDH12	ENSG00000139988	0,950764921	0,03755817	down	miR-181c
C14orf132	ENSG00000227051	1,424719983	0,001244014	up	miR-181c
CCL20	ENSG00000115009	2,218431533	0,034630243	up	miR-181c
UEVLD	ENSG00000151116	2,113806533	0,003776456	up	miR-181c
UFSP1	ENSG00000176125	1,439105536	0,010005854	up	miR-181c
SBF2-AS1	ENSG00000246273	2,76536477	0,000493201	up	miR-181c
MOCS1	ENSG00000124615	1,694958875	0,007504815	up	miR-181c
IPCEF1	ENSG00000074706	2,015566022	0,002992128	up	miR-181c
S100A4	ENSG00000196154	2,180073519	0,024960744	up	miR-181c
HIST1H2BN	ENSG00000233822	1,16938642	0,012166876	up	miR-181c
PAN3-AS1	ENSG00000261485	2,222135643	0,006435244	up	miR-181c
CRTAC1	ENSG00000095713	1,792119959	0,005705327	up	miR-181c
PTGER1	ENSG00000160951	1,353651762	0,012103108	up	miR-181c
LINC01123	ENSG00000204588	1,564661523	0,043526372	up	miR-181c
COLEC10	ENSG00000184374	1,190640841	0,046900559	up	miR-181c
PTHLH	ENSG00000087494	1,971958057	0,032905231	up	miR-181c
NDNL2	ENSG00000185115	1,531802307	0,040092891	up	miR-181c
CAMK1G	ENSG00000008118	2,309571032	0,033045455	up	miR-181c
HRCT1	ENSG00000196196	1,972189658	0,0040113	up	miR-181c

ZNF776	ENSG00000152443	1,900369154	0,006444469	up	miR-181c
LRRC73	ENSG00000204052	1,519055769	0,033870796	up	miR-181c
LINC01705	ENSG00000232679	2,219789754	0,028211509	up	miR-181c
TMEM17	ENSG00000186889	1,952450112	0,028022468	up	miR-181c
