# Supplementary material for: A MiR181/Sirtuin1 regulatory circuit modulates drug response in biliary cancers
Source: Clin Exp Med. 2024 Apr 10;24(1):74. doi: 10.1007/s10238-024-01332-0 (PMC11006774; doi:10.1007/s10238-024-01332-0)
Supplement: Supplementary file 5 — Supplementary file5 (PDF 114 KB) [file 10238_2024_1332_MOESM5_ESM.pdf]

Table S3. Pearson's correlation of miR-181c and miR-181d and predicted target genes (filtered for gene expression's p-value < 0.05) in BTC patients

GENE NAME	correlation with miR-181c	pval	correlation with miR-181d	pval
ENAH	-0,902375451	0,102620989	0,529073322	0,152248135
NPEPPS	-0,41116716	0,013206184	0,288276555	0,026772668
FBXO34	-0,376840728	5,82919E-13	-0,071684643	1,89418E-11
CDYL	-0,253014159	1,40065E-10	-0,021976241	3,30994E-09
TRIM2	-0,250925441	9,5328E-11	-0,128052986	2,34747E-09
GREM1	-0,244792563	2,76296E-06	0,077361681	1,72654E-05
LPCAT1	-0,239707166	8,92519E-12	-0,0100696	2,56467E-10
SIRT1	-0,238139813	1,81E-10	-0,145712638	2,79E-09
GPD2	-0,237632053	5,40246E-13	0,066257621	1,75506E-11
TRAK1	-0,234246098	3,20344E-08	0,094902255	4,30619E-07
SRSF10	-0,222888566	4,66006E-07	0,032305467	4,81777E-06
RIMKLB	-0,219207057	2,12258E-12	-0,021354076	6,61967E-11
VHL	-0,217069477	5,33331E-10	0,095317632	1,18766E-08
AAK1	-0,213396677	0,009720147	-0,004175054	0,02080578
HLTF	-0,207170734	1,48235E-11	0,269153747	4,31087E-10
RLIM	-0,205187254	9,62239E-12	0,058312682	2,85648E-10
ATXN7	-0,198900042	0,006111119	0,30916583	0,014822725
DNAJC7	-0,194620392	0,004705671	0,056995322	0,011986959
ITGA3	-0,193203556	0,000308907	0,170948367	0,001004231
ZNF44	-0,191589175	3,70791E-12	-0,076373555	1,13942E-10
VPS36	-0,191413062	8,43465E-10	-0,173146967	2,02684E-08
ITGA6	-0,187669584	1,01319E-05	0,014688262	5,91482E-05
DHX57	-0,187516227	2,67806E-11	0,16973489	7,38024E-10
DDX60L	-0,181421954	4,93044E-09	0,01628315	7,70715E-08
NOTCH4	-0,180538849	2,85281E-12	-0,177113241	8,79274E-11
HOXB4	-0,172554522	7,99053E-11	0,084215727	1,73244E-09
YWHAG	-0,15521254	0,000196212	0,241919621	0,000843013
ERAP2	-0,154951032	6,39885E-12	-0,227945438	1,86301E-10
PGM2L1	-0,150953937	2,63281E-07	-0,118979877	2,80948E-06
DENND4C	-0,138811417	6,09574E-11	0,039530405	1,48585E-09
ATM	-0,137600413	1,21293E-05	0,098927048	7,46377E-05
LATS1	-0,136899376	4,70117E-12	-0,042327006	1,38134E-10
ADCY1	-0,136493599	2,33035E-14	-0,16869463	8,20986E-13
C1orf52	-0,13642592	8,18299E-13	0,05514939	2,6305E-11
CCP110	-0,135414073	3,5711E-11	0,032257273	9,12423E-10
DNAL1	-0,13331787	2,50341E-12	-0,086470753	7,27528E-11
MINK1	-0,132301634	3,56785E-11	0,278624482	9,49548E-10
DNAJA4	-0,123380069	2,07062E-11	0,10098077	4,98868E-10
PRKCD	-0,122558631	8,07656E-12	-0,037547483	2,17286E-10
GNB4	-0,119299422	4,13644E-10	-0,111156986	9,61507E-09
ZBTB43	-0,116756991	2,7588E-09	-0,20220511	5,03984E-08
ZBTB41	-0,11670869	1,42818E-12	-0,018544703	4,53195E-11
GOLGA8A	-0,11555633	1,60579E-09	-0,317228485	2,69905E-08
MBOAT2	-0,115172885	1,5735E-12	-0,117308876	4,79697E-11
ZDHHC3	-0,114951839	3,14337E-10	0,146107858	6,93737E-09
AFAP1	-0,112475808	1,52372E-11	0,004939498	4,29166E-10
ADGRF5	-0,111194345	8,61166E-13	-0,044991387	2,76859E-11
CCDC88C	-0,107276238	5,84641E-12	0,054680042	1,64754E-10
MAP3K3	-0,106965351	2,25597E-08	-0,090364201	3,59772E-07
RNF34	-0,106752569	1,29618E-11	0,066359031	3,6875E-10
ALG13	-0,106469635	2,04061E-12	-0,231137573	6,16526E-11
TANC2	-0,097795078	6,31294E-11	0,178621356	1,58084E-09
NAPB	-0,096115865	7,03787E-13	0,157242362	2,2769E-11
ETNK1	-0,090672059	7,40132E-12	0,165083263	2,14328E-10
ESR1	-0,089820417	9,73317E-14	-0,204541075	3,07654E-12
ITGA2	-0,089561525	2,21742E-09	0,02933415	3,43569E-08
TULP4	-0,088972099	1,94231E-10	-0,044356506	4,66045E-09
B3GNT5	-0,088853047	3,65565E-12	0,026138341	1,04376E-10
BRD1	-0,086477255	9,56962E-12	-0,003395233	2,75599E-10
ADAM28	-0,079088505	6,69737E-08	-0,073405347	7,17194E-07
KIAA1217	-0,078119901	1,03316E-05	-0,081950559	6,41521E-05
B3GALT5	-0,077543178	1,26395E-11	-0,106823011	3,21095E-10
ADAMTS6	-0,077303595	2,20848E-13	0,072076699	7,35723E-12
RASSF1	-0,076484802	3,66358E-11	0,023665019	9,9779E-10
STXBP5	-0,076304605	5,93701E-11	-0,163502217	1,48223E-09
MTMR9	-0,073244933	7,96079E-12	-0,054942164	2,23638E-10
RNF145	-0,073208781	2,16212E-09	0,130395188	4,23939E-08
ACVR2A	-0,070537592	2,006E-12	-0,113917129	6,26109E-11

ZEB2	-0,06887692	0,003288112	-0,183060647	0,008089194
ZFP62	-0,068273284	7,69761E-13	-0,156616148	2,46673E-11
COL16A1	-0,068161679	4,45317E-12	-0,107662799	1,35477E-10
ANKFY1	-0,067318875	2,83545E-10	-0,058365654	6,50544E-09
PTPRE	-0,066261886	4,17668E-10	-0,153623381	9,40664E-09
ZC3HAV1	-0,065392647	1,46291E-07	-0,195613006	1,76658E-06
SLC24A3	-0,063569323	3,96369E-13	0,046157993	1,30181E-11
CHD7	-0,06310379	1,00359E-05	0,047839548	6,83129E-05
MORC3	-0,060521237	7,33673E-10	-0,021723387	1,66566E-08
SLAIN2	-0,060359778	2,72527E-10	-0,186960506	6,49408E-09
CNOT2	-0,05888253	0,004279406	-0,016840455	0,011306915
CLASP1	-0,058423995	3,07212E-11	0,095497216	8,17426E-10
GNAQ	-0,054904724	1,5543E-08	0,149734753	2,61939E-07
LCOR	-0,052699234	8,41439E-09	0,17190964	1,41326E-07
ANKRD44	-0,05166873	6,65276E-12	-0,17237441	1,86859E-10
CEP97	-0,05126882	9,2619E-14	0,230205113	3,16839E-12
CEP120	-0,050659812	1,69297E-12	0,034436182	5,35263E-11
CREB1	-0,049164254	2,03773E-10	0,048456134	5,0331E-09
ITGB8	-0,045794628	1,19143E-10	0,025714019	2,70381E-09
ZNF664	-0,045233716	1,171E-11	0,162444718	3,22919E-10
PAXBP1	-0,044950006	5,42605E-10	-0,197045364	1,28733E-08
CDC5L	-0,043738508	5,365E-08	0,127698449	7,76304E-07
PLXNC1	-0,041151986	9,51129E-11	-0,085214739	2,19842E-09
DCBLD2	-0,041053056	5,37171E-11	-0,051086805	1,26123E-09
SOAT1	-0,040995164	6,05869E-12	0,236682591	1,77553E-10
CARD11	-0,040336797	1,23301E-13	0,014269768	4,18623E-12
PPIP5K2	-0,038873954	1,19997E-09	-0,044272684	2,42925E-08
DOCK7	-0,038849969	2,24171E-12	0,093567626	6,76352E-11
TRNP1	-0,038655058	3,04448E-12	0,129798307	9,04674E-11
DIDO1	-0,037312077	1,68442E-10	0,261389201	4,20671E-09
SIPA1L2	-0,033365359	5,69064E-12	0,100373925	1,72663E-10
OSBPL3	-0,032334865	1,92215E-09	0,123811533	3,54521E-08
PHF2	-0,032107943	1,454E-11	-0,202650945	3,98654E-10
AVL9	-0,030567034	2,26549E-11	-0,072264743	6,29054E-10
MSI2	-0,029604321	0,00061112	-0,280749921	0,001968758
PARP11	-0,028591548	1,43646E-13	-0,182893966	4,85582E-12
JARID2	-0,028262359	1,69079E-10	0,028712733	3,95364E-09
CCNDBP1	-0,027278491	4,81553E-12	0,153616771	1,417E-10
FOXK1	-0,0271167	2,0353E-11	-0,070856261	5,70776E-10
DGKH	-0,02200577	1,2811E-07	0,088387468	1,41975E-06
FAM135A	-0,02079908	2,62216E-11	0,054800544	7,20764E-10
SPOCK1	-0,019762123	5,82066E-12	-0,146414554	1,51976E-10
ZNF562	-0,018801664	1,28179E-07	-0,013333742	1,46613E-06
ZNF217	-0,018725732	2,18697E-10	0,243895529	5,2377E-09
ATP8B2	-0,018222455	9,07749E-13	-0,220942326	2,90227E-11
ARNT2	-0,017145004	1,72366E-13	0,295749463	5,58451E-12
INPP4A	-0,015672451	1,73853E-12	-0,026227541	5,2411E-11
CRIM1	-0,014080017	5,8253E-10	-0,093872155	1,29212E-08
PTPDC1	-0,013750845	9,38254E-13	-0,052951126	3,00052E-11
FSD1L	-0,011522749	4,3253E-14	0,164155887	1,50572E-12
RAP1B	-0,010848291	9,85958E-09	-0,015754506	1,63859E-07
PUM1	-0,010608278	0,000926414	0,020665864	0,00308103
PURB	-0,010240462	4,88105E-10	-0,060204051	1,16182E-08
SESN3	-0,009043938	1,23428E-10	-0,043925627	3,02075E-09
ENTPD6	-0,008721831	1,29081E-09	0,036404186	2,34269E-08
ENDOD1	-0,007420766	3,21471E-11	-0,161774132	8,25471E-10
PTPN4	-0,007359671	6,97616E-12	0,397605665	1,9555E-10
ACER3	-0,006694565	3,74502E-11	0,120519922	9,25017E-10
TMEM87B	-0,005778603	1,74808E-10	0,057063781	4,34173E-09
DMXL2	-0,004187488	3,05875E-10	0,067335952	7,19805E-09
KRAS	-0,004057157	8,10258E-10	0,137989092	1,65958E-08
ERO1A	-0,002758805	7,09663E-08	0,141107972	8,87379E-07
PSPC1	-0,000447645	6,01413E-12	0,010127266	1,81613E-10
ARHGEF7	-6,68113E-05	1,40516E-11	0,024663052	3,99396E-10
KIF3B	0,001123362	1,82518E-09	-0,112816294	3,72956E-08
XRN1	0,001143671	3,24321E-05	-0,215456277	0,000181581
TTF1	0,002964626	2,25846E-10	0,237086429	5,37825E-09
MAEA	0,00323114	1,06996E-11	0,047088124	3,18289E-10
UBP1	0,004856496	3,11659E-09	0,276189763	6,00179E-08
PDCD4	0,00504728	1,56845E-05	-0,124554849	9,77117E-05
TGFBI	0,007630484	5,71333E-05	-0,010859022	0,000266047
CD46	0,013577103	0,004395407	0,437615408	0,011256843
GRK6	0,014424409	7,90019E-13	-0,044128138	2,55147E-11
GPR88	0,016513395	9,62884E-15	-0,159590335	3,45867E-13
DIP2C	0,017365231	3,71795E-09	-0,138734249	6,69262E-08
CUL5	0,022636141	9,97428E-09	-0,082745657	1,76994E-07
C2CD5	0,022937337	2,40961E-13	0,256605544	8,02613E-12

ANKRD52	0,024578892	2,44832E-11	0,303372901	6,58843E-10
INO80D	0,024749056	8,85795E-11	0,004109303	2,43278E-09
GFPT1	0,025295028	5,78198E-08	-0,017972142	7,80167E-07
KIF1B	0,027239222	1,14059E-08	0,249898677	1,95729E-07
CDS1	0,028042833	2,42714E-12	0,077305047	7,29718E-11
CHMP2B	0,028858752	4,74776E-10	-0,025242717	1,09753E-08
USP33	0,030332202	1,72907E-06	0,025107898	1,5609E-05
PAM	0,030932879	1,75272E-10	0,062700285	4,33558E-09
ATXN1	0,032940592	4,50716E-07	0,224765409	4,96059E-06
PPFIA1	0,03502691	0,000488202	-0,160974646	0,001803139
MAP1A	0,035033429	1,23062E-09	-0,096078073	2,31045E-08
IFT57	0,038664702	1,83517E-12	0,074933329	5,77398E-11
ADRA1A	0,040440856	1,13046E-14	-0,161442504	4,04683E-13
SRGAP1	0,047884536	7,23444E-05	0,024339122	0,000362646
ZDHHC17	0,047998135	5,29007E-12	0,058650092	1,59768E-10
MFAP3L	0,052017403	1,62622E-14	0,017901487	5,78051E-13
CREBZF	0,052264083	7,68495E-10	0,167710828	1,74658E-08
ZNF83	0,053418643	1,04602E-10	0,381052181	2,65832E-09
RBBP7	0,053550546	3,06218E-09	0,252856833	5,74681E-08
RIN2	0,053650163	6,42058E-12	0,181019027	1,93346E-10
CRYBG3	0,055259875	1,21487E-09	-0,061276198	2,4469E-08
MAPK1IP1L	0,056191547	2,40006E-10	0,052845474	6,26416E-09
RANGAP1	0,057215804	1,13401E-06	0,124078838	9,8714E-06
CD2AP	0,061084167	1,17963E-05	0,077911576	7,71186E-05
ATP2B2	0,061973162	3,4269E-14	0,027008817	1,15331E-12
CLOCK	0,062632461	6,52939E-11	-0,003969672	1,7487E-09
SLC25A24	0,062683206	5,81841E-13	-0,084463819	1,89102E-11
CEP83	0,064113382	4,99087E-12	0,248786853	1,46672E-10
NPAT	0,06593453	2,88763E-12	-0,017186695	8,95094E-11
GLIS3	0,068718399	2,34093E-10	0,144512857	5,74742E-09
ZNF544	0,071180731	1,5057E-12	0,230702914	4,58182E-11
C12orf49	0,071687563	5,70504E-12	0,094424668	1,72276E-10
LMBRD2	0,072631227	1,28054E-11	0,221132356	3,7647E-10
EPC2	0,074989323	1,06476E-11	0,190495525	3,04156E-10
IPO7	0,075789019	9,06635E-08	0,213297938	1,26293E-06
SPAG1	0,077292648	6,95469E-13	0,118011522	2,24201E-11
ZNF121	0,07861016	9,31701E-10	0,252087177	1,83918E-08
ACAP2	0,078775096	3,08396E-09	0,218279186	6,16404E-08
RAI1	0,079356771	6,42158E-12	0,032643157	1,86643E-10
ANKRD50	0,079644204	5,98609E-12	0,205007942	1,80194E-10
FMNL2	0,080687993	1,44375E-08	0,053725526	2,27105E-07
JADE2	0,081056243	3,07576E-11	0,033006099	8,15016E-10
SEPT8	0,081176677	4,65231E-11	-0,023748087	1,14464E-09
NR2C2	0,081180431	1,13246E-12	0,375194505	3,60936E-11
SORD	0,081929838	1,10662E-14	0,061570608	3,96428E-13
STIM2	0,082344573	6,54939E-10	-0,002148957	1,4945E-08
PDPK1	0,083008042	1,72146E-09	0,024935455	3,85598E-08
ARFGEF3	0,083045411	7,28046E-12	0,266480705	2,01818E-10
CCDC6	0,08347487	5,81549E-10	0,046599859	1,24992E-08
FRYL	0,085046053	9,95074E-09	0,035137208	1,76178E-07
BCL9	0,085694475	2,80695E-13	0,291927749	9,28637E-12
TET2	0,087842337	1,81359E-09	0,048017516	4,05897E-08
PAN3	0,08947334	5,82994E-12	0,064346061	1,64428E-10
KIF3A	0,089541913	3,8564E-11	0,344395849	1,09101E-09
GPX8	0,089679091	8,62467E-12	0,106523946	2,4745E-10
ARL2BP	0,092608531	2,77734E-12	0,002680412	8,63164E-11
GSE1	0,093065587	2,24345E-06	0,089787302	1,82965E-05
TGFBR1	0,093316307	8,09293E-12	0,226343648	2,34598E-10
DIP2B	0,094996057	2,17215E-11	0,142524327	5,67337E-10
RAB3GAP1	0,09543642	1,05689E-08	0,117938765	1,86505E-07
FAM122B	0,097185075	3,92669E-12	0,316515294	1,11771E-10
G2E3	0,100020395	1,16217E-12	0,054531545	3,70523E-11
LRBA	0,103050709	3,41354E-08	-0,153912733	4,86704E-07
KLF15	0,103869501	2,6853E-14	-0,148264737	9,43327E-13
WDR82	0,108330005	1,00812E-09	0,146359625	2,25655E-08
PDCD6IP	0,110091086	1,14822E-09	0,109032073	2,46634E-08
TNIK	0,1125394	2,92471E-09	-0,105178016	4,81475E-08
MTF2	0,113838254	3,68617E-10	0,122130538	8,86465E-09
C2orf69	0,115406165	1,06275E-12	0,010139855	3,40444E-11
VPS41	0,115998581	6,57367E-07	-0,02972013	6,28899E-06
MCC	0,116159187	4,11934E-14	-0,136597437	1,43273E-12
AGFG1	0,116321323	1,09743E-06	0,172119906	1,01992E-05
NFAT5	0,116573298	0,000143056	-0,036131303	0,000646328
MEF2A	0,117153544	3,91321E-08	-0,288777966	5,55128E-07
AP1S3	0,117190039	2,687E-11	0,064776004	6,66945E-10
VPS13B	0,118771648	1,75832E-11	-0,093101318	4,95294E-10
ATXN3	0,120422228	1,33842E-07	-0,070305118	1,78002E-06

CD302	0,120487952	1,8256E-13	-0,093930653	6,14072E-12
SRGAP2	0,121503691	1,53628E-13	0,045550589	5,19374E-12
OSBPL8	0,121945991	3,67851E-06	0,008558541	2,73548E-05
IGF2BP2	0,122000943	4,03129E-06	0,082858736	2,79837E-05
REST	0,122061685	0,000189683	0,142261593	0,000840444
ETV6	0,122230868	0,00020551	-0,029715284	0,000880483
NAALADL2	0,123282432	4,67881E-12	0,114450698	1,36491E-10
AKT3	0,125515401	2,78972E-10	0,071228395	6,76159E-09
PTBP3	0,127641995	0,001060549	0,320084671	0,003493384
SPIRE1	0,127731027	8,79375E-12	0,309445635	2,61405E-10
CDKL5	0,127917395	9,25315E-13	0,077719876	2,77378E-11
LUZP1	0,128407869	8,21561E-09	-0,011891556	1,56245E-07
PIAS1	0,130348361	7,21781E-09	0,107412733	1,33739E-07
OSBPL2	0,13045603	6,49488E-10	0,050452526	1,38391E-08
IPO8	0,130617885	1,39717E-11	0,228500168	3,97764E-10
GPATCH2L	0,132260888	7,49051E-08	0,119240733	9,9761E-07
CCNK	0,134733075	4,54782E-12	-0,030713064	1,25323E-10
NAA15	0,135590781	3,89932E-10	0,104753708	8,55672E-09
CAPRIN1	0,137060475	5,42091E-09	0,083622504	1,05013E-07
USP46	0,137286049	4,96034E-13	0,063144647	1,56741E-11
F2R	0,138200438	1,30046E-11	-0,012130656	3,78719E-10
RNF169	0,139740353	1,34896E-11	0,047522326	3,94093E-10
PRDX3	0,139833487	8,91344E-14	0,216601129	3,05122E-12
RALA	0,140316137	2,18471E-07	0,167866791	2,34816E-06
CAND1	0,143327593	2,77411E-08	0,297082322	4,13453E-07
GIGYF1	0,144329452	8,19835E-06	0,199439022	5,65151E-05
STRN	0,144860084	4,73281E-12	0,016705089	1,38975E-10
HIPK1	0,144938436	1,47352E-09	0,205445425	3,12865E-08
RALGAPB	0,14515674	8,5899E-12	0,245363821	2,4862E-10
PLEKHJ1	0,145774219	5,79752E-11	0,099797782	1,55553E-09
AFTPH	0,145855483	3,19103E-09	0,175284266	5,81241E-08
CTDSPL	0,147270094	1,03828E-08	0,167597401	1,60916E-07
NOTCH2	0,14834694	3,3541E-12	0,003948018	1,03392E-10
PEAK1	0,148979091	1,27938E-08	-0,21793481	2,10285E-07
LTBP2	0,149515304	1,47493E-05	-0,088054643	8,11408E-05
PDE5A	0,152269062	5,48603E-12	-0,06851962	1,63818E-10
RLF	0,153380126	2,6677E-11	0,222755803	7,1634E-10
ARL5A	0,154477817	5,5133E-07	0,462764182	5,4787E-06
TMEM131	0,15464481	8,5993E-12	-0,021768381	2,48631E-10
SIN3B	0,155599159	1,39877E-10	-0,02835201	3,40089E-09
NACC2	0,15781234	9,13281E-13	0,220476278	2,93669E-11
NAB1	0,158716642	2,64155E-08	0,42007927	3,82277E-07
GOLGA8B	0,158795289	1,45365E-06	0,150445279	1,17535E-05
EIF2AK1	0,161600079	4,25211E-08	0,276301278	6,1874E-07
PPP2R3A	0,161620087	4,10367E-13	0,033178901	1,34661E-11
TNS1	0,163547906	0,000998226	0,229189313	0,003110407
PIK3C2A	0,163557008	0,003569108	-0,026964405	0,009286277
CBLB	0,164204242	2,82754E-08	0,040915636	3,96079E-07
SSB	0,166976929	3,14138E-05	0,161510037	0,000176525
IFT81	0,167494185	1,91165E-13	0,227449868	6,4118E-12
DSE	0,1704541	1,53112E-10	-0,017273624	3,94967E-09
PPP1R12B	0,17485683	1,25727E-07	-0,095465375	1,47894E-06
MYO1E	0,175777479	1,94439E-07	-0,113840113	2,24125E-06
TIA1	0,179721212	9,56334E-07	0,076118075	8,69105E-06
RNMT	0,182209285	4,61782E-09	0,259325955	9,03181E-08
KLF7	0,18313364	9,25085E-11	0,020388397	2,28878E-09
ARF3	0,185592656	0,001033631	-0,178705203	0,003406312
MGA	0,191440984	1,67318E-09	0,229777985	3,63657E-08
SLC35E1	0,192035032	1,034E-07	0,040864057	1,35334E-06
CAMTA2	0,193780505	2,00432E-12	0,181350989	6,28989E-11
CREBRF	0,195888914	0,005854148	0,108753476	0,013811281
AFF1	0,197330923	9,92337E-10	-0,114016581	2,086E-08
BIRC6	0,198799399	7,96847E-07	0,09011727	8,72508E-06
GTSE1	0,198924228	7,79107E-14	0,268070084	2,66835E-12
CPSF6	0,202579292	1,16278E-10	0,208623578	2,58198E-09
CDC42BPA	0,202834468	1,16431E-05	-0,187989446	6,95134E-05
FAM13B	0,203656942	1,22949E-12	0,311407343	3,92273E-11
POU2F1	0,209160055	1,04907E-09	0,269009466	2,35631E-08
CTTNBP2NL	0,211969226	2,69721E-09	-0,125162277	5,089E-08
KATNBL1	0,212396094	3,94866E-10	0,105344181	8,97078E-09
TMEM165	0,21308056	1,92734E-06	0,072045034	1,62802E-05
KPNA4	0,213480511	0,000348716	0,096689034	0,001356135
SNX5	0,21415333	1,58808E-06	0,428061718	1,35675E-05
LEMD3	0,214743062	2,81326E-12	0,21514095	8,71345E-11
G3BP2	0,216153694	1,25883E-10	0,035303856	3,28547E-09
QSER1	0,216479184	1,12307E-09	0,245757075	2,48783E-08
PAK4	0,217728535	7,60461E-10	0,212469043	1,56256E-08

XPO7	0,22005962	6,08035E-10	0,362979256	1,38675E-08
GLS	0,221393157	5,26482E-06	0,098285319	4,06962E-05
PPP2R5E	0,222337762	2,34897E-05	0,119466596	0,000142597
ZNF704	0,224643334	9,67632E-11	-0,130857909	2,30166E-09
GDI1	0,224908403	1,4691E-08	0,287303099	2,24034E-07
NBEA	0,225044441	1,41415E-11	0,095338384	3,72465E-10
ETS1	0,227657783	4,69793E-09	-0,104608212	7,56721E-08
CAPRIN2	0,232903585	2,80743E-09	0,323633291	4,76985E-08
DYNC2H1	0,233029159	2,23923E-08	-0,041875051	2,94714E-07
DNAJC13	0,233463817	1,42625E-09	0,001919591	2,75776E-08
MAPK1	0,234545713	8,1348E-10	0,086312715	1,8482E-08
MECP2	0,235776012	4,7371E-08	0,157064778	6,84672E-07
SBNO1	0,241194294	2,62959E-05	0,385339742	0,000153461
ILF3	0,243668337	0,084865813	-0,633881995	0,131816392
PHTF2	0,244314696	4,8354E-12	0,096558324	1,423E-10
AGO4	0,246086779	1,00533E-11	0,220267843	2,78993E-10
ZC3H6	0,24922022	7,31204E-11	-0,116795884	2,01963E-09
SRPK2	0,250340335	6,84131E-09	-0,009222326	1,12294E-07
CTNNA1	0,252130447	5,25801E-07	0,20785326	5,39505E-06
RAB3IP	0,253273524	9,88018E-08	0,299458912	1,0057E-06
FAM102A	0,254298144	2,66583E-09	0,092027998	5,31768E-08
ZBTB4	0,25951442	4,93266E-10	0,103799838	1,14135E-08
TMEM87A	0,263818165	7,23803E-07	0,139873312	7,51391E-06
DNAJC5	0,272588915	9,20526E-08	0,105911487	1,10365E-06
PCNP	0,274548603	1,30873E-08	0,205624012	2,28009E-07
PTBP2	0,27610622	4,11187E-13	0,171050496	1,3532E-11
ZNF37A	0,276204841	1,26288E-08	0,144956318	2,00834E-07
TGFBRAP1	0,277624519	4,76961E-13	0,313058364	1,56477E-11
RASSF8	0,280425632	1,49344E-11	0,029258248	4,20593E-10
ANTXR1	0,283832689	8,06488E-06	0,070617089	4,60228E-05
KLHL42	0,290077447	1,20164E-09	0,113516976	2,19754E-08
PPP1R9A	0,290148589	5,45506E-08	0,126099292	6,4713E-07
SLC25A36	0,290316178	1,6461E-08	0,181998802	2,59379E-07
CDC73	0,301833682	3,6523E-08	0,107194093	5,53495E-07
SECISBP2	0,306592746	2,33598E-08	-0,080467032	3,39992E-07
BRWD1	0,307076281	1,26321E-06	0,245260303	1,12884E-05
YWHAZ	0,317028858	0,005015992	0,094255461	0,01160902
ZMYND11	0,320440466	2,14763E-07	0,049096154	2,39142E-06
TMF1	0,323201579	1,88197E-05	0,166772582	0,00011574
DNAJC21	0,330678419	2,2634E-07	0,151248486	2,67589E-06
MMP14	0,3329941	0,035698133	0,676298348	0,059087257
YLPM1	0,341003773	0,00010273	0,337583178	0,000460822
MAP1B	0,341020946	0,005569409	-0,18456093	0,012794016
ZNF326	0,352771396	1,41184E-08	0,153406239	2,37942E-07
SMC6	0,35698859	1,88148E-06	0,264135995	1,58768E-05
MFSD6	0,358880473	1,05687E-12	0,277181196	3,37246E-11
CAMSAP2	0,361907987	1,04861E-07	0,267345768	1,24558E-06
INO80	0,367227837	3,48893E-08	0,068677259	5,13446E-07
ZNF655	0,370937787	1,17809E-07	0,385517145	1,47948E-06
GPBP1	0,441802315	0,008001667	0,427969031	0,017348765
PAFAH1B2	0,44276804	0,000274609	0,028324496	0,001085269
TBL1XR1	0,470181436	2,62592E-06	0,339986377	2,04642E-05
TNPO1	0,51332461	0,000384335	0,438357068	0,001441994
