# Supplementary material for: A MiR181/Sirtuin1 regulatory circuit modulates drug response in biliary cancers
Source: Clin Exp Med. 2024 Apr 10;24(1):74. doi: 10.1007/s10238-024-01332-0 (PMC11006774; doi:10.1007/s10238-024-01332-0)
Supplement: Supplementary file 6 — Supplementary file6 (PDF 91 KB) [file 10238_2024_1332_MOESM6_ESM.pdf]

Table S4. GSEA querying Molecular Signature database (MSigDB) of DE target genes in BTC patients expressing high miRNA-181c and -181d versus low, using hallmark, wikipathways, reactome and KEGG pathways

GENE SET	pval	FDR	ES	NES	Pathways
ANDROGEN_RESPONSE	0,000	0,000	-0,618	-23,210	Hallmark
BILE_ACID_METABOLISM	0,000	0,002	-0,838	-21,683	
MYC_TARGETS_V1	0,039	0,122	-0,396	-15,556	
MYC_TARGETS_V2	0,060	0,135	-0,764	-15,078	
HEDGEHOG_SIGNALING	0,062	0,149	-0,723	-14,451	
ADIPOGENESIS	0,099	0,135	-0,376	-14,394	
OXIDATIVE_PHOSPHORYLATION	0,085	0,229	-0,452	-13,198	
FATTY_ACID_METABOLISM	0,223	0,351	-0,438	-12,178	
ESTROGEN_RESPONSE_EARLY	0,231	0,362	-0,220	-11,888	
ESTROGEN_RESPONSE_LATE	0,743	0,837	-0,192	-0,810	
PEROXISOME	0,308	0,458	-0,443	-11,096	
HYPOXIA	0,381	0,644	-0,177	-0,989	
NOTCH_SIGNALING	0,470	0,621	-0,581	-0,977	
MTORC1_SIGNALING	0,455	0,590	-0,174	-0,971	
PI3K_AKT_MTOR_SIGNALING	0,828	0,820	-0,181	-0,789	
PROTEIN_SECRETION	0,988	0,961	-0,221	-0,632	
P53_TRANSCRIPTIONAL_GENE_NETWORK	0,014	0,485	0,680	16,207	WikiPathways
APOPTOSIS	0,463	0,925	0,484	0,999	
ANGIOGENESIS	0,048	0,238	-0,695	-15,721	
EGFR_TYROSINE_KINASE_INHIBITOR_RESIS	0,054	0,333	-0,387	-14,700	
TARGET_OF_RAPAMYCIN_SIGNALING	0,135	0,434	-0,609	-13,240	
PI3KAKTMTOR_SIGNALING_PATHWAY_AND	0,220	0,561	-0,482	-12,018	
MODULATION_OF_PI3KAKTMTOR_SIGNALIN	0,102	0,433	-0,961	-12,848	
WNT_SIGNALING	0,237	0,636	-0,304	-11,304	
DNA_DAMAGE_RESPONSE_ONLY_ATM_DEP	0,167	0,563	-0,283	-11,993	
DNA_MISMATCH_REPAIR	0,801	0,950	-0,601	-0,802	
MAPK_CASCADE	0,337	0,656	-0,482	-10,905	
NRF2_PATHWAY	0,018	0,336	0,811	15,579	
NUCLEAR_RECEPTORS	0,001	0,018	-0,676	-20,550	
ESTROGEN_RECEPTOR_PATHWAY	0,001	0,050	-0,968	-18,889	
FATTY_ACID_BIOSYNTHESIS	0,001	0,019	-0,939	-21,452	
OMEGA9_FATTY_ACID_SYNTHESIS	0,001	0,059	-0,972	-18,915	
FATTY_ACID_TRANSPORTERS	0,003	0,064	-0,913	-18,271	
OXYSTEROLS_DERIVED_FROM_CHOLESTER	0,025	0,411	-0,984	-13,213	
FATTY_ACID_BETAOXIDATION	0,210	0,562	-0,523	-11,975	
MEVALONATE_ARM_OF_CHOLESTEROL_BI	0,288	0,634	-0,837	-11,291	
CHOLESTEROL_SYNTHESIS_DISORDERS	0,330	0,641	-0,837	-11,101	
CHOLESTEROL_BIOSYNTHESIS_PATHWAY	0,351	0,635	-0,837	-11,027	
CHOLESTEROL_METABOLISM	0,859	0,962	-0,455	-0,749	
LIPID_METABOLISM_PATHWAY	0,820	0,975	-0,385	-0,758	
CHOLESTEROL_METABOLISM	0,005	0,062	-0,731	-18,412	
SREBF_LIPID_HOMEOSTASIS	0,028	0,266	-0,803	-15,518	
STEROL_REGULATORY_ELEMENTBINDING_	0,196	0,558	-0,311	-12,046	
TRANSCRIPTIONAL_CASCADE_REGULATIN	0,027	0,295	-0,933	-15,313	
ENERGY_METABOLISM	0,845	0,958	-0,261	-0,786	
MITOCHONDRIAL_COMPLEX_IV_ASSEMBLY	0,282	0,618	-0,879	-11,560	
MITOCHONDRIAL_FATTY_ACID_BETAOXIDA	0,006	0,242	-0,967	-15,781	
MITOCHONDRIAL_COMPLEX_I_ASSEMBLY_	0,032	0,332	-0,898	-14,635	
ELECTRON_TRANSPORT_CHAIN_OXPHOS_S	0,042	0,353	-0,880	-14,466	
GLYOXYLATE_METABOLISM	0,003	0,171	-0,979	-16,445	
GLYCOLYSIS_AND_GLUCONEOGENESIS	0,127	0,418	-0,693	-13,473	
PPAR_SIGNALING_PATHWAY	0,005	0,018	-0,752	-2,094	
PPARALPHA_PATHWAY	0,064	0,432	-0,966	-12,888	
NAD_METABOLISM_SIRTUINS_AND_AGING	0,406	0,731	-0,779	-10,451	
NAD_METABOLISM_	0,965	1,000	-0,328	-0,639	
FOXA2_PATHWAY	0,621	0,866	-0,538	-0,885	
FATTY_ACYL_COA_BIOSYNTHESIS	0,001	0,059	-0,939	-20,775	Reactome
ABC_TRANSPORTER_DISORDERS	0,005	0,133	-0,737	-1,877	
NUCLEAR_RECEPTOR_TRANSCRIPTION_PA	0,009	0,120	-0,513	-18,674	
ABC_FAMILY_PROTEINS_MEDIATED_TRANS	0,013	0,133	-0,737	-17,999	
SYNTHESIS_OF_VERY_LONG_CHAIN_FATTY	0,007	0,121	-0,913	-17,999	
HEDGEHOG_LIGAND_BIOGENESIS	0,004	0,179	-0,854	-17,229	
FATTY_ACID_METABOLISM	0,006	0,177	-0,598	-17,123	
RAF_INDEPENDENT_MAPK1_3_ACTIVATION	0,025	0,173	-0,680	-16,959	
SIGNALING_BY_NOTCH3	0,016	0,245	-0,867	-16,342	
ATF6_ATF6_ALPHA_ACTIVATES_CHAPERON	0,018	0,252	-0,841	-16,255	
ACTIVATED_NTRK2_SIGNALS_THROUGH_PI	0,020	0,262	-0,940	-15,854	
RHO_GTPASES_ACTIVATE_PKNS	0,051	0,289	-0,428	-15,524	
ACTIVATION_OF_GENE_EXPRESSION_BY_SI	0,036	0,276	-0,490	-1,551	
PKA_ACTIVATION_IN_GLUCAGON_SIGNALL	0,066	0,314	-0,767	-15,248	
RHO_GTPASES_ACTIVATE_PAKS	0,043	0,318	-0,692	-15,190	
MTOR_SIGNALLING	0,057	0,318	-0,610	-15,093	
RESPIRATORY_ELECTRON_TRANSPORT_AT	0,047	0,315	-0,898	-15,040	
NICOTINAMIDE_SALVAGING	0,036	0,320	-0,888	-14,914	
RESPIRATORY_ELECTRON_TRANSPORT	0,042	0,328	-0,898	-14,848	
THE_CITRIC_ACID_TCA_CYCLE_AND_RESPI	0,024	0,320	-0,898	-14,825	
COMPLEX_I_BIOGENESIS	0,050	0,334	-0,898	-14,703	
PCP_CE_PATHWAY	0,041	0,328	-0,882	-14,702	
ENERGY_DEPENDENT_REGULATION_OF_MT	0,045	0,350	-0,862	-14,122	
NOTCH3_INTRACELLULAR_DOMAIN_REGUL	0,075	0,371	-0,866	-13,999	
MITOCHONDRIAL_PROTEIN_IMPORT	0,068	0,387	-0,625	-13,879	
RHO_GTPASES_ACTIVATE_CIT	0,125	0,394	-0,691	-1,372	
RHO_GTPASES_ACTIVATE_ROCKS	0,102	0,390	-0,691	-13,712	
MITOCHONDRIAL_TRANSLATION	0,097	0,397	-0,828	-13,663	
SENESCENCE_ASSOCIATED_SECRETORY_P	0,147	0,393	-0,500	-13,658	
PEROXISOME	0,003	0,000	-0,871	-2,379	KEGG
PPAR_SIGNALING_PATHWAY	0,003	0,010	-0,752	-2,082	
FATTY_ACID_METABOLISM	0,003	0,048	-0,913	-1,838	
PROTEASOME	0,043	0,344	-0,882	-1,479	
INSULIN_SIGNALING_PATHWAY	0,043	0,427	-0,298	-1,420	
GLYOXYLATE_AND_DICARBOXYLATE_MET	0,003	0,379	-1,000	-1,343	
GLYCINE_SERINE_AND_THREONINE_METAB	0,018	0,469	-0,992	-1,305	
PHENYLALANINE_METABOLISM	0,048	0,475	-0,979	-1,297	
TYROSINE_METABOLISM	0,054	0,495	-0,979	-1,286	
HEDGEHOG_SIGNALING_PATHWAY	0,184	0,519	-0,908	-1,210	
TAURINE_AND_HYPOTAURINE_METABOLIS	0,230	0,548	-0,872	-1,169	
LYSINE_DEGRADATION	0,289	0,549	-0,592	-1,156	
GALACTOSE_METABOLISM	0,272	0,540	-0,692	-1,153	
FRUCTOSE_AND_MANNOSE_METABOLISM	0,307	0,583	-0,513	-1,117	
CIRCADIAN_RHYTHM_MAMMAL	0,333	0,603	-0,484	-1,102	
GLYCOSYLPHOSPHATIDYLINOSITOL_GPI_A	0,331	0,630	-0,635	-1,083	
GLYCOLYSIS_GLUCONEOGENESIS	0,581	0,736	-0,692	-0,934	
BIOSYNTHESIS_OF_UNSATURATED_FATTY_	0,687	0,881	-0,498	-0,821	
