# Supplementary material for: A MiR181/Sirtuin1 regulatory circuit modulates drug response in biliary cancers
Source: Clin Exp Med. 2024 Apr 10;24(1):74. doi: 10.1007/s10238-024-01332-0 (PMC11006774; doi:10.1007/s10238-024-01332-0)
Supplement: Supplementary file 7 — Supplementary file7 (PDF 68 KB) [file 10238_2024_1332_MOESM7_ESM.pdf]

Table S5. Functional annotation of target genes negatively regulated by miRNA 181c/d in BTC

Functional Category	Genes in list	interaction	interaction score
HALLMARK ADIPOGENESIS	CD302	SIRT1 (pp) DICER1; CD302 (pp) DICER1	0.727
	PRDX3	SIRT1 (pp) PPARGC1A; PPARGC1A (pp) PRDX3	0.460
HALLMARK ANDROGEN RESPONSE	SORD	SIRT1 (pp) CAT; CAT (pp) SORD	0.858
HALLMARK HYPOXIA	ERO1A	SIRT1 (pp) ARNTL; ERO1L (pp) ARNTL	0.459
	ETS1	SIRT1 (pp) TP53; TP53 (pp) ETS1	0.999
	KLF7	SIRT1 (pp) PPARG; PPARG (pp) KLF7	0.438
	PAM		
	TGFBI	SIRT1 (pp) MMP2; MMP2 (pp) TGFBI	0.615
	PAM	SIRT1 (pp) TP53; PAM (pp) FAU; RPS27A (pp) FAU	0.999
HALLMARK MTORC1 SIGNALING	ERO1A	SIRT1 (pp) ARNTL; ERO1L (pp) ARNTL	0.459
	SORD	SIRT1 (pp) CAT; CAT (pp) SORD	0.858
HALLMARK MYC TARGETS	PRDX3	SIRT1 (pp) PPARGC1A; PPARGC1A (pp) PRDX3	0.460
	SORD	SIRT1 (pp) CAT; CAT (pp) SORD	0.858
	SSB	SIRT1 (pp) MDM2; MDM2 (pp) SSB	0.680
HALLMARK OXIDATIVE PHOSPHORYLATION	PRDX3	SIRT1 (pp) PPARGC1A; PPARGC1A (pp) PRDX3	0.460
KEGG PPAR SIGNALING_PATHWAY	PDPK1	SIRT1 (pp) FOXO3; PDPK1 (pp) FOXO3	0.792
REACTOME ABC TRANSPORTER_DISORDERS	ERO1A	SIRT1 (pp) ARNTL; ERO1L (pp) ARNTL	0.459
REACTOME METABOLISM OF AMINO ACIDS AND DERIVATIVES	GLS	SIRT1 (pp) TP53; TP53 (pp) GLS	0.999
	RIMKLB	SIRT1 (pp) MDM2; FOLH1 (pp) MDM2; FOLH1 (pp) RIMKLB	0.787
	SECISBP2	SIRT1 (pp) MDM2; MDM2 (pp) RPL30; SECISBP2 (pp) RPL30	0.787
REACTOME NUCLEAR RECEPTOR TRANSCRIPTION PATHWAY	ESR1	SIRT1 (pp) ESR1	0.909
	NR2C2	SIRT1 (pp) HDAC3; HDAC3 (pp) NR2C2	0.524
