# Supplementary material for: A MiR181/Sirtuin1 regulatory circuit modulates drug response in biliary cancers
Source: Clin Exp Med. 2024 Apr 10;24(1):74. doi: 10.1007/s10238-024-01332-0 (PMC11006774; doi:10.1007/s10238-024-01332-0)
Supplement: Supplementary file 8 — Supplementary file8 (DOCX 27 KB) [file 10238_2024_1332_MOESM8_ESM.docx]

**Supplemental data**

**Supplemental data include 2 supplemental figures and 5 tables.**

**Supplementary Figure 1.** *MiR-181c and -181d modulate the response to therapy in BTC*

**A)** Violin plots showing the expression (CPM log) of miR-181c and miR-181d in 62 BTC patients grouped on the basis of the primary tumor site in intra- (iCCA), extra-haepatic (eCCA) cholangiocarcinoma and gallbladder cancer (GBC); the upper/lower quartile and the median were indicated; p< 0.05.

**B)** Kaplan–Meier estimates Overall survival (OS) of patients responders and resistant to chemotherapy. Patients were stratified by using RECIST criteria. Pairwise log-rank test was used to analyse the survival; p< 0.0001.

**C)** Violin plots showing the expression (Log-CPM) of miR-181c and miR-181d in BTC patients grouped on the basis of the CA19-9 plasma levels; CA19-9 plasma levels of 37 U/mL were considered as cut-off value; the upper/lower quartile and the median were indicated; p< 0.05.

**D)** Bar-plot depicting the expression (Log-CPM) of miRNA-181 family members in BTC and Cholangiocytes cell lines.

**E)** QRT-PCR analysed miR-181c, miR-181d in EGI, EGI-DR and RBE cells transfected with miR-181c and -181d mimic and CTR. Statistical analyses were conducted by unpaired t test or one-way ANOVA with Tukey’s post hoc analysis. *p < 0.01; **p < 0.001; ***p < 0.0001.

**F)** Flow cytometry analysis of apoptosis in RBE and EGI cells transfected with miR-181c and -181d mimic and CTR for 48h. The degree of apoptosis has been determined by annexin V/PI staining. Representative histograms from a typical experiment are shown.

**G-H)** Pearson correlation analysis for miR-181c **(F)** and miR-181d **(G)** expression and the GI_50_ values for Gem/Cis in the BTC cell lines. Data are plotted in log-log scale (Log base 10). Red dots indicate Gem/Cis-resistant cells.

**I)** Flow cytometry analysis of apoptosis in EGI and EGI-DR cells transfected with miR-181c and -181d mimic and CTR and treated with Gem/Cis. The degree of apoptosis has been determined by annexin V/PI staining. Representative histograms from a typical experiment are shown.

**J)** QRT-PCR analysed miR-181c, miR-181d and SIRT1 mRNA expression in Intra-haepatic (Intra); Extra-haepatic (Extra); Gallbladder/Ampulla of Vater (BG/AV) BTC cell lines and normal cholangiocytes (Normal). Expression data are plotted in Fold Change (FC). The horizontal bar within each box indicates the median. The data are means +/- SD of three independent experiments. Statistical analyses were conducted by two-way ANOVA. *p < 0.01.

**Supplementary Figure 2.** *MiR-181c/d Modulate Key Signaling Transcriptional Networks in BTC*

**A-B)** Heatmap demonstrating log relative expression level of differentially expressed genes between the groups of patients in which miRNAs were UP-regulated and orange the DOWN-regulated. Cell colors (blue-white-orange-red gradient) correspond the binary logarithm of the ratio of the expression level in a current sample to the average level across all the samples (per each gene). Cluster analysis grouped samples and differentially expressed genes (DEGs) according to similarity in expression. DEGs are in rows and samples in columns. The DEGs clustering tree is indicated on the left. To create the heatmap we converted the read counts into log2-counts-per-million (logCPM) values. **C-E)** Representative GSEA plots of MTORC1 signalling, Fatty Acid Metabolism, ABC family gene signatures. Only the gene sets that showed the normalized enrichment score (NES > 2.0) and false discovery rate (FDR) q value < 5% were reported. Representative Heatmaps of differentially expressed genes constituting the leading-edge subsets within the gene sets shown. Each row represents a gene, and each column represents the Median Fold Change (Log) of miRNA-181c/d UP-regulated patients vs DOWN-regulated. **F-G)** Heatmap of the coefficients of the Pearson correlation matrix between the miR-181c **(F)** and miR-181d **(G)** expression, and target gene expression values for BTC patients. **H-J)** Gene set enrichment analysis (GSEA) with compiled modules from Wiki-Pathways, Reactome and KEGG in MSigDB. Dot plots of GSEA results illustrated gene network associated with miRNA-181c/d expression in BTC patients. The figures show the significant top 15 positively and the top 15 negatively enriched GO terms, based on co-expressed genes. Dot color indicates the Enrichment Score (ES). Dot size indicates the p value (-Log10 pvalue).

**Table S1**

Demographic and clinical characteristics of BTC patients.

**Table S2**

Differentially expressed genes p-value filtered (<0.05) in BTC patients grouped as miR-181c and miR-181d UP and DOWN expression.

**Table S3**

Pearson's correlation of miR-181c and miR-181d and predicted target genes (filtered for gene expression's p-value < 0.05) in BTC patients.

**Table S4**

GSEA querying Molecular Signature database (MSigDB) of DE target genes in BTC patients expressing high miRNA-181c/d versus low, using hallmark, wikipathways, reactome and KEGG pathways.

**Table S5**

Functional annotation of target genes negatively regulated by miRNA 181c/d in BTC
